# Synergistic Ferroptosis–Immunotherapy Nanoplatforms: Multidimensional Engineering for Tumor Microenvironment Remodeling and Therapeutic Optimization

**DOI:** 10.1007/s40820-025-01862-6

**Published:** 2025-09-02

**Authors:** Xiao Wei, Yanqiu Jiang, Feiyang Chenwu, Zhi Li, Jie Wan, Zhengxi Li, Lele Zhang, Jing Wang, Mingzhu Song

**Affiliations:** 1 School of Preclinical Medicine, Chengdu University, Chengdu, 610106 People’s Republic of China; 2 Section of Molecular Dermatology, Medical Faculty Mannheim, Heidelberg University, Heidelberg, 69117 Germany

**Keywords:** Ferroptosis–immunotherapy, Nanoplatforms, Tumor microenvironment, Synergistic strategies, Nanocarrier design

## Abstract

Emerging ferroptosis–immunotherapy strategies, integrating functionalized nanoplatforms with ferroptosis-inducing agents and immunomodulatory therapeutics, demonstrate significant potential in managing primary, recurrent, and metastatic malignancies. Mechanistically, ferroptosis induction not only directly eliminates tumor cells but also promotes immunogenic cell death (ICD), eliciting damage-associated molecular patterns (DAMPs) release to activate partial antitumor immunity. However, standalone ferroptosis therapy fails to initiate robust systemic antitumor immune responses due to inherent limitations: low tumor immunogenicity, immunosuppressive microenvironment constraints, and tumor microenvironment (TME)-associated physiological barriers (e.g., hypoxia, dense extracellular matrix). To address these challenges, synergistic approaches have been developed to enhance immune cell infiltration and reestablish immunosurveillance, encompassing (1) direct amplification of antitumor immunity, (2) disruption of immunosuppressive tumor niches, and (3) biophysical hallmark remodeling in TME. Rational nanocarrier design has emerged as a critical enabler for overcoming biological delivery barriers and optimizing therapeutic efficacy. Unlike prior studies solely addressing ferroptosis or nanotechnology in tumor therapy, this work first systematically outlines the synergistic potential of nanoparticles in combined ferroptosis–immunotherapy strategies. It advances multidimensional nanoplatform design principles for material selection, structural configuration, physicochemical modulation, multifunctional integration, and artificial intelligence-enabled design, providing a scientific basis for efficacy optimization. Moreover, it examines translational challenges of ferroptosis–immunotherapy nanoplatforms across preclinical and clinical stages, proposing actionable solutions while envisioning future onco-immunotherapy directions. Collectively, it provides systematic insights into advanced nanomaterial design principles and therapeutic optimization strategies, offering a roadmap for accelerating clinical translation in onco-immunotherapy research.

## Highlights

- First systematic integration: This work presents the first comprehensive outline of the synergistic potential of nanoparticle-enabled ferroptosis–immunotherapy strategies against malignancies, moving beyond studies solely focusing on ferroptosis induction or standalone nanotherapeutics in cancer.
- Multidimensional nanoplatform design: Establishes advanced design principles for functionalized nanoplatforms, including rational material selection, structural configuration, physicochemical modulation, multifunctional integration, and AI-enabled design, to overcome tumor microenvironment barriers and optimize ferroptosis–immunotherapy efficacy.
- Translational focus & AI integration: Provides a critical analysis of translational hurdles for ferroptosis–immunotherapy nanoplatforms across preclinical and clinical development, proposing actionable solutions while pioneering the integration of artificial intelligence into future nanoplatform design and onco-immunotherapy direction.

## Graphical abstract

**Figure:**
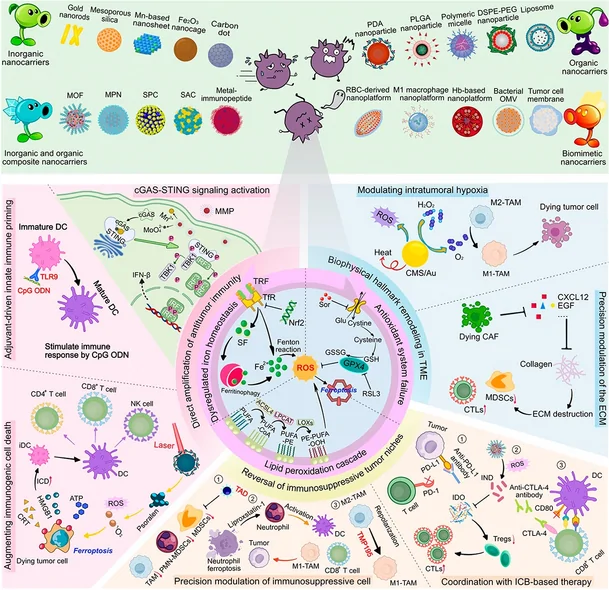

## Introduction

Recent advances in tumor immunotherapy have transformed oncology by harnessing sustained systemic immunity to eliminate malignancies and suppress metastasis [1, 2]. However, the immunosuppressive tumor microenvironment (TME) diminishes tumor immunogenicity, leading to suboptimal response rates and compromised therapeutic efficacy [3]. Emerging evidence highlights ferroptosis—a regulated cell death mechanism—as a promising strategy to enhance tumor immunogenicity and stimulate antitumor immunity while selectively eradicating malignant cells.

Ferroptosis is mechanistically driven by three core pathways: (I) **Dysregulated iron homeostasis**: Intracellular accumulation of redox-active Fe^2+^ in the labile iron pool (LIP) initiates ferroptosis. Strategies to amplify LIP Fe^2+^ include iron-based nanocarriers for iron donation, salinomycin (Sal)-mediated upregulation of transferrin receptors to enhance transferrin-dependent iron uptake [4], and autophagic ferritin degradation promoted by dihydroartemisinin (DHA) or spiked surface nanoparticles [5–7]. Elevated Fe^2+^ catalyzes Fenton reactions, generating hydroxyl radicals (·OH) that drive peroxidation of polyunsaturated fatty acid-containing phospholipids (PUFA-PLs), compromising membrane integrity and inducing ferroptosis [8]. (II) **Lipid peroxidation cascade**: The peroxidation susceptibility of bis-allylic methyl-containing PUFAs dictates ferroptosis progression [9]. Nanoparticle delivery of arachidonic acid (AA) or adrenic acid increases cellular PUFA reservoirs. Radiation exposure or NF2-YAP pathway inhibition elevates acyl-coA synthetase long-chain family member 4 (ACSL4) expression, promoting PUFA-PL biosynthesis and membrane incorporation [10]. Subsequent iron-dependent Fenton reactions or lipoxygenase (LOX) activity converts PUFA-PLs to phospholipid hydroperoxides, executing ferroptosis [11, 12]. (III) **Antioxidant system failure**: Glutathione peroxidase 4 (GPX4), essential for lipid peroxide (LPO) detoxification, is inactivated through multiple mechanisms [13]. Covalent inhibition by RSL3 directly blocks GPX4 activity [14]. Erastin and sorafenib (SRF) suppress system Xc^−^-mediated cystine uptake, depleting glutathione (GSH) and indirectly inactivating GPX4 [15]. This dual inactivation disrupts redox homeostasis, permitting lethal LPO accumulation and plasma membrane rupture.

Ferroptosis-based therapy exerts dual antitumor effects by directly eliminating tumor cells and enhancing immunogenicity via immunogenic cell death (ICD), which activates robust antitumor immunity. Ferroptotic cells release tumor-associated antigens (TAAs) and damage-associated molecular patterns (DAMPs; high-mobility group box 1 (HMGB1), surface-exposed calreticulin (CRT), and extracellular adenosine triphosphate (ATP)), promoting dendritic cell (DC) maturation and subsequent T cell-mediated antitumor immunity [16, 17]. Activated CD8^+^ T cells amplify ferroptosis sensitivity through interferon-γ (IFN-γ) secretion, which simultaneously suppresses system Xc^−^-mediated GSH synthesis and upregulates ACSL4 to enhance PUFA-phospholipid biosynthesis [18]. Furthermore, modulating ferroptosis in immunosuppressive cells reverses their pro-tumor functions while attenuating immunosuppressive activity. Notably, certain ferroptosis inducers induce either ferroptosis in M2 macrophages or their repolarization into tumor-suppressive M1 phenotypes [19]. These M1 macrophages generate hydrogen peroxide (H_2_O_2_) to intensify intratumoral Fenton reactions, creating a self-reinforcing ferroptosis cycle [20]. The integration of ferroptosis inducers with immunotherapies represents a promising strategy to remodel the TME and mobilize immune effectors, potentially enabling sustained tumor control and improved therapeutic outcomes.

Emerging nanomaterials are actively explored for combined ferroptosis–immunotherapy to address challenges in biocompatibility, systemic retention, and targeting precision of therapeutic agents. Functionalized nanoplatforms demonstrate multifunctional advantages in this synergistic approach: (1) Diverse nanocarrier architectures: Nanomaterials are categorized as organic, inorganic, composite, or biomimetic based on composition. Organic systems like liposomes offer dual encapsulation of hydrophilic/hydrophobic drugs with inherent biocompatibility [21]. Inorganic counterparts such as carbon dots (CDs) exhibit enzyme-mimicking catalytic activity and low environmental impact [22]. Iron oxide nanoparticles enable magnetic targeting and magnetic resonance imaging (MRI)-guided therapy [23]. Metal–organic frameworks (MOFs) and metal–phenolic networks (MPNs) leverage high surface area and Fe^2+^ stabilization to potentiate Fenton reactions [24, 25]. Biomimetic nanoplatforms derived from tumor cell membranes or bacterial outer membrane vesicles (OMVs) combine immune evasion with homologous targeting [26, 27]. (2) Targeted delivery and stimuli-responsive release: Functionalization strategies enhance tumor specificity through ligand–receptor interactions (e.g., CD44/folic acid (FA)-mediated targeting) [28]. TME-responsive systems exploit acidic pH, elevated GSH, or enzyme overexpression for spatiotemporally controlled drug release. Multimodal imaging integration—via fluorescent tags, photothermal agents, or magnetic components—enables real-time therapeutic monitoring [29, 30]. (3) Immunomodulation and therapeutic amplification: Engineered nanoplatforms synergize ferroptosis with ICD induction. When combined with phototherapies (photothermal therapy (PTT)/photodynamic therapy (PDT)) or chemotherapeutic agents (Doxorubicin (DOX)/DHA), they enhance tumor immunogenicity and immune cell infiltration, potentiating immune checkpoint inhibitor efficacy [31]. Nanoparticles delivering immunomodulators activate immune cells and modulate the TME, for example through co-delivery of immune adjuvants like cytosine-phosphorothioate-guanine oligodeoxynucleotides (CpG ODNs) enhancing antigen presentation and stimulating T-cell activation [32]. Oxygen-generating nanoreactors alleviate tumor hypoxia to polarize M2 to M1 macrophages and amplify reactive oxygen species (ROS)-mediated ferroptosis [33].

**Scheme 1:**
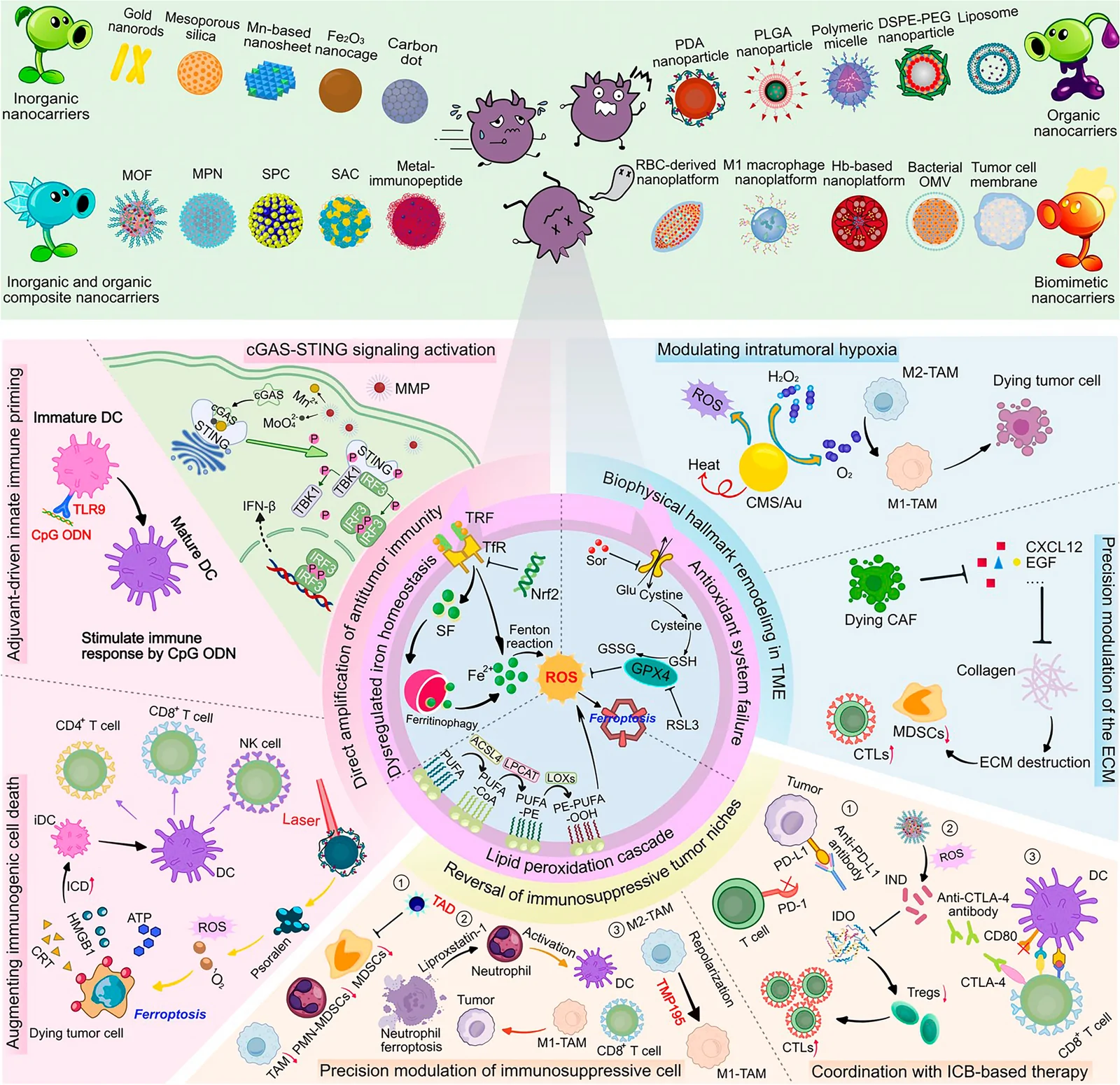
Mechanistic overview of engineered nanoplatforms for synergistic ferroptosis–immunotherapy: integration of tumor microenvironment modulation, immunogenic cell death, and immune response activation

This work systematically classifies nanocarriers for ferroptosis–immunotherapy into four categories by composition: organic (e.g., liposomes, polymeric micelles/nanoparticles), inorganic (e.g., iron oxide nanoparticles, mesoporous silica), composite (e.g., MOFs, MPNs), and biomimetic (e.g., tumor cell membrane-coated nanocarriers), listing representative systems. Upon tumor-targeted delivery, these nanoparticles dysregulate iron homeostasis, trigger lipid peroxidation cascades, and collapse antioxidant defenses, forcing tumor cells into ferroptosis. This subsequently elicits ICD and activates antitumor immunity. To amplify immunotherapy, we developed synergistic strategies: directly amplifying antitumor immunity via cGAS-STING activation, co-delivered immune adjuvants, or augmented ICD; reversing immunosuppressive niches by modulating immunosuppressive cells and coordinating with immune checkpoint blockade (ICB)-based therapy; and remodeling the biophysical hallmark of TME through precise hypoxia modulation and extracellular matrix (ECM) degradation, boosting overall immunotherapeutic efficacy (Scheme 1).

While existing reviews have examined ferroptosis in immunotherapy or nanoparticle-triggered ferroptosis in cancer therapy, this constitutes the first comprehensive review focusing on nanoparticle-mediated ferroptosis–immunotherapy synergistic mechanisms, introducing a novel framework for designing nanocarriers specifically for ferroptosis–immunotherapy. This review systematically outlines design principles for ferroptosis–immunotherapy-integrated nanocarriers, focusing on carrier classification, structural engineering, and physicochemical property-driven drug delivery optimization. Their multifunctional roles in ferroptosis induction, molecular imaging, active targeting, and stimuli-responsive release are critically analyzed. After exploring the design of artificial intelligence (AI)-enabled smart nanoplatform, we further detail advanced strategies to amplify therapeutic synergy, including (1) enhancing antitumor immunity via ICD promotion, (2) disrupting immunosuppressive pathways (e.g., programmed cell death protein 1 (PD-1)/programmed cell death-ligand 1 (PD-L1) axis), and (3) remodeling the TME through hypoxia alleviation or extracellular matrix modulation. Current challenges—such as carrier biodegradation kinetics, off-target effects, and clinical scalability—are discussed with proposed solutions leveraging surface engineering and computational design. Looking forward, nanotechnology tailored for spatiotemporal control of ferroptosis–immunotherapy cross-talk promises to enhance therapeutic precision, accelerate clinical translation, and revolutionize oncology paradigms.

## Meticulous Design of Nanocarriers for Ferroptosis–Immunotherapy: A Multidimensional Insight

Current advancements in ferroptosis–immunotherapy focus on engineering functional nanoplatforms with precise targeting and tunable drug release profiles. Critical considerations for optimizing anticancer nanotherapeutics include: (1) Customization of nanocarrier types and components to meet personalized therapeutic demands; (2) Strategic modulation of physicochemical parameters (size, surface charge, morphology, amphiphilicity) to govern stability, biodistribution, and cellular uptake; (3) Multifunctional integration of tumor-targeting ligands, microenvironment-responsive release mechanisms, and imaging modalities (e.g., MRI/fluorescence) within a unified system; (4) AI-enabled smart design including aiding nanomaterial screening, predicting in vivo processes, and evaluating nanoparticle efficacy. This section systematically discusses how structural engineering (such as nanocarrier classifications and their advantages/limitations) and functional synergies in nanocarriers enhance ferroptosis induction, molecular imaging precision, active targeting efficiency, and stimulus-responsive behaviors. Relevant descriptions are summarized in Tables 1, 2 and 3. By establishing these design frameworks, this analysis aims to accelerate the rational development of nanomedicines for clinically translatable ferroptosis–immunotherapy strategies.

**Table 1:** Engineered nanoplatforms: compositional/structural features enabling ferroptosis–immunotherapy synergy

Category	Nano-formulation	Structural constituent	Size (nm)	Zeta potential (mv)	Morphology	Therapeutic agent	Drug-loading mode	References
Organic nanocarrier	Polymeric nanoparticle	PLGA/DSPE-PEG_2k_/PFOB	130.8	− 33	Sphere	Gambogic acid	Hydrophobic interaction	[84]
		Butyrate/PLGA-lipid	130	− 20	Sphere	Sor/Sal	Physical loading	[85]
	Polymeric micelle	PEG-b-PDPA-r-PPa/mPEG-b-PDPA-r-GC/PBE	~ 100	–	Sphere	RSL-3	*π*–*π* stacking/hydrophobic interaction	[42]
		PS-b-PAA/MPTMS/PEG	120	− 22.8	Sphere	SAS/DOX	Hydrophobic interaction	[44]
	Liposome	DSPE-PEG_2k_/DOPA/DOPC/BC-LNPs	108.44	− 31.3 ± 1.04	Sphere	Paclitaxel/alendronate	Hydrophobic interaction/Coordination	[86]
		DSPE-TK-PEG_2k_	115.71 ± 11.97	− 40 ~ − 30	Sphere	Ce6/ML385/Arg	Hydrophobic interaction	[37]
Inorganic nanocarrier	Mn-doped layered nanosheet	Mn/LDH	58 ± 1	41 ± 3	Sheet	IFN-γ	Electrostatic interaction	[57]
	Hollow mesoporous copper sulfide nanoparticle	Cu_2−X_S/NH_2_-PEG	110	–	Sphere	GOx/CaCO_3_	Covalent conjugation/Sedimentation	[61]
	MnMoO_4_ nanodot	MnMoO_4_/DSPE-PEG_5k_	5	–	Dot	Mn^2+^/MoO_4_^2−^	–	[62]
	Gold nanoparticle	Au core/thiol-PEG-amine	~ 100	~ 0	Sphere	miR-21-3p	Covalent conjugation	[87]
	Bi_2_Fe_4_O_9_ nanosheet	Bi_2_Fe_4_O_9_	60.05	− 14.2 ± 2.4	Sheet	–	–	[88]
	Up-conversion SiO_2_ nanocomposite	SiO_2_/NaYF_4_:Yb,Er@NaYF_4_	~ 100	–	Sphere	Ce6/BSO	Physical adsorption	[89]
	CaCO_3_/Mn^2+^@SiO_2_ nanocomposite	CaCO_3_/Mn^2+^/TEOS	211	− 14.9	Sphere	Ca^2+^/Mn^2+^	Cation exchange	[69]
	Tetrapod FePd nanocrystal	FePd/DA/PVP	110	–	Tetrapod spiky-like	Fe^2+^	Coordination	[5]
	Hollow Cu_2_WS_4_ nanosphere	Cu_2_WS_4_/PEG/PVP	80	− 7.23	Sphere	–	–	[64]
	Cu-based carbon dot	FA/Genistein/Cu^2+^	7.1 ± 1.9	–	Dot	Cu^2+^	H-bond coordination	[65]
Composite nanocarrier	MOF	2-aminoterephthalic acid/Fe^2+^	245	–	Polyhedron	MnO_2_/GOx	Covalent conjugation/Chemical deposition	[90]
	Metal–phenolic network	Mn^2+^/Fe^3+^/TA	168	− 27.6	Irregular morphology	DNAzyme	Electrostatic interaction	[91]
	Metal–polyphenol coordinated nanoparticle	BSA/EGCG/MnO_2_	190 ± 10	–	Sphere	Mn^2+^/Sorafenib	Coordination/physical loading	[92]
	Porous bimetallic nanozyme	FeCo/Fe-Co DAzyme/BSA	200	− 23	Sphere	Phospholipase A_2_/LOX	Physical loading	[75]
	Coordination polymer nanofiber	Gallic acid/aspirin/Fe^2+^/PVP	300	–	Fiber shape	Aspirin/Fe^2+^	–	[93]
	Anti-PD-L1 peptide-Fe nanocomplex	Fe^3+^/TPP/APP–spermidine–dextran	50	22.4	–	Spermidine/Fe^3+^	Covalent conjugation/coordination	[94]
	UA NP-carbon dot conjugate	Ursolic acid	133.5 ± 1.2	− 21.2 ± 0.8	Sphere	Ursolic acid/carbon dot	H-bond interaction	[95]
Biomimetic nanocarrier	Platelet membrane-derived magnetic nanoparticle	Platelet/Fe_3_O_4_	268.9 ± 8.9	− 22.1 ± 0.9	Sphere	SAS/Fe^2+^	Physical loading	[82]
	Erythrocyte membrane-coated nanoparticle	Erythrocyte membrane/TCPP/Fe^3+^	298	–	Fusiform	AA/Fe^3+^	Physical adsorption	[81]
	Cancer cell membrane-coated nanoparticle	Cancer cell membrane/MSN/Mn^2+^	125.90 ± 2.52	− 39.83 ± 1.46	Sphere	Pt (IV)	Covalent conjugation	[96]
	OMV-coated nanoparticle	OMV/MSN/Fe^3+^	150	− 8.5	Sphere	DHA/Fe^3+^	Physical adsorption	[80]

**Table 2:** Comparative analysis of nanocarrier platforms for ferroptosis–immunotherapy: advantages and limitations

Category	Advantages	Disadvantages
Organic nanocarriers	* High biocompatibility and biodegradability	* Limited drug-loading capacity
	* Dual drug encapsulation capacity (hydrophilic/hydrophobic) [34]	* Poor structural stability under physiological conditions [97]
	* Amenable to functional surface modification for targeted delivery [39]	* Complex functionalization required to overcome tumor microenvironment (TME) barriers
Inorganic nanocarriers	* High drug-loading capacity [53]	* Potential long-term toxicity and biopersistence [98]
	* Intrinsic catalytic or enzyme-mimicking activity for ferroptosis induction [66]	* Lower biocompatibility [97]
	* Compatibility with multimodal imaging (e.g., MRI, PAI, US)	* Susceptible to immune recognition without surface shielding or hybridization
	* Durable and structurally stable under physiological conditions	
Composite nanocarriers	* Combines functionalities from multiple components	* Complex synthesis and potential stability concerns
	* High surface area and tunable porosity facilitate ROS diffusion and drug loading [71]	* Less explored for clinical use (requires further validation of scalability and safety) [98]
	* Facilitates multimodal therapeutic integration	* Risk of metal ion leakage if improperly stabilized
Biomimetic nanocarriers	* Enhanced immune evasion and prolonged circulation via native membrane proteins [78]	* Potential batch-to-batch variability in membrane source and properties
	* High target specificity through homologous targeting [78]	* May exhibit limited drug-loading capacity compared to synthetic carriers

**Table 3:** Engineered nanoformulations: functional versatility in ferroptosis–immunotherapy platforms

Nano-formulation	Structural constituent	Ferroptosis induction	Molecular imaging	Active targeting	Stimulus response	Tumor model	References
Liposome	DSPE-PEG_2k_-SH/DOTAP/DPPC/AuNPs	As_2_O_3_	PFH/USI	cRGD/tumor cell targeting	US/pH	Hepatocellular carcinoma	[111]
	DPPC/NHS/DSPE-PEG_2k_	R162	IR780/FI, PAI	TPP/mitochondrial targeting	US	Breast cancer	[112]
Polymeric nanoparticle	TCPP-TK-PEG-PAMAM	Hf^4+^/siHIF-1α	–	FA/tumor cell targeting	ROS	Breast cancer	[49]
	Benzimidazole–cyclodextrin	SRF	–	Transferrin/tumor cell targeting	pH	Breast cancer	[113]
	PLGA	RSL-3/UMF	PFH/USI UMF/MRI, PAI	VGB3 peptide/tumor cell targeting	NIR	Breast cancer	[114]
Polymeric micelle	PDBA	RSL-3/DHA	IR780/FI	–	pH/ROS	Pancreatic ductal adenocarcinoma (PDAC)	[115]
Semiconducting iron nano-chelate	Semiconductor polymer chelating Fe^3+^/PEG	Fe^3+^	SPC/FI	–	pH	Breast cancer	[43]
Gold nanorod	Gold nanorod/chitosan/12-mer peptide	Gold nanorod	–	12-mer peptide/tumor cell targeting	–	Acute myeloid leukemia	[55]
Mesoporous manganese oxide	Regenerated silk fibroin/MnO_*x*_	Mn^2+^/ICG derivative	–	CD44/tumor cell targeting	US	Colon cancer	[116]
Single-crystal Fe nanoparticle	Single-crystal Fe/Fe_3_O_4_/iRGD peptide	Fe	–	iRGD/tumor cell targeting	pH	Hepatocellular carcinoma	[117]
Fe_2_O_3_ nanoparticle	DMSA/Fe_2_O_3_	Fe_2_O_3_/DOX	Fe_2_O_3_/MRI	–	GSH/pH/ROS	Breast cancer	[118]
FePd nanocrystal	FePd/PVP	FePd	FePd/PAI	–	–	Breast cancer	[5]
Cu_2−x_Se/ZIF-8 heterostructure	Cu_2−x_Se/ZIF-8/PEG-FA	Erastin/Cu^+^/Cu^2+^	–	FA/tumor cell targeting	–	Breast cancer	[33]
Arsenene–Iridium nanosheet	As/IrFN/PDA	IrFN	–	IrFN/mitochondria targeting	–	PDAC	[74]
Semiconducting polymer nano-PROTAC	PROTAC/PFODBT/ferrocene/DSPE-TK-PEG	Ferrocene	PFODBT/FI	–	US	Colon cancer	[119]
Metal–pyrimidine network	Zn^2+^/5-Fu	Zn^2+^	5-Fu/MRI	–	ATP/pH	Colon cancer	[120]
Bimetallic MOF	Telmisartan-PEG_2k_/FeCo	Panobinostat/Fe^3+^/Co^3+^	–	AT1R/CAFs targeting, tumor cell targeting	–	Breast cancer	[121]
MOF	Fe^3+^/TA/BSA	Triptolide/Fe^3+^	–	FA/tumor cell targeting	–	Melanoma	[122]
Biomimetic nanocarrier	Hb/MMP2-responsive peptide/SRF	SRF/Fe	–	Hb/homologous targeting	Enzyme	Breast cancer	[83]
	Platelet membrane/Fe_3_O_4_	SAS	–	Platelet membrane/homologous targeting	–	Breast cancer	[82]
	Leukocyte membrane/Fe_3_O_4_	Fe^2+^	Fe_3_O_4_/MRI	Fe_3_O_4_/Magnetic targeting, leukocyte membrane/homologous targeting	pH	Breast cancer	[123]
	M1 macrophage membrane/DMSN	Fe^3+^	–	M1 macrophage membrane/homologous targeting	pH	Osteosarcoma	[79]
	Tumor cell membrane/bacterial-derived VNP	SAS/Fe	–	Tumor cell membrane/homologous targeting	pH	Breast cancer	[124]

### Nanocarrier Classifications for Ferroptosis–Immunotherapy

#### Organic Nanocarriers

This section analyzes three organic nanoplatforms—liposomes, polymeric micelles, and polymeric nanoparticles—focusing on their structural and functional attributes (Tables 1, 2). Their amphiphilic architectures enable hydrophobic drug encapsulation in their cores while hydrophilic shells improve pharmacokinetic stability in biological environments. These systems demonstrate inherent biocompatibility and biodegradability, minimizing systemic toxicity risks while ensuring efficient drug transport. Such features position them as clinically viable drug delivery systems for precision medicine applications.

##### Liposomes

Liposomes are spherical nanostructures composed of self-assembled amphiphilic molecules, featuring a hydrophilic core surrounded by a lipid bilayer [34]. Their structure enables differential drug loading: hydrophilic drugs partition into the aqueous core through passive diffusion, while lipophilic drugs integrate into the bilayer via hydrophobic interactions. The cationic surface properties facilitate electrostatic adsorption of anionic nucleic acid therapeutics, improving their biological stability [35]. Incorporation of PUFAs in the lipid membrane provides substrates for tumor-specific lipid peroxidation, effectively inducing ferroptosis. Furthermore, liposomal coatings can enhance biocompatibility of inorganic nanoparticles and MOFs while enabling surface functionalization [36]. These unique characteristics have driven the development of liposomal platforms for ferroptosis–immunotherapy combinations. Wang et al. developed cholesterol/1,2-distearoyl-sn-glycero-3-phosphoethanolamine-thioketal-polyethylene glycol 2000 (DSPE-TK-PEG2000) liposomes encapsulating L-arginine (Arg) in the aqueous core, with chlorin e6 (Ce6) photosensitizer and erythroid 2-related factor 2 (NRF2) inhibitor ML385 embedded in the hydrophobic bilayer [37]. Light-triggered Ce6 release combined with Arg metabolites promoted ferroptosis-mediated ICD, while ML385 alleviated immunosuppression through PD-L1 downregulation and myeloid-derived suppressor cell (MDSC) reprogramming. In a separate approach, Gao et al. engineered cationic liposomes using DOTAP, DSPE-PEG2000, cholesterol, and DAPC [38]. The formulation electrostatically complexed immunostimulatory CpG ODNs while utilizing DAPC's dual AA chains to amplify lipid peroxidation. This strategy concurrently induced ferroptosis through system Xc^−^ inhibition and enhanced DC maturation, establishing a synergistic therapeutic loop. Advanced hybrid systems demonstrate additional functionality. A Fe^3+^-terephthalic acid (H_2_BDC) MOF drug carrier encapsulated within liposomes achieved improved biocompatibility while providing anchoring sites for tumor-targeting peptide modifications, effectively suppressing both primary tumor growth and metastatic progression [39].

##### Polymeric Micelles

Polymer micelles, formed by self-assembly of amphiphilic block copolymers, feature a hydrophobic core and hydrophilic shell that synergistically enhance tumor therapeutic delivery [40]. The core effectively encapsulates lipophilic drugs while the hydrophilic corona prevents serum component interactions and immune recognition, prolonging systemic circulation [41]. Tailorable copolymer properties enable optimization of drug-loading capacity and pharmacokinetic profiles for diverse therapeutic agents. Recent advances have engineered sophisticated micellar systems for ferroptosis–immunotherapy combination strategies. Song et al. developed pH-responsive nanomicelles using acid-ionizable PEG-block-poly(2-(diisopropylamino)ethyl methacrylate) (PEG-b-PDPA) copolymers stabilized by phenylboronate ester covalent bonds [42]. The *π*–*π* stacking between phenylboronate ester groups facilitated stable RSL-3 encapsulation in the PDPA core. These nanomicelles demonstrated acid-activated PDT through core protonation while enhancing tumor cell sensitivity to RSL-3-induced ferroptosis via T lymphocyte-mediated IFN-γ secretion. Another innovation involved semiconductor iron-chelating nanomodulators containing thiophene-based Schiff base polymers for Fe^3+^ coordination, modified with PEG side chains [43]. This system achieved spatiotemporal control of ferroptosis induction coupled with M1 macrophage polarization, significantly enhancing effector T-cell infiltration for potent tumor suppression and metastasis inhibition. A hybrid organic–silica micelle incorporating disulfide bonds was designed for GSH-responsive co-delivery of sulfasalazine (SAS) and DOX [44]. Micellar disassembly under reducing conditions enabled simultaneous ferroptosis and pyroptosis induction, promoting DC maturation and cytotoxic T lymphocyte (CTL) activation through enhanced tumor antigen release.

##### Polymeric Nanoparticles

Polymer nanoparticles, constructed from structural polymer subunits, demonstrate precise control over size, surface charge, and multidrug encapsulation capacity, enabling simultaneous delivery of therapeutic agents with enhanced efficiency [45]. Their superior biocompatibility and surface modifiability permit targeted delivery through functionalization, positioning them as promising platforms for ferroptosis–immunotherapy applications [46]. A recent study engineered DOX-loaded DSPE-PEG microcapsules through electrostatic encapsulation, followed by tannic acid (TA)-Fe^3+^ coating [47]. This dual-release system triggered apoptosis (via DOX) and ferroptosis (via Fe^3+^), amplifying oxidative stress to potentiate ICD and synergize with PD-L1 blockade therapy. Poly(lactic-co-glycolic acid) (PLGA), a biodegradable polymer, has been utilized in hybrid core–shell vesicles incorporating iron oxide nanocubes and hydrophobic ascorbic acid through hydrophobic interactions [48]. Poly(amidoamine) (PAMAM) dendrimers leverage their branched architecture for high-capacity drug loading and targeted delivery. Tetra(4-carboxyphenyl)porphyrin (TCPP)-conjugated, FA-functionalized PAMAM nanoparticles effectively delivered HIF-1α-targeting siRNA via electrostatic adsorption, enhancing tumor ferroptosis sensitivity while eliciting systemic antitumor immunity with memory responses [49]. Polydopamine (PDA) systems exhibit pH-responsive behavior in TME. Sun et al. developed dopamine-modified oxaliplatin nanoparticles coated with PDA, incorporating lapatinib via hydrophobic interactions [50]. This nanosystem achieved TME-specific charge reversal for localized ferroptosis induction, CTL activation, and tumor infiltration while maintaining excellent biosafety.

Beyond conventional designs, structurally and functionally advanced polymeric nanoparticles show promise in ferroptosis–immunotherapy. Fan et al. engineered glioblastoma-targeting nanoparticles by conjugating matrix metalloproteinase-2 (MMP-2)-responsive HA-PLGLAG-dEGCG polymers with anti-B7-H3 × CD3 bispecific antibodies [51]. This system enhanced ferroptosis–immunotherapy synergy, significantly improving glioblastoma eradication. Separately, non-metallic covalent organic frameworks (COFs) integrated with aggregation-induced emission (AIE) materials generated dual pyroptosis/ferroptosis inducers [52]. COF-919 exploited its ROS-generating capacity to amplify lipid peroxidation and induce GPX4-mediated ferroptosis. Concurrent pyroptosis/ferroptosis induction remodeled the immunosuppressive TME by boosting T-cell infiltration, thereby suppressing metastasis and recurrence.

#### Inorganic Nanocarriers

Inorganic nanomaterials demonstrate high drug-loading capacity, facile functionalization, and low immunogenicity, positioning them as promising drug delivery candidates [53]. Metal-based carriers, particularly those enabling Fenton/Fenton-like reactions, drive ferroptosis through amplified ROS generation. This section systematically examines inorganic platforms for ferroptosis–immunotherapy, with emphasis on monometallic systems, multimetallic/bimetallic compounds, and nonmetallic counterparts (e.g., carbon/silicon-based platforms).

##### Monometallic Nanomaterials

Metal-based nanomaterials (Au, Mn, Fe, Cu) demonstrate dual therapeutic imaging capabilities in ferroptosis–immunotherapy. Gold nanoparticles, valued for their biocompatibility and nonimmunogenicity, enable drug loading and photoacoustic imaging (PAI) [54]. Du et al. engineered cetyltrimethylammonium bromide/sodium oleate-synthesized gold nanorods functionalized with chitosan and a 12-mer peptide, achieving leukemia suppression through redox imbalance at low doses [55]. Mn-based systems catalyze Fenton-like reactions to amplify ROS/GSH depletion while activating antitumor immunity [56]. A positively charged ultrathin Mn-doped layered double hydroxide (LDH) nanosheet electrostatically coated with IFN-γ enhanced LPO accumulation for ferroptosis induction while stimulating DC-mediated systemic immunity against primary/metastatic tumors [57]. Iron nanomaterials serve dual roles as iron donors and drug carriers. Chin et al. developed chlorophyll (Chl)-Fe(II)-templated Fe_3_O_4_ nanoclusters, where Fe^2+^-Chl π-interactions enabled PDT/chemodynamic therapy (CDT)-mediated ferroptosis to convert immunosuppressive “cold” tumors into immunogenic “hot” phenotypes [58]. In addition, copper-based nanoparticles exhibit unique advantages in ferroptosis–immunotherapy: Cu^2+^ can consume GSH to convert into Cu^+^ and participate in Fenton-like reactions to generate ·OH. Furthermore, the photothermal effect by nanoparticles such as Cu_2−x_S and Cu_2−x_Se can enhance ROS production, thus significantly improving ferroptosis efficiency. Moreover, excessive copper uptake prompts dihydrolipoamide S-acetyltransferase (DLAT) aggregation in tumor cells, triggering cuproptosis that synergizes with ferroptosis to promote ICD effects and enhance antitumor immunity. Building on this, Ruan et al. reported a nanohybrid where Cu_2_O nanoparticles electrostatically bind to *Escherichia coli (E. coli)*'s surface [59]. Following intravenous injection, the nanohybrid accumulates in tumors, where endogenously produced H_2_S converts Cu_2_O into Cu_x_S. Under near-infrared-II (NIR-II) irradiation, this system simultaneously induces ferroptosis via PTT-enhanced Fenton-like reactions and cuproptosis through DLAT aggregation, demonstrating potent antimetastatic efficacy when combined with immune checkpoint blockade. In another study, a Cu_2−x_Se@Fc nanocomposite was formed by electrostatically loading lauric acid (LA) and ferrocene (Fc) onto Cu_2−x_Se nanoparticles [60]. Under NIR irradiation, Cu_2−x_Se NPs produced localized heat through photothermal conversion, melting the phase-change material LA to permit controlled Fc release. The released Fc and Cu^2+^ synergistically triggered Fenton and Fenton-like reactions, inducing destructive lipid peroxidation cascades in tumor cells, which effectively promoted ferroptosis and enhanced antitumor immunity.

##### Metal Nanocomposites

Recent advancements in metal composite nanomaterials have demonstrated therapeutic potential through engineered synergism, enabling multi-mechanistic actions—concurrently triggering ferroptosis via Fenton reactions, stimulating antitumor immunity, and improving treatment outcomes. A notable example is tetrapodal FePd nanocrystals synthesized via co-precipitation of iron and palladium salts under reducing conditions [5]. These nanostructures exhibit glutathione oxidase-like activity, designed to activate autophagy, amplify ferroptosis, and stimulate pro-inflammatory cytokine release in macrophages, thereby enhancing immunotherapeutic efficacy. Another innovative approach involves a CaCO_3_-mineralized nanocomposite featuring a hollow mesoporous Cu_2−x_S core covalently conjugated with glucose oxidase [61]. This system induces mitochondrial dysfunction and glucose deprivation in tumor cells, driving immunogenic ferroptosis. Transition metal-based nanocomposites (e.g., MnMoO_4_, CoMoO_4_, Cu_2_WS_4_) are gaining traction for combined ferroptosis–immunotherapy due to their enzyme mimetic properties. Lei et al. developed PEGylated manganese molybdate nanodots that activate the STING pathway while inducing ferroptosis via Mn^2+^/MoO_4_^2−^-mediated redox reactions, effectively counteracting the immunosuppressive TME [62, 63]. Additionally, PEGylated Cu_2_WS_4_ hollow nanozymes, synthesized via sacrificial templating, exhibited combined radio-sensitizing and immunomodulatory effects through ICD induction [64].

##### Nonmetallic Nanomaterials

Nonmetallic nanomaterials, particularly carbon-based and silicon-based systems, have emerged as promising platforms for synergistic ferroptosis–immunotherapy. Carbon dots (CDs) demonstrate exceptional potential due to their biocompatibility, versatile surface functionalization, and enzyme-mimicking properties. Genistein-derived CDs synthesized through hydrothermal treatment with FA and CuCl_2_ precursors exhibit copper ion coordination via hydrogen bonding, effectively inducing ICD and reprogramming tumor-associated macrophages (TAMs) from immunosuppressive M2 to immunostimulatory M1 phenotypes [65]. Complementary studies reveal that chlorogenic acid-based carbon quantum dots from coffee sources display glutathione peroxidase-like activity, triggering tumor-specific ferroptosis while potentiating antitumor immunity through GSH metabolism disruption [66]. Silicon-based architectures offer distinct advantages including tunable porosity, chemical resilience, and modular surface engineering [67]. Chen et al. developed a multifunctional mesoporous silica nanoplatform integrating upconversion nanoparticles with lauric acid-loaded pores and surface-modified ferroptosis inducers (K_2_FeO_4_)/photosensitizers (psoralen) [68]. NIR activation enables spatiotemporal control of ROS generation for enhanced ferroptosis-PDT synergy and tumor-specific immune activation. Another innovative design employs cation-exchange-derived CaCO_3_/Mn^2+^@SiO_2_ nanocomposites, where Ca^2+^-mediated GSH depletion couples with Mn^2+^-activated STING pathway signaling to orchestrate dual ferroptosis induction and innate immune potentiation [69].

#### Composite Nanocarriers

Metal–organic frameworks (MOFs), constructed through coordination between metal-containing nodes (ions or clusters) and organic linkers, form extended networks with ordered porous architectures [70]. These materials exhibit advantages including high drug-loading capacity, biocompatibility, and precisely tunable pore structures. Notably, the redox-active multivalent metal centers in MOFs enable consumption of TME-abundant species (e.g., H_2_O_2_ and GSH) through valence state transitions while minimizing premature ion leakage during systemic circulation [71]. Moreover, their porous nature enhances specific surface area, facilitating ROS diffusion and amplifying ferroptosis efficacy. These properties position MOFs as promising platforms for combined ferroptosis–immunotherapy. For instance, Fe^3+^-based MIL-53 MOFs with 2-aminoterephthalic acid ligands demonstrated efficient CM-272 loading via electrostatic interactions [71]. This system synergized sonodynamic therapy (SDT) with Fe^3+^-mediated Fenton-like reactions to deplete H_2_O_2_/GSH, amplify ROS toxicity, and induce ferroptosis-coupled ICD, effectively converting immunologically “cold” tumors to “hot” phenotypes. Metal–phenolic networks (MPNs), formed through metal–phenol coordination, offer additional advantages including facile synthesis and structural tunability. The inherent reducibility of polyphenols stabilizes Fe^2+^ and promotes Fe^3+^ reduction, enhancing Fenton reactivity for ROS generation. Xu et al. developed tannic acid (TA)/Fe^3+^ MPNs coated with fibronectin to deliver DOX, achieving synergistic chemotherapy-CDT while activating antitumor immunity via ferroptosis-induced ICD [72]. Another study developed an MPN via Fe^3+^ and gallic acid coordination, electrostatically binding ovalbumin while encapsulating L-buthionine sulfoximine (BSO) through hydrophobic interactions to deplete intracellular GSH, trapping tumor cells in a ferroptosis–immunotherapy cascade to achieve colorectal cancer eradication [73].

Beyond MOFs and MPNs, emerging composite nanocarriers demonstrate distinct advantages in oncotherapy. A recent study developed ultrathin 2D arsenene nanosheets exhibiting exceptional drug-loading capacity through electrostatic adsorption of iridium metal cation complex (IrFN), followed by PDA surface modification [74]. This platform enabled synergistic chemo-immunotherapy via ferroptosis induction, demonstrating enhanced therapeutic efficacy. Separately, Liu et al. engineered an innovative nanoplatform through pyrolysis of FeCo Prussian blue analogues under nitrogen atmosphere to create N-doped graphene-encapsulated FeCo nanocages [75]. Subsequent sulfuric acid etching yielded porous FeCo/Fe-Co bimetallic nanozymes, followed by co-loading of phospholipase A_2_ and LOX, with BSA surface functionalization for improved biocompatibility. The resulting architecture exhibits six-enzyme mimetic activity, simultaneously initiating tumor-selective immunogenic ferroptosis through dual mechanisms: intrinsic multienzyme-driven lipid peroxidation and AA upregulation that synergizes with CD8^+^ T cell-derived IFN-γ to amplify ACSL4-mediated ferroptosis pathways.

#### Biomimetic Nanocarriers

Biomimetic nanoparticles are typically fabricated by coating drug-loaded nanoparticles with cell membranes isolated from specific cell types, preserving core physicochemical properties while inheriting complex biological functions from native membrane components [76]. These protein-enriched nanocarriers demonstrate enhanced biocompatibility, immune evasion, and targeted delivery capabilities [77]. Current research highlights the therapeutic potential of tumor cell membranes, immune cell membranes (M1 macrophages, leukocytes), bacterial OMVs, and blood-derived components (erythrocytes, platelets, hemoglobin (Hb)) in ferroptosis–immunotherapy applications.

Tumor cell membrane-coated nanoparticles exhibit reduced systemic clearance and homologous targeting [78]. Additionally, Wang et al. developed M1 macrophage membrane-coated dendritic mesoporous silica nanoparticles (MSNs) chelated with Fe^3+^-TA complexes [79]. This platform achieved enhanced tumor accumulation through reticuloendothelial system (RES) evasion, simultaneously triggering ICD and facilitating TAM repolarization. Notably, bacterial OMVs demonstrate dual functionality as natural adjuvants and stable drug carriers. Chen et al. engineered OMV-modified hollow MSNs co-loaded with Fe^3+^ and DHA, effectively inducing concurrent ferroptosis in both tumor cells and M2 macrophages while reversing immunosuppression [80]. Erythrocyte membrane-camouflaged nanoparticles show prolonged circulation, exemplified by a red blood cell-coated MOF nanophotosensitizer (Fe^3+^/meso-TCPP) loaded with ascorbic acid [81]. This system reduced tumor recurrence risks through sustained ferroptosis induction and antitumor immunity activation. Platelet membrane-functionalized nanoparticles exploit natural tropism toward tumor vasculature and inflammatory sites. A platelet-camouflaged Fe_3_O_4_ mesoporous nanoparticle loaded with SAS demonstrated enhanced PD-1 blockade efficacy via system Xc^−^ inhibition-mediated ferroptosis, achieving durable tumor regression in metastatic 4T1 models [82]. Hb-based platforms address dual therapeutic needs: oxygen supplementation for PDT and iron supply for ferroptosis. The SRF@Hb-Ce6 nanoassembly exemplifies this strategy, combining oxygen-enhanced PDT with iron-dependent ferroptosis through hydrophobic interaction-mediated SRF loading and covalent Hb-Ce6 conjugation, demonstrating synergistic antitumor efficacy with minimized systemic toxicity [83].

### Key Physicochemical Determinants of Nanocarriers in Ferroptosis–Immunotherapy

Beyond nanomaterial type and composition, key physicochemical properties critically influence drug delivery efficacy and therapeutic outcomes in ferroptosis–immunotherapy. The rational design of nanocarriers for this combined therapeutic approach should strategically optimize the following characteristic parameters:

**(i) Size Optimization (20–100 nm)**

Nanoparticle size governs biodistribution and in vivo delivery efficiency. Optimizing carriers within 20–100 nm balances enhanced permeability and retention (EPR) effect utilization with physiological clearance avoidance [99]. This size range minimizes renal filtration and RES capture while facilitating tumor accumulation. For instance, Bi_2_Fe_4_O_9_ nanosheets (hydrodynamic diameter: 60.05 nm, PDI: 0.108) demonstrate optimal EPR compatibility with low polydispersity, ensuring homogeneous drug distribution and prolonged tumor retention [88].

**(ii) Morphological Engineering**

Nanostructure geometry dictates circulatory persistence, cellular internalization efficiency, and payload capacity. Distinct morphologies confer unique therapeutic advantages: high-aspect-ratio rods prolong circulation and enhance tumor uptake [100]; disk-shaped particles exhibit vascular rolling dynamics through edge-mediated interactions [101]; sharp-tipped needles enable membrane penetration for rapid intracellular delivery [102]. Stimuli-responsive shape transition systems demonstrate enhanced therapeutic precision—for instance, phosphatase-triggered nanofiber formation from endocytosed nanoassemblies induces lysosomal membrane permeabilization, promoting cytosolic drug release [103]. Furthermore, mesoporous architectures (e.g., MOFs, MSNs) combine high-surface-area loading with TME-responsive sustained release. The ordered porosity of MOFs synergistically enhances both drug encapsulation and catalytic ROS generation, potentiating ferroptosis induction.

**(iii) Charge-Tunable Surfaces**

The surface charge of nanocarriers plays a critical role in determining drug delivery efficiency. Surface charge characteristics directly influence cellular membrane interactions: cationic nanocarriers exhibit strong electrostatic binding with anionic domains on tumor cell membranes, significantly enhancing cellular internalization. Conversely, neutral or moderately anionic nanoparticles demonstrate reduced nonspecific protein adsorption and minimized cellular interactions, thereby prolonging systemic circulation through decreased RES clearance while maintaining biocompatibility [104, 105]. Strategic modulation of surface charge enables simultaneous minimization of nonspecific adhesion to healthy cells and optimization of binding to target biomolecules, ultimately improving therapeutic efficacy while reducing systemic toxicity. A representative example involves surface modification with pH-responsive peptide conjugates like 8-polyhistidine modified PEG (PEGH8), which undergo charge reversal from negative to positive in acidic TME [50]. This smart transformation promotes both enhanced tumor cell uptake and facilitated lysosomal escape, significantly boosting delivery efficiency for ferroptosis–immunotherapy agents.

**(iv) Amphiphilic Balance**

Achieving an optimal equilibrium between hydrophilic and hydrophobic properties is crucial for developing stable nanoplatforms in ferroptosis–immunotherapy synergy. Surface functionalization of nanocarriers with polar moieties (e.g., hydroxyl and carboxyl groups) enhances hydrophilicity, thereby creating active interfacial sites that facilitate tumor cell interactions, improve cellular internalization efficiency, and enable spatiotemporally controlled drug release. Alternatively, grafting hydrophilic polymers like PEG through surface modification can minimize opsonization, evade immune surveillance, and extend systemic circulation duration [106]. Conversely, the introduction of nonpolar alkyl chains on nanocarrier surfaces augments hydrophobicity, modulating drug permeability across cellular membranes and release kinetics [107]. Particularly for organic nanocarriers, the precise tuning of hydrophilic–hydrophobic ratios fundamentally governs not only nanomedicine stability and aqueous dispersibility but also determines drug release mechanisms and in vivo biodistribution patterns [108]. Rational engineering of polymer amphiphilicity through balanced incorporation of hydrophilic/hydrophobic segments in macromolecular architectures enables performance optimization, ultimately advancing targeted therapeutic delivery and efficacy in combined ferroptosis–immunotherapy regimens.

**(v) Multimodal Imaging Integration**

Strategic integration of functional components enables engineering of nanocarriers as multifunctional theranostic probes for real-time longitudinal monitoring of therapeutic responses. Incorporating paramagnetic ions (Fe^3+^, Co^2+^, Gd^3+^) enhances T_1_/T_2_-weighted MRI contrast, enabling precise anatomical demarcation and treatment progress evaluation [109]. Concurrent integration of transition metal sulfides or noble metals (Au, Cu, Pd) capitalizes on their strong NIR photon absorption characteristics, thus enabling high-spatial-resolution tumor visualization through PAI [110]. Notably, encapsulation of phase-change materials like perfluorohexane (PFH) permits acoustically triggered microbubble generation under ultrasound irradiation, dramatically amplifying ultrasound imaging (USI) contrast through cavitation effects [111].

**(vi) Optical Responsiveness**

Optoelectronic engineering of nanomaterials significantly enhances therapeutic precision in ferroptosis–immunotherapy integration. Photo-responsive nanoparticles with tailored absorption profiles enable laser-triggered drug liberation and amplified ROS production, potentiating ferroptotic cell death through photodynamic potentiation. Furthermore, strategic incorporation of NIR fluorescent dyes (e.g., IR780, indocyanine green (ICG)) or intrinsically fluorescent therapeutics (e.g., Ce6, DOX) confers dual diagnostic–therapeutic functionality, permitting real-time spatiotemporal tracking of nanocarrier biodistribution and drug payload activation. This multimodal visualization capability facilitates data-driven optimization of treatment parameters and dosage regimens. Through structure–activity optimization of photonic and electronic characteristics, rationally engineered nanoplatforms achieve synchronized therapeutic delivery and response monitoring, ultimately amplifying synergistic therapeutic outcomes in combined ferroptosis–immunotherapy paradigms.

### Nanocarrier Multi-functionality Orchestrating Ferroptosis–Immunotherapy

#### Ferroptosis Induction

**Scheme 2:**
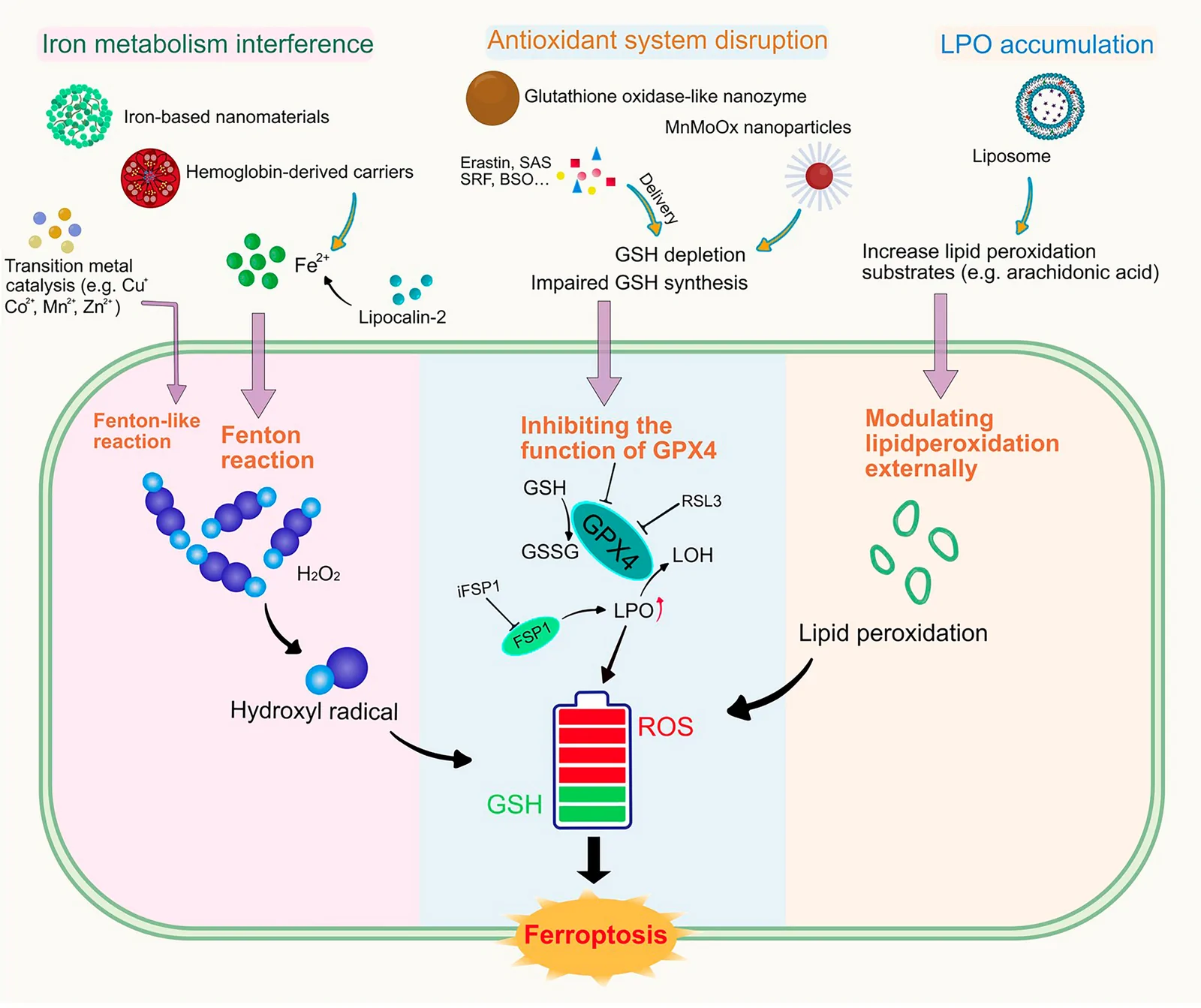
Potential mechanisms underlying nanomaterial-induced ferroptosis: (1) serving as nanocarriers for targeted delivery of ferroptosis inducers; (2) intrinsically triggering ferroptosis through three primary mechanisms: iron metabolism interference, antioxidant system disruption, and LPO accumulation

In tumor ferroptosis–immunotherapy, nanocarriers induce ferroptosis predominantly through targeted delivery of ferroptosis inducers (Scheme 2, Table 3**)**. Erastin, SAS, and SRF suppress cystine uptake via system Xc^−^ inhibition, causing GSH depletion [125]. BSO and arsenic trioxide impair GSH synthesis or directly inactivate GSH, amplifying ferroptotic susceptibility [111, 126]. RSL3 and ML162 alkylate the selenocysteine active site of GPX4, irreversibly inactivating this key antioxidant enzyme [15]. Concurrently, iFSP1 disrupts ferroptosis suppression by targeting ferroptosis suppressor protein 1 (FSP1), a parallel lipid peroxidation inhibitor operating independently of GPX4 [127]. Lipocalin-2 further enhances ferroptosis by altering iron homeostasis through reduced iron uptake/storage and modulation of iron-metabolizing enzymes, elevating intracellular LIP levels [50].

Notably, certain nanocarriers intrinsically induce ferroptosis through three primary mechanisms: iron metabolism interference, antioxidant system disruption, and LPO accumulation (Scheme 2**)**. Iron-based nanomaterials (e.g., Fe-MOFs, iron oxide nanoparticles) and hemoglobin-derived carriers function as iron donors, elevating LIP via controlled Fe^2+^/Fe^3+^ release while inhibiting iron export through ferroportin 1 (FPN1) downregulation. This Fe^2+^ surplus drives Fenton reactions, converting tumor-associated H_2_O_2_ into cytotoxic ·OH for CDT or ferroptosis induction. For instance, Fe-MOFs constructed from 2-aminoterephthalic acid and Fe^2+^ simultaneously serve as drug carriers and Fenton reaction catalysts [90]. Xu et al. demonstrated hemoglobin-based nanocarriers dual functionality: enhancing PDT through oxygen transport while supplementing iron to potentiate ferroptosis [83]. Beyond iron-centric systems, transition metal catalysts (Cu, Co, Mn, Zn) amplify Fenton-like reactions [128]. A copper-based MOF platform exhibited NIR-enhanced Cu^+^-mediated ·OH generation, achieving efficient ferroptosis induction [129]. Nanomaterials also deplete GSH through enzymatic mimicry or covalent conjugation. The Cu_2_WS_4_ nanozyme oxidizes GSH to oxidized glutathione (GSSG) via glutathione oxidase-like activity while suppressing GPX4 through KEAP1/NRF2/HMOX1 pathway modulation [64]. Similarly, MnMoOx nanoparticles leverage high-valence Mo/Mn centers for GSH depletion and GPX4 inhibition [63]. Innovative lipid-like materials containing cinnamaldehyde dimers deplete GSH through Michael addition with thiol groups, creating pro-ferroptotic conditions [130]. Concurrently, lipid-based systems enhance ferroptosis sensitivity through structural design. DAPC liposomes, incorporating AA-derived unsaturated tails, provide substrates for accelerated lipid peroxidation [38]. Liu et al. engineered hybrid liposomes combining lecithin (unsaturated lipids) with DSPE-PEG and γ-Fe_2_O_3_ nanoparticles [131]. The PEG-enhanced membrane permeability facilitates H_2_O_2_/OH diffusion, while embedded Fe^3+^ catalyzes intrabilayer Fenton reactions, directly generating LPO from oxidized unsaturated lipids.

#### Imaging Integration

The integration of imaging modalities into nanoplatforms represents a critical advancement in therapeutic monitoring, enabling real-time visualization of drug delivery trajectories and biodistribution profiles. This capability substantially enhances the optimization of nanomedicine delivery systems while improving therapeutic outcomes [132]. In ferroptosis–immunotherapy synergy, advanced imaging techniques including MRI, fluorescence imaging, and PAI facilitate targeted tumor accumulation of therapeutic agents while minimizing off-target toxicity. Such precision in spatiotemporal drug delivery monitoring ensures improved treatment safety and efficacy [133]. Subsequent sections review current developments in imaging-functionalized nanoplatforms employed in combined ferroptosis–immunotherapy interventions, categorized by their respective imaging methodologies.

##### Magnetic Resonance Imaging

Magnetic resonance imaging (MRI) leverages strong magnetic fields and radiofrequency pulses to excite water proton spins, with spatial encoding via magnetic field gradients enabling detection of relaxometric signals for high-resolution soft tissue visualization [134]. As a cornerstone clinical modality, MRI provides non-invasive visualization at cellular and subcellular resolutions, establishing its utility as a premier anatomical imaging tool [135]. Paramagnetic nanoplatforms containing gadolinium, iron, or manganese—engineered for enhanced magnetization and relaxivity—show particular promise for contrast-enhanced MRI [136]. A representative system employs pH-responsive core–shell nanovesicles containing ascorbic acid (core) and iron oxide nanocubes (shell) [48]. Under magnetic field exposure, vesicle disintegration releases ascorbic acid to reduce Fe^3+^ to Fe^2+^, inducing relaxivity-dependent R2 signal modulation. This dual capability enables MRI-guided monitoring of Fenton reaction dynamics and nanovesicle biodistribution. Furthermore, hyperpolarized ^129^Xe MRI, with its exceptional sensitivity to microenvironmental changes, offers novel detection strategies for evaluating ferroptosis potentiation and immunotherapeutic responses in pulmonary metastases [137].

##### Fluorescence Imaging

Fluorescence imaging (FI) employs wavelength-specific excitation of fluorophores to capture emission signals, offering rapid multi-channel detection, high sensitivity, and non-ionizing operation [138]. When utilizing red-light excitation, this modality enables concurrent PDT through nanoparticle-mediated ROS generation, synergistically enhancing ferroptosis efficacy and immunotherapeutic responses. Smart nanocarriers incorporating activatable fluorescence “switches” improve imaging precision and drug release monitoring. A pH-responsive system using pyropheophorbide A (PPa)-conjugated nanoparticles demonstrates this principle: PPa's π-conjugated structure exhibits aggregation-caused quenching (ACQ) above pH 6.2, while acidic tumor microenvironments (pH < 5.4) restore fluorescence through disaggregation [42]. This pH-dependent signal modulation tracks nanoparticle accumulation and tumor metabolism while enabling image-guided PDT. Such localized treatment induces ICD, stimulates IFN-γ secretion, and subsequently inhibits system Xc^−^ to initiate GPX4-regulated ferroptosis. Notably, NIR imaging capitalizes on reduced tissue scattering and minimal autofluorescence for enhanced penetration depth and spatial resolution [139]. A representative design encapsulates hydrophobic NIR dyes in GSH-responsive nanovesicles, where ACQ maintains an “OFF” state until GSH-triggered disassembly activates fluorescence (“ON” state) [130]. This dual-function platform provides real-time visualization of tumor-targeting and drug release kinetics.

##### Ultrasound Imaging

Ultrasound imaging (USI) employs high-frequency acoustic waves reflected by biological tissues to construct diagnostic images. Transducers emit and receive these waves, with image formation relying on analysis of echo intensity and temporal delay characteristics. While higher frequency waves enable superior spatial resolution, lower frequencies achieve deeper tissue penetration. This modality has gained widespread clinical adoption due to its inherent safety profile (non-ionizing radiation), cost-effectiveness, portability, and real-time imaging capabilities. Perfluorocarbon-based compound PFH, a biocompatible phase-change liquid, demonstrates ultrasound-responsive behavior through acoustic droplet vaporization, thereby enhancing echogenic signals. A recent investigation developed PFH-encapsulated lipid nanoparticles as advanced ultrasound contrast agents [111]. These agents significantly improved imaging contrast, delineated tumor margins with enhanced clarity, and facilitated therapeutic monitoring. In hepatocellular carcinoma models, the nanomedicine demonstrated dual functionality: effective induction of tumor cell ferroptosis through iron-dependent lipid peroxidation and synergistic antitumor activity when combined with PD-L1 immune checkpoint inhibitors.

##### Photoacoustic Imaging

Photoacoustic imaging (PAI) merges the high spatial resolution of optical imaging with ultrasound's deep tissue penetration by detecting laser-induced ultrasonic waves. This modality operates through wavelength-selective light absorption in biological tissues, generating localized thermoelastic expansion that emits detectable ultrasound signals for image reconstruction [110]. Transition metal sulfides, molybdenum oxides (MoO_x_), and palladium-based nanomaterials constitute principal PAI contrast agents, potentiating imaging quality through localized modulation of optoacoustic properties [5]. Iron–palladium (FePd) nanocrystals exemplify multifunctional agents with superior photothermal conversion efficiency, enabling real-time therapy guidance while synergistically inducing tumor ablation through ferroptosis promotion and immunomodulation. Manganese molybdate (MnMoO_x_) nanoparticles demonstrate microenvironment-responsive performance, developing tunable NIR absorption peaks upon GSH interaction [63]. Quantifying tumor-associated photoacoustic signal variations have established optimal administration schedules for precision theranostics.

##### Multimodal Imaging

Multimodal imaging synergistically integrates complementary imaging modalities to overcome inherent limitations of single techniques, advancing tumor diagnosis and treatment strategies [140]. A theranostic nanoplatform exemplifies this approach through its integration of ultra-small manganese ferrite (UMF) nanoparticles for dual-modality MRI/PAI and PFH as a phase-change ultrasound contrast agent [114]. NIR laser irradiation enables simultaneous PTT with real-time temperature monitoring via infrared thermography, achieving precise thermal ablation of triple-negative breast tumors while enabling controlled drug release. Parallel developments in phototheranostic metal–phenolic networks (PF-MPNs) demonstrate enhanced NIR-II fluorescence/photoacoustic tracking capabilities through Fe^3+^ coordination with semiconducting polyphenolic-PEG conjugates [141]. Tumor-specific accumulation is evidenced by time-dependent fluorescence/PA signal enhancement at target sites, contrasted with hepatic/splenic clearance patterns reflecting systemic pharmacokinetics. The semiconductor polymer backbone further ensures superior photothermal conversion efficiency, enabling quantitative heat imaging for real-time PTT efficacy assessment. These multimodal platforms collectively enable early tumor detection, image-guided therapy, and treatment response monitoring through integrated diagnostic-therapeutic functionality.

#### Targeting Capability

Passive nanoparticle targeting predominantly relies on the EPR effect, enabling selective accumulation in tumor tissues through leaky vasculature [142]. Surface-engineered nanoplatforms achieve active targeting through four distinct mechanisms: tumor cell-specific binding, subcellular localization, TME engagement, and homotypic recognition. These functionalization strategies synergistically enhance therapeutic precision by directing drug internalization pathways. The following sections critically evaluate these active targeting approaches within the context of ferroptosis-mediated immunotherapy.

##### Tumor Cell-Specific Binding

Surface modification of nanocarriers with small molecular ligands like butyrate, phenylboronic acid (PBA), and folate (FA) enhances tumor-targeting capabilities in ferroptosis–immunotherapy. Butyrate demonstrates particular promise through its interaction with monocarboxylate transporter 1 (MCT-1), which shows differential intestinal expression and overexpression in hepatocellular carcinoma. Butyrate-conjugated PLGA-lipid nanoparticles leverage this mechanism, enhancing intestinal drug penetration and enabling sustained delivery to hepatic tumors [85]. FA-modified nanoplatforms capitalize on folate receptor upregulation in malignancies, exemplified by MOF-based systems that improve tumor cell uptake while minimizing systemic toxicity [33]. Similarly, 4-carboxyphenylboronic acid targets glycoprotein receptors overexpressed in breast and other cancers, significantly enhancing therapeutic precision for ferroptosis-mediated immunotherapy [58]. These ligand-directed strategies collectively improve tumor specificity while reducing off-target effects.

Beyond small molecules, peptides and proteins offer enhanced tumor-targeting capabilities for nanocarriers. The tumor-penetrating iRGD peptide selectively binds αvβ3 integrins and neuropilin-1 in solid tumors, improving targeting efficiency and tissue penetration when co-delivered with nanotherapeutics [143]. Fibronectin's intrinsic RGD motif similarly targets integrin αvβ3-rich tumor microenvironments [72]. Kong et al. demonstrated synergistic effects by combining the angiogenesis-targeting APRPG peptide (specific to tumor-associated αvβ3) with cell-penetrating octa-arginine (R8), significantly boosting tumor-selective drug uptake for ferroptosis–immunotherapy [39]. Emerging ligands like the VGB3 peptide enable dual targeting of vascular endothelial growth factors receptor (VEGFR)-1/VEGFR-2 in 4T1 tumors, enhancing both therapeutic efficacy and imaging potential [114]. Ferritin-based nanoparticles exploit transferrin receptor 1 (TfR1) overexpression in malignancies, effectively inducing ferritinophagy-mediated ferroptosis [113]. These biomolecular targeting strategies address tumor heterogeneity while optimizing therapeutic precision.

Carbohydrate-based ligands including hyaluronic acid (HA) and fucose demonstrate potent tumor-targeting potential. HA-functionalized nanocarriers exploit CD44 overexpression in malignancies, exemplified by HA-grafted HGF nanoparticles selectively targeting melanoma cells in ferroptosis–immunotherapy [144]. Fucose-modified systems leverage upregulated C-type mannose receptor 1 in tumor and immune cells, enhancing both nanocarrier internalization and CpG ODNs' immunostimulatory efficacy [38]. These sugar-mediated targeting strategies synergize recognition of tumor-specific receptors with therapeutic payload delivery optimization.

##### Mitochondrial Localization

Mitochondria, crucial regulators of redox homeostasis, paradoxically drive ferroptosis via oxidative stress and excessive ROS generation when functionally impaired [145]. This dual role positions them as strategic subcellular targets for ferroptosis-mediated cancer immunotherapy. Triphenylphosphine (TPP), a prototypical mitochondrial-targeting moiety, demonstrates effective incorporation into liposomal phospholipid bilayers, enabling mitochondrial-specific drug delivery. Mechanistically, TPP's proton sponge effect in lysosomes induces osmotic swelling and membrane rupture, facilitating liposomal escape and subsequent mitochondrial accumulation of therapeutic payloads [112]. Parallel mitochondrial targeting is achieved by iridium complexes, which synergistically disrupt cellular antioxidant defenses through ROS amplification, thereby potentiating ferroptosis induction [74].

##### TME Engagement

The TME represents a complex ecosystem comprising malignant cells, stromal immune/inflammatory cells, cancer-associated fibroblasts (CAFs), and TAMs, critically orchestrating tumorigenesis and progression [146]. CAFs drive metastasis, angiogenesis, and therapeutic resistance through ECM remodeling and growth factor secretion [147], establishing their elimination as a crucial therapeutic strategy. Capitalizing on Angiotensin II type I receptor (AT1R) overexpression in both CAFs and breast cancer cells, researchers developed telmisartan-functionalized Fe/Co bimetallic nanocarriers leveraging the drug's “Delta lock” structural affinity. These nanovehicles demonstrated dual targeting capabilities, effectively disrupting cancer cells, degrading ECM barriers, and enhancing drug penetration [121]. Concurrently, TAMs emerge as dominant immunosuppressive components within TME, promoting tumor progression through multiple pathways. Targeting CD206-overexpressing M2-polarized macrophages, Tang et al. engineered mannose-conjugated MSNs to induce ferroptosis via erastin loading while reversing TAM-mediated immunosuppression [148]. Hypoxia-targeted delivery systems have been developed through microbial–nanomaterial hybridization. Engineered *E. coli*-based nanohybrids demonstrated preferential accumulation in hypoxic tumor regions, facilitating site-specific drug delivery through anaerobic bacterial tropism [59]. These multimodal strategies collectively address TME complexity through stromal reprogramming, immune modulation, and microenvironment-specific targeting.

##### Homotypic Recognition

Cell membrane-coated nanoparticles demonstrate remarkable potential in ferroptosis–immunotherapy through their inherent biocompatibility, immune evasion properties, and active targeting capabilities. Platelet membrane-encapsulated Fe_3_O_4_ nanoparticles exhibit enhanced tumor-targeting specificity compared to uncoated counterparts, particularly in lung metastasis models, where they achieve superior accumulation and ferroptosis inducer delivery efficiency [82]. This targeted approach significantly improves therapeutic outcomes in metastatic tumors through amplified ICD mechanisms. Leukocyte membrane-functionalized PLGA nanoparticles leverage surface protein interactions to prolong systemic circulation while reducing off-target toxicity [149]. This biomimetic design enhances tumor-specific delivery in both acute myeloid leukemia (AML) and colorectal cancer models, demonstrating improved therapeutic indices for ferroptosis-mediated immunotherapy. Similarly, macrophage membrane-coated nanoparticles show differential targeting behavior depending on polarization state—M1 macrophage membrane-cloaked BSA nanoparticles demonstrate enhanced tumor tropism and mononuclear phagocyte system evasion, significantly improving pharmacokinetic profiles and therapeutic efficacy [150]. Tumor cell membrane-coated nanoparticles exploit homologous recognition mechanisms through preserved “self-marker” surface proteins. Gastric cancer cell membrane-encapsulated MSNs exemplify this strategy, combining immune evasion with intrinsic tumor homing capabilities to enhance local retention and ferroptosis-mediated immunogenicity [96]. Advanced hybrid systems integrating 4T1 tumor membrane-coated Fe_3_O_4_ nanoparticles with bacterial-derived vault nanoparticles (VNPs) demonstrate synergistic targeting through both VNP tumor tropism and membrane-mediated homing [124]. This dual-targeting approach amplifies intracellular H_2_O_2_ accumulation while inducing M1 macrophage polarization, effectively bridging ferroptotic cell death with adaptive immune activation.

#### Stimulus Responsiveness

In cancer therapeutics, stimulus-responsive nanocarriers have emerged as a critical strategy for enabling precise spatiotemporal control of therapeutic agents by responding to TME characteristics or external stimuli. This paradigm addresses two fundamental challenges: preventing premature drug leakage during systemic circulation while enhancing tumor-specific payload release. The subsequent discussion focuses on key stimulus-responsive mechanisms applicable to ferroptosis–immunotherapy synergy, categorized as endogenous triggers, exogenous stimuli, and multi-stimuli-responsive systems.

##### Endogenous Stimuli

While normal tissues maintain a physiological pH of ~ 7.4, the TME exhibits an acidic ECM (pH 6.5–7.4). Exploiting the acidic characteristics of TME, a wide variety of pH-responsive nanocarriers have been developed [151]. Capitalizing on this pH differential, pH-responsive nanosystems have been engineered through non-covalent assembly of anti-PD-L1 peptides, spermidine-functionalized dextran, and Fe^3+^-TPP coordination complexes [94]. Under acidic TME conditions, spermidine amino group protonation disrupts Fe^3+^ coordination via enhanced electrostatic repulsion, enabling tumor-specific iron release to potentiate ferroptosis. Exploiting the redox imbalance in cancer cells, redox-responsive nanocarriers are engineered to enable precise drug release by sensing heightened intracellular GSH/ROS levels within tumor cells [152]. GSH-responsive platforms have garnered significant attention given the four–tenfold higher GSH concentrations in tumors versus normal tissues. A representative system employs disulfide-bridged organosilica hybrid micelle that undergo tumor-selective disulfide cleavage, triggering co-release of SAS and DOX to synergistically induce ferroptosis and pyroptosis while activating antitumor immunity [44]. ROS-overproducing tumors have motivated the development of oxidative stress-responsive carriers. An engineered nanoplatform combining TCPP with ROS-cleavable thioketal (TK) linkers and PAMAM dendrimers demonstrates tumor-selective disintegration [49]. ROS-mediated TK cleavage liberates hafnium ions and HIF-1α-targeting siRNA, synergistically enhancing ferroptosis and overcoming hypoxia-mediated therapy resistance. In addition, enzyme-responsive systems exploit tumor-overexpressed proteases for precision therapy. A hemoglobin-based nanoparticle incorporating MMP-2-cleavable peptide linkers ensures tumor-specific activation, releasing the ferroptosis inducer SRF exclusively in MMP-2-high malignancies while sparing healthy tissues [83].

##### Exogenous Stimuli

External physical stimuli including ultrasound, light, and magnetic fields enable spatiotemporally controlled drug activation in ferroptosis–immunotherapy nanoplatforms. Ultrasound demonstrates particular promise due to its non-invasive nature and adjustable tissue penetration. Perfluorooctyl bromide-modified PLGA nanoparticles achieve high-intensity focused ultrasound (HIFU)-triggered gambogic acid release, effectively initiating tumor ferroptosis [84]. Similarly, light-responsive platforms exploit wavelength/power-tunable energy deposition. Cheng et al. developed laser-activatable PLGA nanoparticles encapsulating PFH, where photo-triggered phase transition drives rapid payload release, achieving tumor-specific drug accumulation and cytotoxicity [114]. In contrast to energy-dependent modalities, magnetic field-responsive systems exploit mechanical forces for precision therapy. Core–shell vesicles containing iron oxide nanocubes (IONCs) undergo structural deformation under rotating magnetic fields, releasing encapsulated ascorbic acid [48]. This initiates IONC-mediated Fe^2+^ liberation via Fenton reactions, simultaneously driving ferroptosis and ICD while bypassing biological barriers through physical field penetration.

##### Multi-stimuli Responsiveness

Multi-stimuli-responsive systems have emerged to achieve tumor-selective multistage drug delivery with enhanced therapeutic precision. A pH/light-dual responsive nanoplatform demonstrates acid/light-triggered degradation, enabling sequential activation of ferroptosis inducers and ICD triggers [50]. Moreover, pH/ROS-responsive architectures particularly exploit tumor-specific acidosis and oxidative stress. A PDBA-based diblock copolymer grafted with DHA and PBA undergoes pH-dependent protonation and H_2_O_2_-responsive ester cleavage, achieving spatiotemporal delivery of RSL3/DHA payloads [115]. Notably, quadruple-responsive systems integrate pH/ROS/GSH/US sensitivity through rational material engineering. Mesoporous MnOx nanoparticles coated with silk fibroin leverage β-sheet dissociation (pH/ROS-sensitive) and disulfide cleavage (GSH-responsive) for tumor-selective ICG derivative release [116, 153]. Meanwhile, ultrasound irradiation further amplifies payload liberation through cavitation effects, creating a synergistic therapeutic cascade that overcomes TME heterogeneity.

### Artificial Intelligence-Enabled Smart Nanoplatform Design

Although current nanotechnology platforms exhibit significant potential in ferroptosis–immunotherapy, traditional nanomaterial design primarily relies on empirical screening and trial-and-error optimization, which is limited by long development cycles and high costs. Additionally, their clinical translation is confronted with complex variables and optimization challenges, including restricted drug delivery efficiency, substantial inter-individual variability, and unpredictable treatment responses [154]. Recent years have witnessed breakthroughs in artificial intelligence (AI) technology offering novel solutions to these challenges. AI has shown tremendous potential for aiding nanomaterial screening, predicting in vivo processes, and assessing nanoparticle efficacy. AI-powered intelligent nanodelivery systems are anticipated to facilitate major advancements in ferroptosis–immunotherapy. First, by integrating multi-omics data with nanosensor technologies, AI enables precise tumor biomarker analysis and facilitates personalized drug screening via computational simulations, thus optimizing treatment regimens [155]. Second, leveraging machine learning algorithms and computational models, AI can systematically optimize the design and properties of nanoparticles. This optimization targets parameters such as their size, surface charge, drug encapsulation efficiency, interactions with target drugs, vasculature, immune system, and cell membranes, as well as drug release kinetics [156]. Such optimization accelerates novel nanoparticle formulation discovery and enhances therapeutic outcomes. Notably, a recent study leveraged AI-powered image segmentation to analyze the permeability of over 67,000 individual blood vessels across 32 tumor models, employing drug- and tracer-loaded ferritin nanocages. This high-throughput approach revealed distinct nanoparticle transport mechanisms: passive extravasation via the EPR effect in highly permeable tumors, and active trans-endothelial transport in poorly permeable ones. By refining ferritin nanocage design (e.g., incorporating pH-responsive peptides and albumin-binding domains), researchers significantly enhanced drug delivery efficiency in low-permeability tumors [157]. Finally, AI-enabled nanoparticle systems integrate sensing modules and imaging agents to enable a dynamic monitoring-feedback-optimization loop in tumor treatment response. Real-time data analysis allows AI algorithms to precisely evaluate efficacy and adaptively adjust therapeutic strategies, markedly improving treatment precision and clinical response rates [158].

## Synergistic Nano-enabled Mechanisms Coordinating Ferroptosis–Immunotherapy Crosstalk

The ferroptosis–immunotherapy combination demonstrates synergistic efficacy in tumor therapy, eliminating primary lesions while activating systemic immunity to inhibit metastasis and recurrence. This section analyzes key strategies to augment antitumor immunity through: (1) Direct immune potentiation, (2) Immunosuppressive TME reversal, and (3) Critical TME physical-property remodeling. Table 4 summarizes the discussed mechanisms.

**Table 4:** Synergistic strategies: Nanoplatform-engineered ferroptosis–immunotherapy amplification

Strategy		Nano-formulation	Synergistic agent	Synergistic ferroptosis–immunotherapy mechanistic pathway	References
Direct amplification of antitumor immunity	Augmenting immunogenic cell death	Tumor cell membrane-coated MOF	GOx/DOX/Fe^2+^	Ferroptosis synergizes with DOX chemotherapy to potentiate ICD, triggering amplified antitumor immunity	[159]
		PEGylated Cu_2_WS_4_ nanoparticle	Cu^+^/W^6+^	Cu_2_WS_4_-based radioferrotherapy induces ICD, releasing DAMPs to activate DCs and drive T cell infiltration, amplifying antitumor immunity	[64]
		Semiconducting iron-chelating nano-immunomodulator	SPC/Fe^3+^	Fenton reaction driven by Fe^3+^ → Fe^2+^ redox cycling generates ROS, with ultrasound-activated SPC augmenting oxidative stress to induce ferroptosis-triggered ICD, eliciting DAMPs release and DC maturation	[43]
		Up-conversion SiO_2_ nanoparticle	Psoralen/K_2_FeO_4_	K_2_FeO_4_/psoralen-PDT orchestrates O_2_ supply, GSH depletion, and ROS overproduction, synergistically inducing ferroptosis-driven DC maturation and antitumor immunity	[68]
		Carrier-free nanoassembly	PPa/Erastin	PPa-mediated PDT generates substantial ROS that, combined with Erastin, synergistically triggers ferroptosis, thereby enhancing antitumor immune responses	[160]
		Metal–phenolic network	IR820/CO	CO gas therapy synergizes with PTT/ferroptosis induction in triple-combination therapy, driving ICD amplification for potent tumor immunotherapy	[161]
		*E. coli*-coated nanoparticle	Cu_2_O	Cu_2_O-mediated PTT potentiates ferroptosis/cuproptosis to reprogram immunosuppressive TME into immunoresponsive state via DCs maturation and T cell priming	[59]
		Cancer cell membrane-coated nanoparticle	Pt(II)	Pt^2+^ activates NOXs to generate H_2_O_2_, thereby potentiating ferroptosis and elevating ICD	[96]
		FeAl-LDH nanoparticle	DHA	Fe^2+^-triggered DHA degradation amplifies ROS production, enhancing ferroptosis-driven DAMPs release and subsequent antitumor immunity	[162]
		Cu/Zn bimetallic MOF	siNFS1	siNFS1 cooperates with Cu^2+^ to elevate LIP, augmenting ferroptosis and ICD	[163]
		Poly(amidoamine) nanoparticle	Ce6	Ce6-based PDT induces ROS-mediated lipid peroxidation while suppressing AKT-mTOR/GPX4 axis to impair LPO detoxification, synergistically augmenting ferroptosis-driven ICD	[127]
		Coordination polymer nanofiber	Gallic acid/Fe^2+^	PTT-mediated hyperthermia augments Fenton reaction and depletes GSH, synergistically amplifying ferroptosis and ICD induction	[93]
		PLGA nanoparticle	Salinomycin	Salinomycin upregulates IRP2-mediated TfR expression and suppresses Ferritin, amplifying iron-driven ferroptosis and ICD	[85]
		Tetrapod FePd nanocrystal	–	The tetrapodal needle-like architecture induces ferritin-selective autophagy, potentiating tumor ferroptosis and amplifying ICD	[5]
	Adjuvant-driven innate immune priming	Liposome	CpG ODN	CpG ODN activates TLR9 to drive DC maturation and MHC-II upregulation, potentiating Th1 polarization and pro-inflammatory cytokine production that activate both innate and adaptive immunity	[38]
		FeAl-LDH nanoparticle	Al	Al^3+^/ROS-induced oxidative stress enables in situ nanovaccine self-assembly, eliciting systemic immunity that inhibits distant tumor progression and pulmonary metastasis	[162]
	cGAS-STING signaling activation	Mn-based nanoparticle	Mn^2+^	Mn^2+^ potentiates cGAS sensing of dsDNA and amplifies STING activation in DCs	[164]
		MnMoO_4_ nanodot	Mn^2+^/MoO_4_^2−^	Mn^2+^/MoO_4_^2−^ co-activate cGAS-STING signaling via STING → TBK1 → IRF3 phosphorylation cascade, promoting DC maturation and IFN-β production	[62]
		Cu/Zn bimetallic MOF	Zn^2+^	Zn^2+^ potentiates cGAS-STING activation, triggering inflammatory cytokine/DAMPs release to amplify DC maturation and induce robust ICD, thereby activating antitumor immunity	[163]
		FeGd nanoparticle	SN38	SN38 triggers STING signaling to induce IFN-β secretion via DNA exosomes, activating NK/CD8^+^ T cells and potentiating antitumor immunity	[165]
		Liposome	ML385	ML385 triggers STING signaling in tumor MDSCs, alleviating CTL suppression and reprogramming immunosuppressive TME toward immunostimulatory phenotypes to enhance antitumor responses	[37]
Disruption of immunosuppressive tumor niches	Coordination with ICB-based therapy	Bi_2_Fe_4_O_9_ nanoparticle	Anti-PD-1 antibody	Anti-PD-1 blockade reverses immunosuppressive TME by disrupting PD-1/PD-L1 axis, reprogramming antitumor immunity	[88]
		MnMoO_x_ nanoparticle	Anti-PD-L1 antibody	PD-L1 blockade unleashes pre-existing antitumor T cell immunity via checkpoint inhibition	[63]
		Metal–phenolic network	DNAzyme	DNAzyme-driven PD-L1 silencing synergizes with ferroptosis–immunotherapy to eradicate primary/abscopal tumors	[91]
		Fe_2_O_3_ nanoparticle	^D^PPA-1	^D^PPA-1-mediated PD-L1 blockade augments tumor-infiltrating CD8^+^ T lymphocytes	[118]
		Metal–phenolic network	Anti-CTLA-4 antibody	Anti-CTLA-4 blockade augments cytotoxic T lymphocyte proliferation	[161]
		Polymeric nanoparticle	IND	IND-mediated IDO inhibition suppresses Treg activity while eliciting antitumor immunity	[166]
		M1 macrophage membrane-coated nanoparticle	PEP20	PEP20 potentiates macrophage-mediated tumor phagocytosis via CD47-SIRPα blockade, synergizing ferroptosis–immunotherapy	[150]
		Polymeric nanoparticle	Anti-B7-H3 × CD3 biAbs	BiAbs block tumor B7-H3 and engage T cell CD3, amplifying T cell activation and IFN-γ secretion	[51]
	Precision modulation of immunosuppressive cell populations	Semiconducting iron-chelating nano-immunomodulator	Fe^3+^	Fe^3+^ depletes GSH and synergizes with SPC sonodynamic therapy, polarizing M1-TAMs while reprogramming M2-TAMs, coupled with DC activation to amplify antitumor immunity	[43]
		OMV-coated nanoparticle	Fe^2+^	Fe^2+^ depletes intratumoral GSH through Fe^2+^-driven Fenton/DHA reactions, generating radical flux to trigger ferroptosis in neoplastic cells and M2-TAMs	[80]
		Liposome	Liproxstatin-1	Liproxstatin-1 alleviates immunosuppressive TME by blocking neutrophil ferroptosis	[167]
Biophysical hallmark remodeling in TME	Modulating intratumoral hypoxia	CaCO_3_/Mn^2+^@SiO_2_ nanocomposite	Ca^2+^/Mn^2+^	CMS mitigate tumor hypoxia through H_2_O_2_-catalyzed O_2_ generation, downregulating HIF-1α to activate innate immunity and drive M2-to-M1 TAM repolarization	[69]
		Cu_2−x_Se/ZIF-8 heterostructure	Cu^+^	Cu^+^-catalyzed H_2_O_2_ decomposition generates O_2_ to remodel hypoxic TME, reprogramming TAMs and activating CD8^+^ T cells	[33]
	Precision modulation of the extracellular matrix	Fe/Co bimetallic MOF	Telmisartan	Telmisartan selectively targets CAFs to trigger ferroptosis, driving collagen degradation and ECM remodeling	[121]

### Direct Amplification of Antitumor Immunity

#### Augmenting Immunogenic Cell Death

Ferroptosis eliminates tumor cells while eliciting antitumor immunity across solid malignancies. This process triggers ICD, marked by the release of DAMPs from dying cells. These signals recruit antigen-presenting cells (APCs) and activate T-cell responses through immune cell coordination [17]. However, ICD efficacy remains constrained by tumor immune evasion mechanisms and immunosuppressive microenvironments [168]. Strategic combination with chemotherapy, radiotherapy, phototherapy, or SDT can synergize with ferroptosis-based therapy to amplify ICD. Multimodal ferroptosis enhancement strategies thus potentiate immunogenic signaling, reinforcing antitumor immunity.

##### Therapeutic Synergy Amplifying ICD Signal

**Fig. 1:**
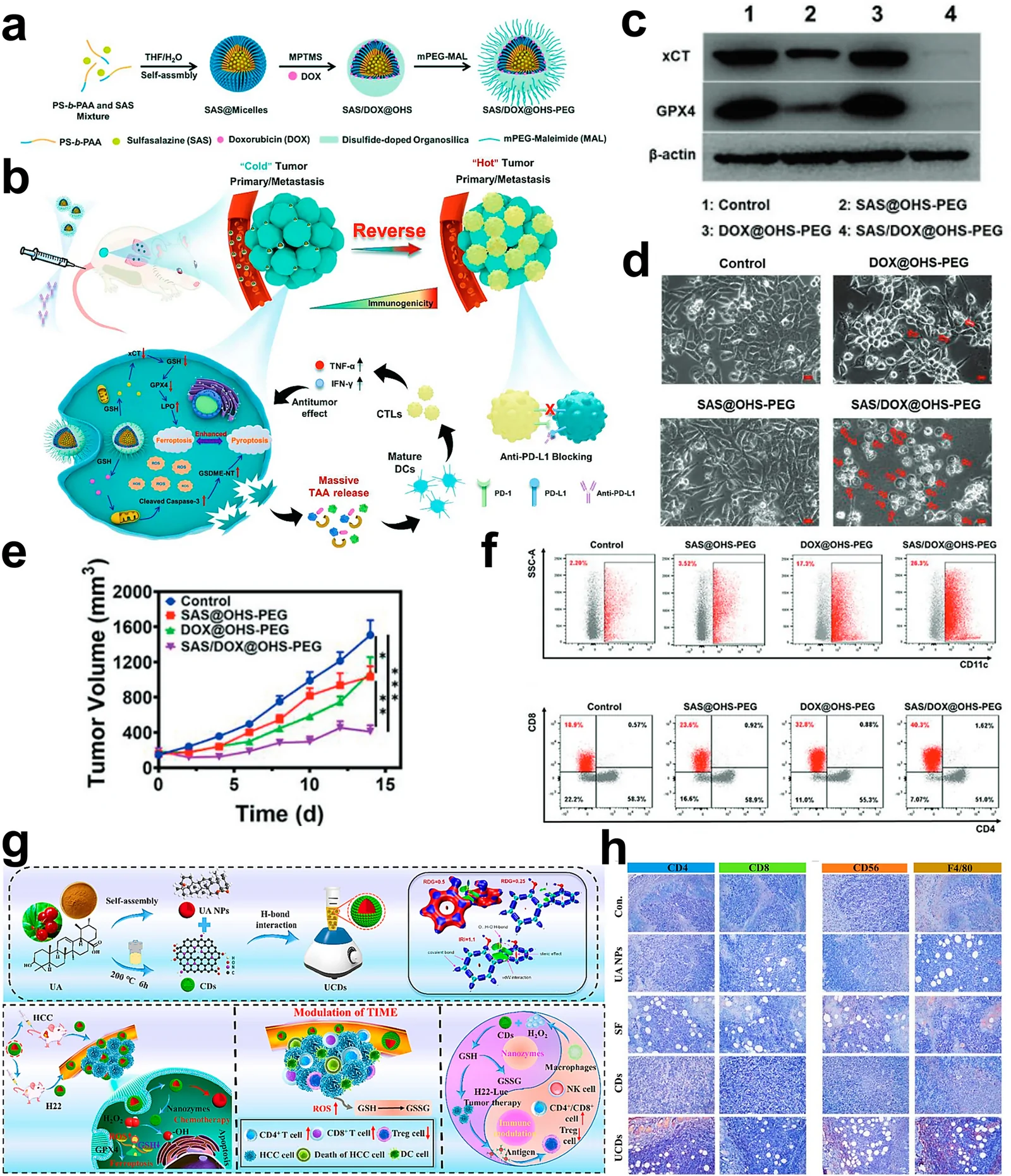
**a** Synthesis process of SAS/DOX@OHS-PEG nanomodulator. **b** Schematic illustration of SAS/DOX@OHS-PEG nanomodulator for enhancing “cold” tumor immunotherapy and suppressing pulmonary metastasis through ferroptosis-pyroptosis induction. **c** Western blot analysis of xCT and GPX4 expression in 4T1 cells post-treatment. **d** Morphological changes in 4T1 cells after treatments (scale bar: 50 μm), showing characteristic cell swelling with large bubbles (red arrow). **e** Tumor growth kinetics in different treatment groups. **f** Flow cytometry analysis of CD11c^+^ DC infiltration in tumors and CD4^+^/CD8^+^ T cell populations in splenic CD3^+^ T cells. Adapted with permission from [44], copyright 2023 Wiley–VCH GmbH, Weinheim. **g** Design and functional characteristics of UCDs enabling enhanced tumor accumulation, ferroptosis induction, and combination therapy. **h** Immunohistochemical analysis of CD4^+^, CD8^+^, CD56^+^, and F4/80^+^ cells in H22 tumors. Adapted with permission from [95], copyright 2024 American Chemical Society

Current chemotherapy employs cytotoxic agents targeting proliferative processes through DNA fragmentation, cytoskeletal inhibition, and ATP synthesis blockade [169]. Notably, anthracyclines and DNA-damaging agents are known to trigger ICD, characterized by CRT exposure and HMGB1 release, which promote DC maturation and subsequent T cell activation [170]. Building on this concept, one previous study engineered SAS/DOX@OHS-PEG, a nanomodulator co-delivering ferroptosis inducer SAS and DOX (Fig. 1a) [44]. This dual-action system synergistically induces ferroptosis and pyroptosis, enhancing TAAs release to stimulate DC maturation and CTL infiltration, thereby reprogramming immunosuppressive TME in cold tumors (Fig. 1b). Western blot analysis confirmed SAS-mediated xCT inhibition and GPX4 downregulation, significantly enhancing LPO accumulation—an effect potentiated by DOX co-administration (Fig. 1c). Concurrently, DOX activated gasdermin E (GSDME) cleavage via caspase-3, inducing characteristic pyroptotic morphology (cellular swelling and membrane blebbing) in 4T1 cells within 24 h (Fig. 1d). The nanomodulator demonstrated superior therapeutic efficacy (72.8% tumor inhibition rate) compared to monotherapies, indicating synergistic tumor elimination (“1 + 1 > 2” effect) (Fig. 1e). Immune profiling revealed enhanced DC recruitment (2.2%–26.3%) and subsequent CTL infiltration, confirming enhanced ICD through ferroptosis-pyroptosis synergy (Fig. 1f). Parallel development of ursolic acid nanoparticle (UA NPs)-carbon dot conjugates (UCDs) achieved similar TME remodeling through combined chemotherapy and ferroptosis, converting immunologically cold tumors to hot phenotypes via T cell/NK cell/macrophage infiltration (Fig. 1g, h) [95].

**Fig. 2:**
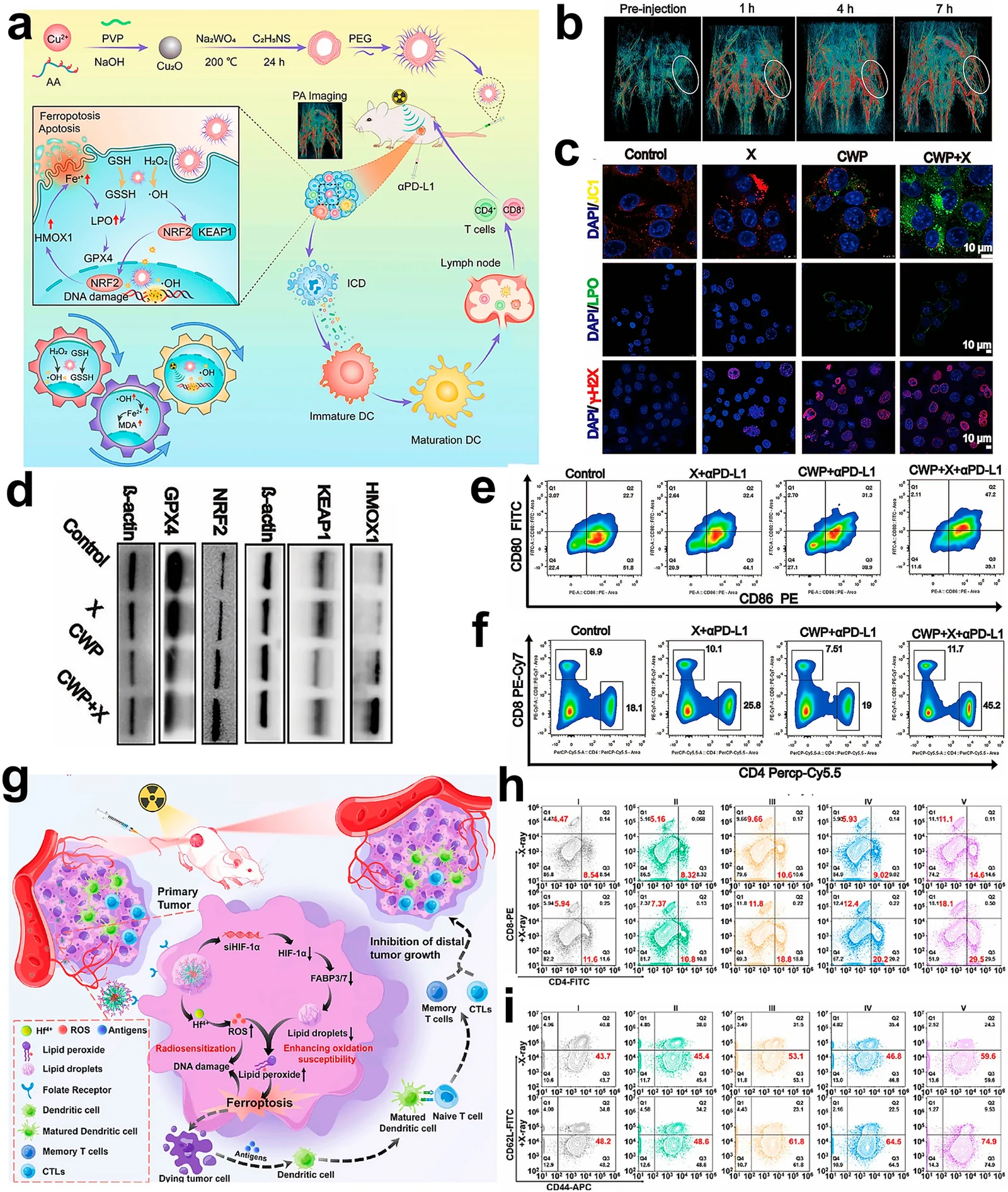
**a** Synthesis of CWP and its mechanism: Multifunctional CWP-mediated sensitization enhances breast cancer therapy through PA imaging-guided radio-immunotherapy. **b** In vivo photoacoustic imaging of tumor-bearing nude mice post-CWP injection (20 mg/kg). **c** Fluorescence imaging of 4T1 cells treated with CWP ± X-ray: Mitochondrial damage (JC-1), lipid peroxidation (LPO), and DNA damage (γ-H2AX) visualized by DAPI co-staining. **d** Western blot analysis of KEAP1, NRF2, HMOX1, and GPX4 expression in 4T1 cells under various treatments. **e** Flow cytometry assessment of DC maturation (CD80^+^/CD86^+^) in lymph nodes. **f** Flow cytometry analysis of splenic T cell subtypes: CTLs (CD3^+^CD4^−^CD8^+^) and helper T cells (CD3^+^CD4^+^CD8^−^). Adapted with permission from [64], copyright 2024 Wiley–VCH GmbH, Weinheim. **g** Design of TCPP@Hf-TK-PEG-PAMAM-FA@siHIF-1α nanoassemblies and their low-dose radiation-activated ferroptosis–immunotherapy mechanism. Quantitative flow cytometry analysis of **h** CD4^+^/CD8^+^ T cell infiltration and **i** CD8a^+^CD44^+^CD62L^−^ effector memory T cells in distal tumors across treatment groups. Adapted with permission from [49], copyright 2023 American Chemical Society

Radiotherapy exerts localized cytotoxic effects through ionizing radiation-induced biomolecular damage and elevated oxidative stress [171]. Emerging evidence suggests its capacity to induce ICD, potentially reprogramming immunosuppressive TME for systemic antitumor immunity or immunotherapy synergy [172]. Integrating ferroptosis induction with radiotherapy in nanotherapeutic systems amplifies ICD-mediated immune activation. The PEG-coated Cu_2_WS_4_ nanozyme (CWP) exemplifies this approach through dual mechanisms: Cu^2+^-mediated Fenton-like reactions and tungsten (W)-enhanced radiosensitization via high-Z element energy deposition (Fig. 2a) [64]. CWP demonstrated tumor-targeting capability through PAI, showing maximal intratumoral accumulation 7 h post-injection under 808 nm excitation (Fig. 2b). In vitro, CWP + X-ray treatment induced pronounced nuclear DNA damage in 4T1 cells, confirmed by intensified γ-H2AX foci (Fig. 2c). Western blot analysis revealed GPX4 suppression, verifying ferroptosis induction (Fig. 2d). Immune profiling showed CWP + X-ray + aPD-L1 treatment enhanced mature DCs (CD80^+^CD86^+^) by 24.5% and tumor-infiltrating CD8^+^ T cells by 4.8% versus controls (Fig. 2e, f), confirming enhanced antitumor immunity through ICD potentiation. Similarly, the TCPP-TK-PEG-PAMAM-FA nanocomplex co-delivering Hf^4+^ radiosensitizers and siHIF-1α achieved TME remodeling via low-dose radiotherapy/ferroptosis synergy, increasing TAAs/DAMPs release to convert cold tumors and establish T cell-mediated immunity (Fig. 2g) [49]. Secondary tumor analysis revealed higher CD8^+^/CD4^+^ T cell infiltration and significant CD44^+^CD62L^−^ effector memory T cell expansion (Fig. 2h, i), demonstrating systemic immune activation capable of suppressing distant tumors and establishing durable immune memory.

**Fig. 3:**
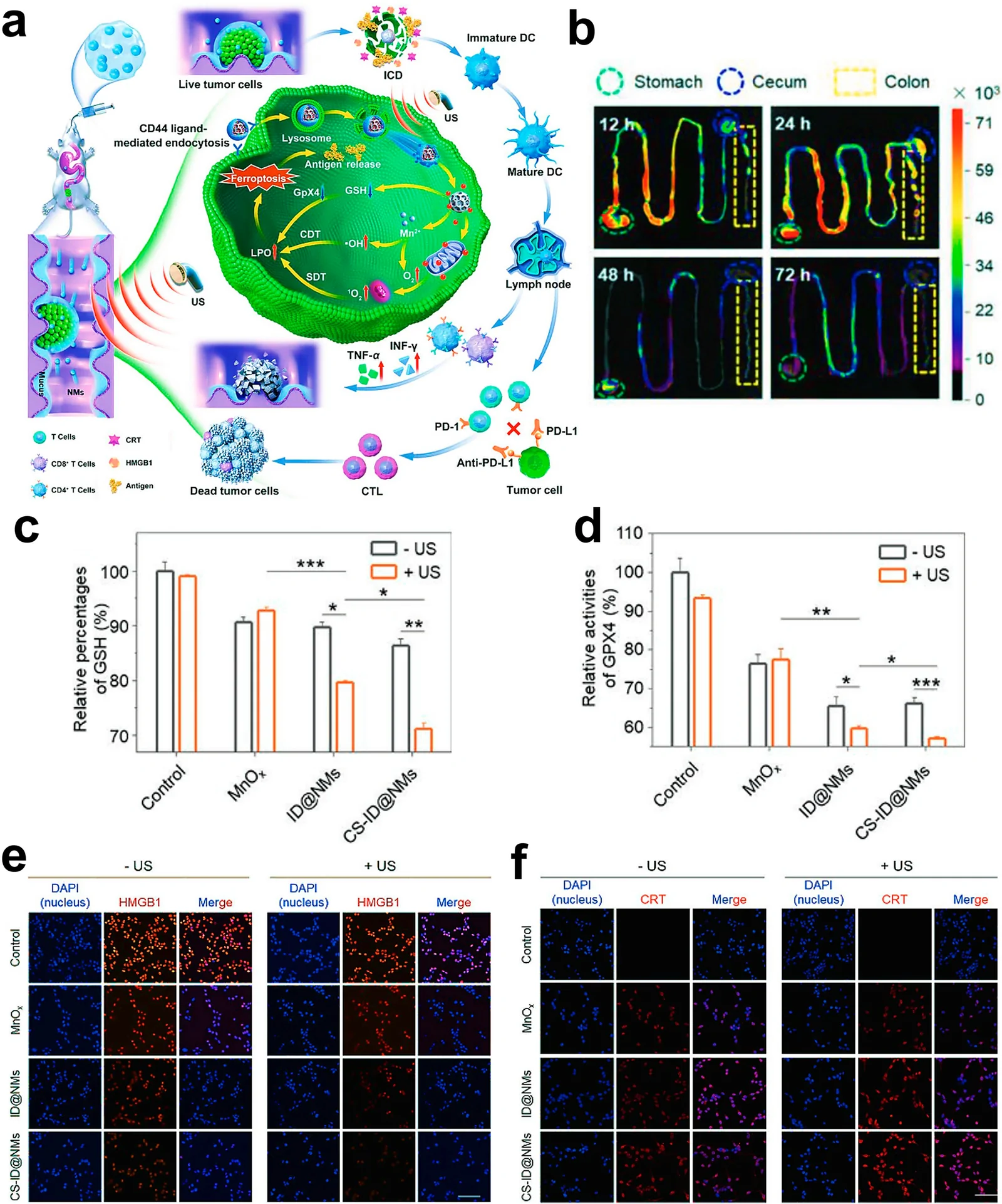
**a** Schematic of CS-ID@NMs multifunctional nanomotors demonstrating mucus penetration, deep tumor targeting, antitumor efficacy, and immune activation. **b** Ex vivo fluorescence imaging of gastrointestinal tracts (GITs) at 12–72 h post-administration of CS-ID@NM hydrogel. **c** Intracellular GSH quantification across treatment groups. **d** GPX4 activity levels in tumor cells under different treatments. CLSM images showing **e** HMGB1 release and **f** cell surface-exposed CRT (scale bar: 100 μm). Adapted with permission from [116], copyright 2022 Wiley–VCH GmbH, Weinheim

SDT induces cancer cell death through US-triggered ROS generation via sonosensitizer activation in oxygen-rich environments [173]. Previous studies demonstrate SDT's dual capability in promoting ICD and activating antitumor immunity [174]. Building upon these findings, a H_2_O_2_- and US-driven mesoporous manganese-based nanomotor (CS-ID@NMs) that leverages the TME' excess H_2_O_2_ through Mn^2+^-mediated Fenton-like reactions was engineered [116]. This process simultaneously generates ·OH for CDT and oxygen to potentiate SDT efficacy, creating a synergistic ferroptosis-inducing mechanism. The combined oxidative stress effectively disrupts tumor cell redox homeostasis, promoting antigen release and enhancing antitumor immune responses (Fig. 3a). The O_2_ microbubble propulsion system enables deep tumor penetration through mucus barriers under ultrasound guidance, with CD44-targeted chondroitin sulfate ligands facilitating selective tumor cell internalization. NIR fluorescence imaging revealed effective colonic accumulation 24 h post-administration followed by gradual signal diminution (Fig. 3b), confirming successful oral delivery to the colon region. Therapeutic assessment demonstrated CS-ID@NMs + US treatment significantly depleted intracellular GSH and suppressed GPX4 activity compared to controls (Fig. 3c, d), demonstrating robust ferroptosis induction. Histopathological evaluation through H&E staining, TUNEL assay, and Ki67 immunohistochemistry revealed marked tumor regression in colon adenocarcinoma models. ICD biomarker analysis showed nuclear HMGB1 release (reduced red fluorescence intensity, Fig. 3e) concurrent with increased cell surface CRT expression (Fig. 3f), suggesting amplified ICD effects from the multimodal therapeutic strategy. These findings collectively validate the nanomotor's capacity to synergize CDT, SDT, and ferroptosis pathways for enhanced immunotherapeutic outcomes.

**Fig. 4:**
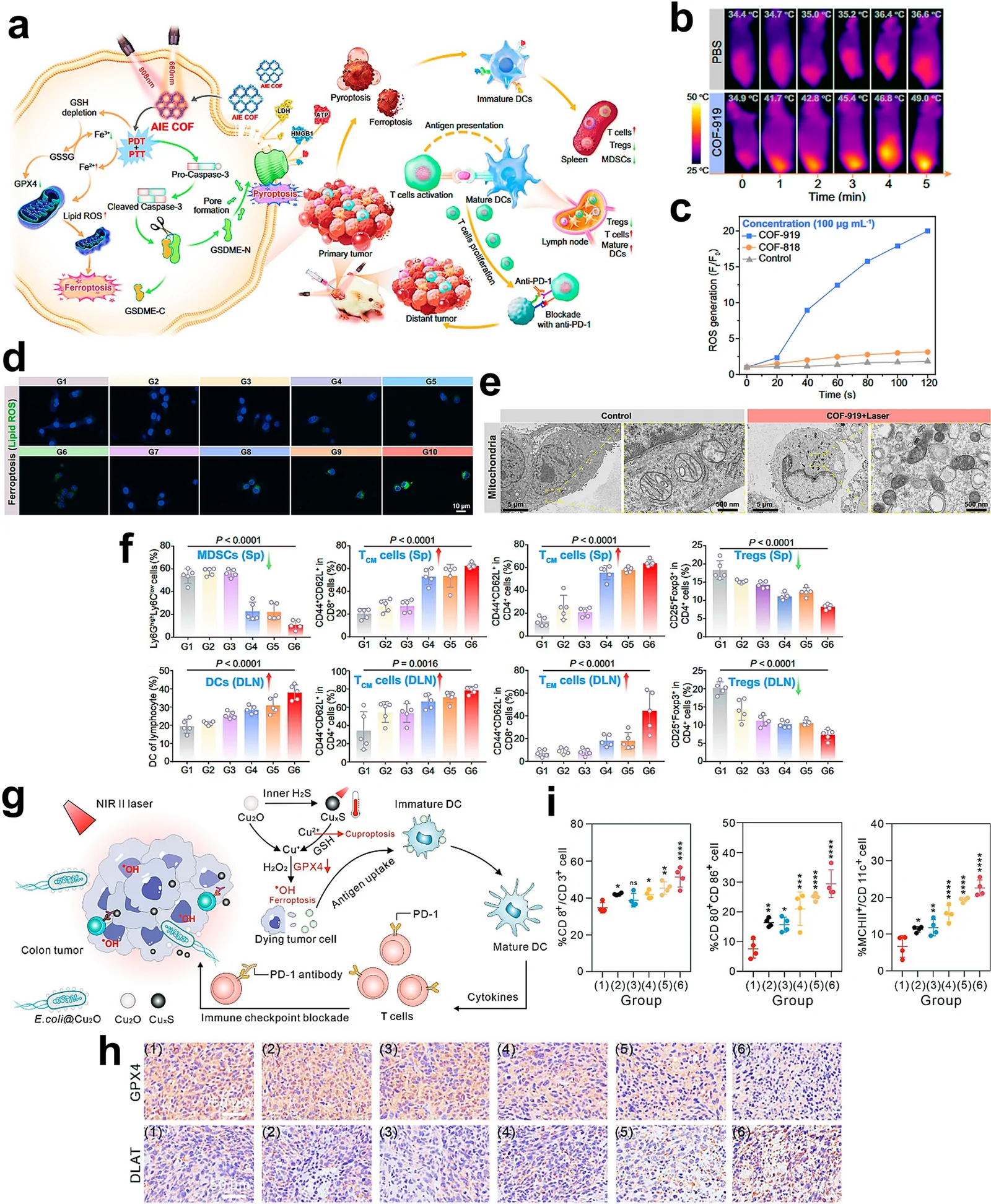
**a** Integration of planar/twisted motifs in COFs enables pyroptosis–ferroptosis-primed cancer immunotherapy. **b** In vivo thermal imaging of control vs COF-919 at different post-irradiation intervals. **c** Intracellular ROS generation analysis. **d** Confocal imaging of lipid peroxidation in 4T1 cells using BODIPY581/591-C11 probe (green). **e** Mitochondrial ultrastructure in normal vs COF-919-treated cells by bio-TEM. **f** Quantitative immune cell profiling: DCs in DLNs; TCM/TEM cells in spleen/DLNs; Tregs and MDSCs in spleen. Adapted with permission from [52], copyright 2023 Springer Nature Limited. **g** Design of *E. coli*@Cu_2_O nanohybrid for TME-activatable NIR-II PTT-enhanced ferroptosis/cuproptosis immunotherapy. **h** Immunohistochemical detection of GPX4 and DLAT expression in treated tumors. **i** Comparative analysis of splenic CTLs (CD3^+^CD8^+^CD4^−^), DC maturation (CD11c^+^CD80^+^CD86^+^), and activated DCs (CD11c^+^MHCII^+^) in tumor-draining lymph nodes. Adapted with permission from [59], copyright 2024 Wiley–VCH GmbH, Weinheim

PTT utilizes NIR laser-induced thermal effects to ablate tumors through light-absorbing materials. The rapid necrosis caused by PTT generates TAAs and enhances DC maturation via ICD activation and DAMPs release [175]. PDT, another light-based modality, employs photosensitizers under visible light to convert oxygen into cytotoxic ROS while inducing tumor ICD. Integrating PTT and PDT into a unified nanotherapeutic platform demonstrates synergistic enhancement of ICD induction. For instance, Zhang et al. developed a dual pyroptosis and ferroptosis inducer, COF-919, by integrating planar and twisted motifs with AIE characteristics into a COF [52]. This system combines PTT and PDT to trigger GPX4-related ferroptosis and GSDME-dependent pyroptosis, releasing HMGB1, lactate dehydrogenase, and ATP to stimulate robust immune responses (Fig. 4a). Under 808 nm irradiation, COF-919 demonstrates exceptional photothermal conversion efficiency (Fig. 4b), while 660 nm irradiation induces substantial ROS generation (Fig. 4c). In 4T1 cells treated with COF-919 under dual 660/808 nm irradiation, distinct bubble formation and intense lipid ROS fluorescence (Fig. 4d) accompanied by mitochondrial shrinkage in TEM images (Fig. 4e) confirmed concurrent pyroptosis and ferroptosis. Notably, COF-919-mediated phototherapy significantly increased DC populations and CD4^+^/CD8^+^ T cells in murine spleens/draining lymph nodes (DLNs), while reducing immunosuppressive MDSCs and regulatory T cells (Tregs) (Fig. 4f), indicating enhanced ICD-driven T-cell immunity. Cuproptosis, a newly identified regulated cell death mechanism involving copper binding to DLAT, induces DLAT aggregation, proteotoxic stress, and DAMPs release for antitumor immunity [176]. Strategies combining PTT with cuproptosis/ferroptosis show promise for ICD amplification. An engineered *E. coli*@Cu_2_O microbial nanohybrid synergizes NIR-II laser-enhanced PTT with GPX4 inactivation (ferroptosis) and DLAT aggregation (cuproptosis), effectively suppressing colon tumor recurrence/metastasis through ICD-mediated immunity (Fig. 4g) [59]. Immunohistochemical analysis revealed GPX4 suppression and DLAT aggregation in tumors treated with *E. coli*@Cu_2_O plus 1064 nm laser (Fig. 4h), achieving complete tumor eradication in vivo. Flow cytometry demonstrated elevated CTLs in spleens and activated DCs in DLNs (Fig. 4i), confirming robust antitumor immunity in MC38 tumor-bearing mice through PTT-enhanced ferroptosis/cuproptosis synergy.

**Fig. 5:**
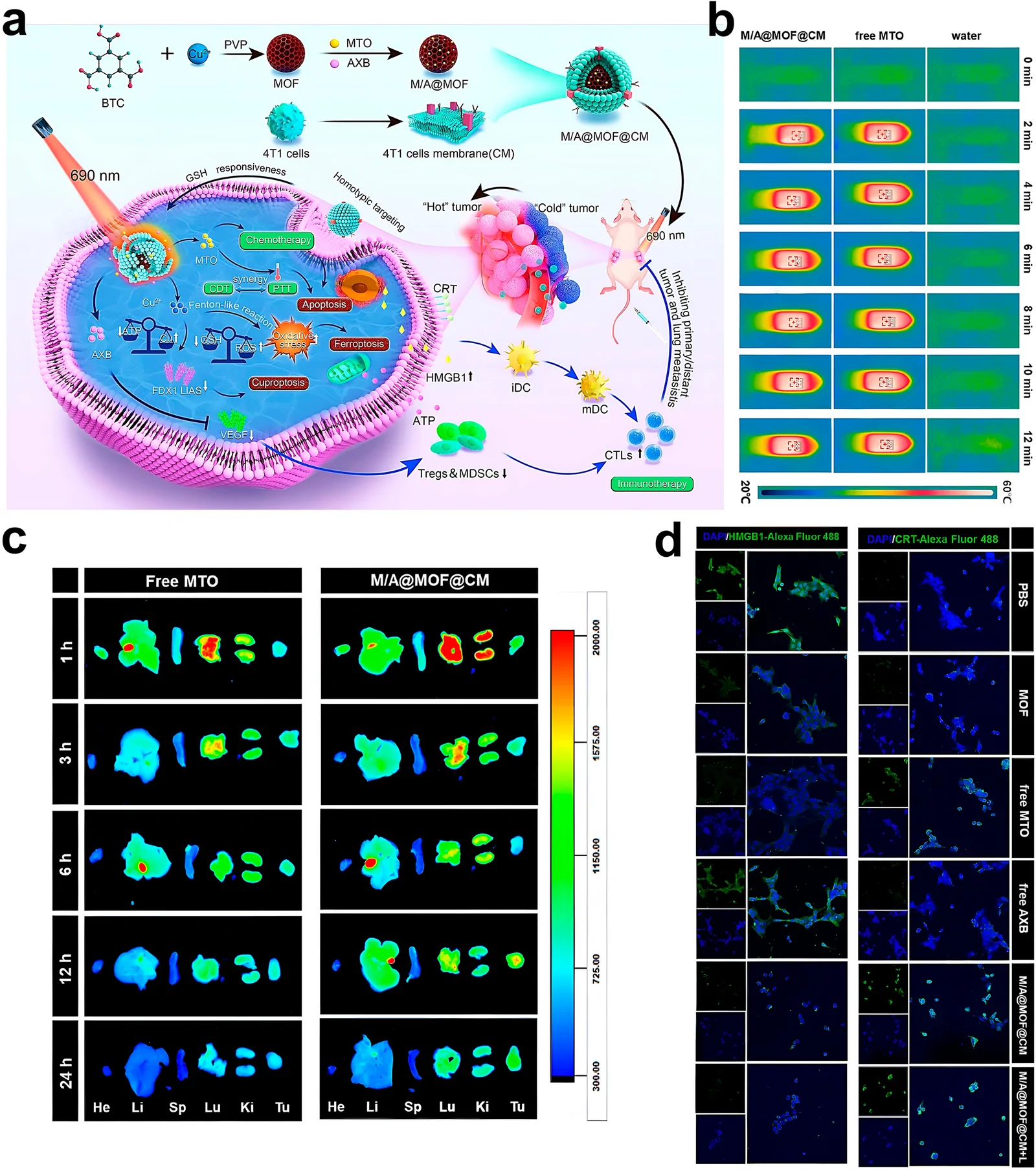
**a** Synthesis scheme of M/A@MOF@CM nanoplatform and its therapeutic mechanism in murine cancer models. **b** Thermal imaging comparison of water, free MTO, and M/A@MOF@CM under 0.82 W cm^−2^ irradiation (12 min). **c** Ex vivo fluorescence tracking of tumors/organs post-intravenous injection of free MTO vs M/A@MOF@CM. **d** Confocal microscopy visualization of HMGB1 translocation and CRT exposure. Adapted with permission from [177], copyright 2024 Elsevier B.V

Moreover, Ji et al. engineered a tumor cell membrane-coated Cu-MOF nanoparticle (M/A@MOF@CM) co-loaded with mitoxantrone (MTO) and axitinib (AXB) for synergistic chemotherapy, PTT, and CDT [177]. This multimodal platform simultaneously triggers cuproptosis, ferroptosis, and apoptosis through all-side tumor targeting, promoting DAMPs release and ICD induction while suppressing Tregs and MDSCs via VEGF pathway inhibition, effectively eliminating primary/secondary 4T1 tumors and lung metastases (Fig. 5a). MTO's inherent photothermal conversion capability enables dual chemotherapy-PTT functionality and thermal imaging capacity (Fig. 5b). In vivo fluorescence imaging revealed superior tumor accumulation of M/A@MOF@CM within 12 h post-administration (Fig. 5c), demonstrating enhanced homologous targeting. The M/A@MOF@CM + L group exhibited marked tumor regression, confirming the therapeutic synergy of combined modalities. Immunofluorescence analysis showed decreased intracellular HMGB1 with concurrent CRT membrane exposure in treated 4T1 cells (Fig. 5d), validating ICD activation. Flow cytometry confirmed enhanced DC maturation, elevated CTLs, and reduced Treg infiltration, evidencing reinforced antitumor immunity through amplified ICD induction.

##### Ferroptosis Amplification Driving ICD Activation

**Fig. 6:**
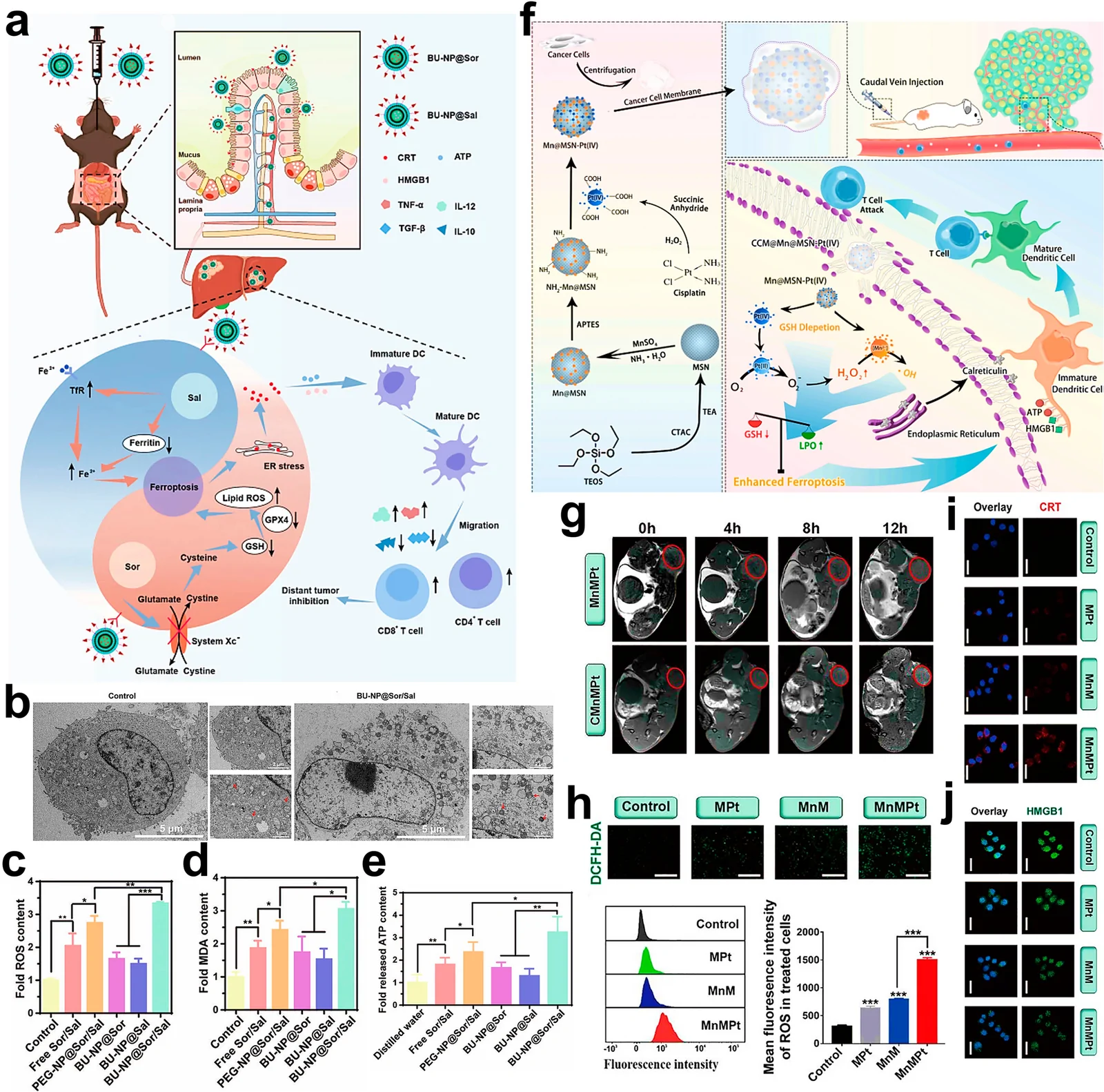
**a** Schematic of BU-NP@Sor/Sal demonstrating tumor-targeted accumulation for enhanced ferroptosis/ICD-mediated antitumor efficacy. **b** TEM images of Hepa1-6 cells showing mitochondrial ultrastructural changes (arrows). **c, d** Quantification of intracellular ROS levels and MDA concentrations across groups. **e** Extracellular ATP levels measured by ELISA assay. Adapted with permission from [85], copyright 2023 Wiley–VCH GmbH, Weinheim. **f** Design of CCM@Mn@MSN-Pt(IV) nanocomposites enabling GSH depletion, H_2_O_2_ self-supply, and homologous targeting for amplified ferroptosis-mediated ICD. **g** In vivo T1-weighted MRI of tumor-bearing mice pre-/post-injection (0–12 h) of MnMPt vs CMnMPt. **h** ROS detection by fluorescence imaging and flow cytometry quantification. **i**, **j** CLSM visualization of CRT membrane translocation and HMGB1 nuclear release in treated MFC cells. Adapted with permission from [96], copyright 2023 Elsevier B.V

Sal potentiates ferroptosis through coordinated upregulation of iron regulatory protein 2 (IRP2) and TfR1 with concomitant ferritin downregulation, elevating intracellular iron to potentiate ferroptosis [4]. This mechanistic synergy positions Sal combination therapy with other ferroptosis inducers as a strategy for enhanced ferroptotic cell death. The butyrate-conjugated PLGA nanoparticle BU-NP@Sor/Sal exemplifies this approach through dual mechanisms: sorafenib (Sor) induces system Xc^−^ inhibition and GSH depletion, while Sal elevates intracellular iron, creating a synergistic ferroptosis–ICD axis that enhances antitumor immunity and suppresses distal tumor growth (Fig. 6a) [85]. BU-NP@Sor/Sal treatment demonstrated characteristic mitochondrial contraction (Fig. 6b)—hallmarks of ferroptotic progression, accompanied by significantly elevated ROS and LPO levels versus BU-NP@Sor controls (Fig. 6c, d). ICD marker analysis three days post-treatment revealed maximal CRT surface exposure, extracellular ATP secretion (Fig. 6e), and HMGB1 release in BU-NP@Sor/Sal groups, correlating with peak CD8^+^ T-cell infiltration, confirming superior ICD induction versus monotherapies. Expanding this paradigm, cisplatin precursor Pt(IV) exhibits dual ferroptosis-enhancing capabilities: nuclear DNA damage-mediated cytotoxicity and GSH depletion through Pt(II) conversion, which activates NADPH oxidase (NOX) to generate Fenton reaction substrates. This mechanistic foundation inspired development of gastric cancer membrane-coated, Mn-doped MSNs (CMnMPt) co-delivering Pt(IV) [96]. The system leverages tumor-specific GSH reduction of Pt(IV) to Pt(II), amplifying H_2_O_2_ production while Mn^2+^ catalyzes •OH generation through Fenton-like reactions, creating a self-amplifying ferroptosis–ICD cycle (Fig. 6f). CMnMPt-NPs demonstrated superior homologous targeting through T_1_-weighted MRI signal enhancement within 4 h post-injection (Fig. 6g). MnMPt-NP treatment induced significant GPX4 suppression and ROS increase (Fig. 6h) alongside CRT membrane translocation (Fig. 6i), nuclear-to-extracellular HMGB1 redistribution (Fig. 6j), and amplified ATP release, qualitatively or quantitatively validating enhanced ICD induction.

**Fig. 7:**
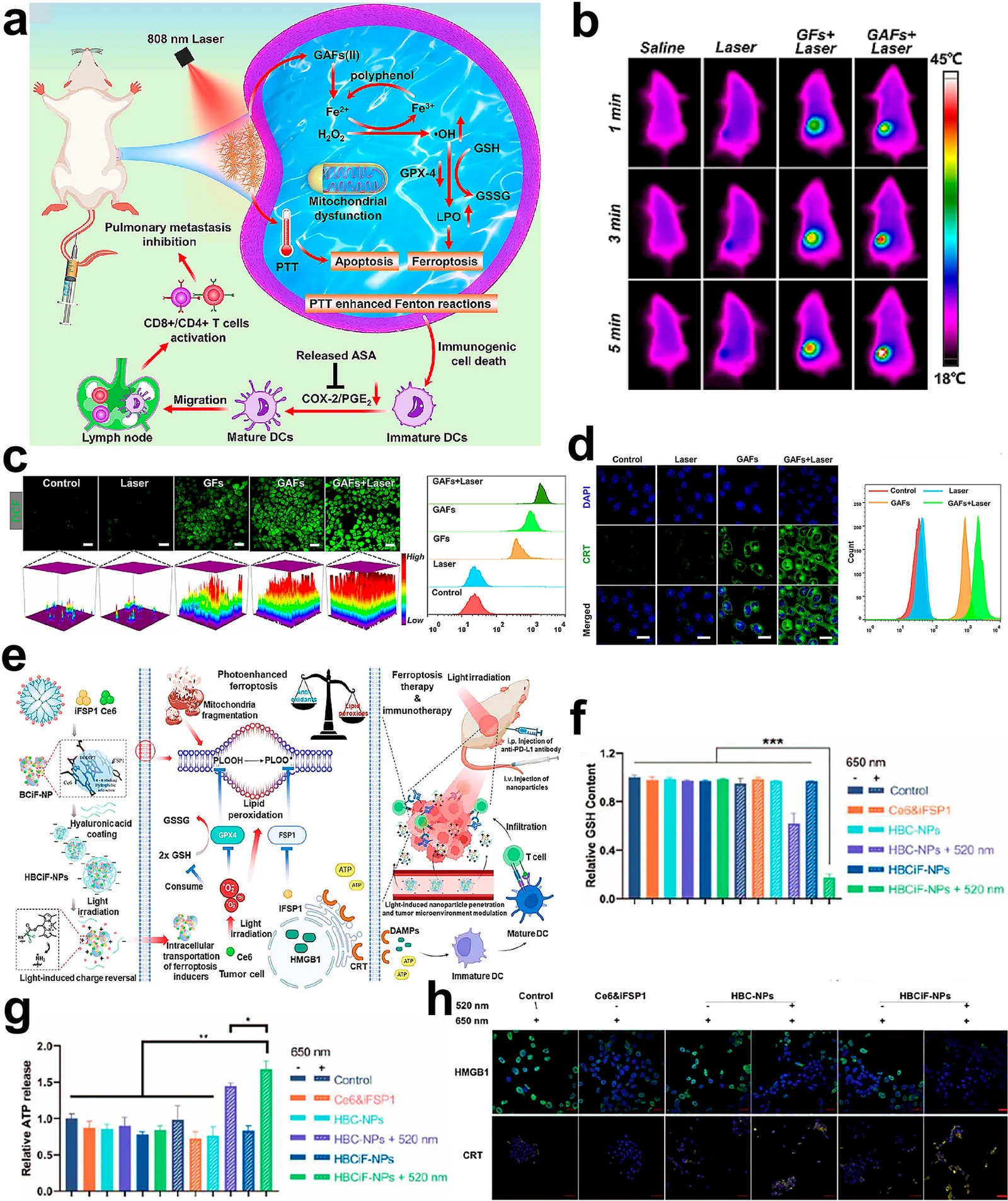
**a** Therapeutic mechanism of GAFs through COX-2/PGE2 axis suppression enabling PTT-enhanced ferroptosis–immunotherapy under 808 nm irradiation. **b** Real-time photothermal imaging of tumors in mice during 5-min irradiation across treatment groups. **c** Confocal microscopy with 3D intensity profiles showing ROS generation in treated cells. **d** CRT surface exposure in 4T1 cells treated with GAFs ± laser by confocal imaging and flow cytometry. Adapted with permission from [93], copyright 2023 Elsevier B.V. **e** Synergistic strategy combining light-activated ferroptosis (via HBCiF-NPs charge reversal) with PD-L1 blockade: PDT-induced GPX4 inhibition/ROS accumulation synergizes with iFSP1-enhanced lipid peroxidation, driving ICD-mediated antitumor immunity. **f** Intracellular GSH levels in A549 cells across therapeutic regimens. **g** Extracellular ATP levels measured in different treatment groups. **h** Confocal imaging of nuclear-to-cytoplasmic HMGB1 translocation and surface CRT expression in treated A549 cells. Adapted with permission from [127], copyright 2023 Wiley–VCH GmbH, Weinheim

PTT-induced hyperthermia enhances Fenton reaction kinetics to boost ROS production while accelerating GSH depletion, thereby impairing LPO clearance and synergistically promoting ferroptosis and ICD. Capitalizing on this mechanism, a previous research developed a gallic acid-aspirin-Fe(II) coordination polymer nanofiber (GAFs) where PTT-mediated temperature elevation amplifies ferroptotic signaling to activate antitumor immunity (Fig. 7a) [93]. The GAFs + laser group achieved rapid tumor-specific hyperthermia (Δ*T* = 27 °C/5 min), confirming superior photothermal conversion (Fig. 7b). Laser-activated GAFs generated substantial •OH (Fig. 7c) while depleting intracellular GSH. 4T1 cells treated with GAFs/PTT demonstrated maximal CRT exposure via intense fluorescence signals (Fig. 7d) and elevated DC maturation markers (CD80/CD86), validating enhanced ICD induction. Furthermore, PDT-mediated ferroptosis enhancement operates through dual mechanisms: ROS-driven lipid peroxidation coupled with AKT-mTOR axis inhibition that downregulates GPX4, effectively blocking LPO clearance. Zhou et al. engineered hyaluronic acid-coated PAMAM nanoparticles (HBCiF-NPs) co-loading FSP1 inhibitor iFSP1 and photosensitizer Ce6 [127]. Light activation (650 nm) triggers Ce6-mediated ^1^O_2_ generation for GSH/GPX4 depletion while iFSP1 inhibits FSP1 to amplify LPO accumulation, establishing photo-enhanced ferroptosis that promotes DAMPs release, DC maturation, and T-cell responses (Fig. 7e). HBCiF-treated cells showed minimal GSH/GPX4 levels post-irradiation (Fig. 7f), confirming ferroptosis potentiation. Concurrently, elevated surface CRT exposure (Fig. 7g), ATP secretion, and nuclear HMGB1 depletion (Fig. 7h) demonstrated enhanced ICD via light-amplified ferroptotic signaling.

**Fig. 8:**
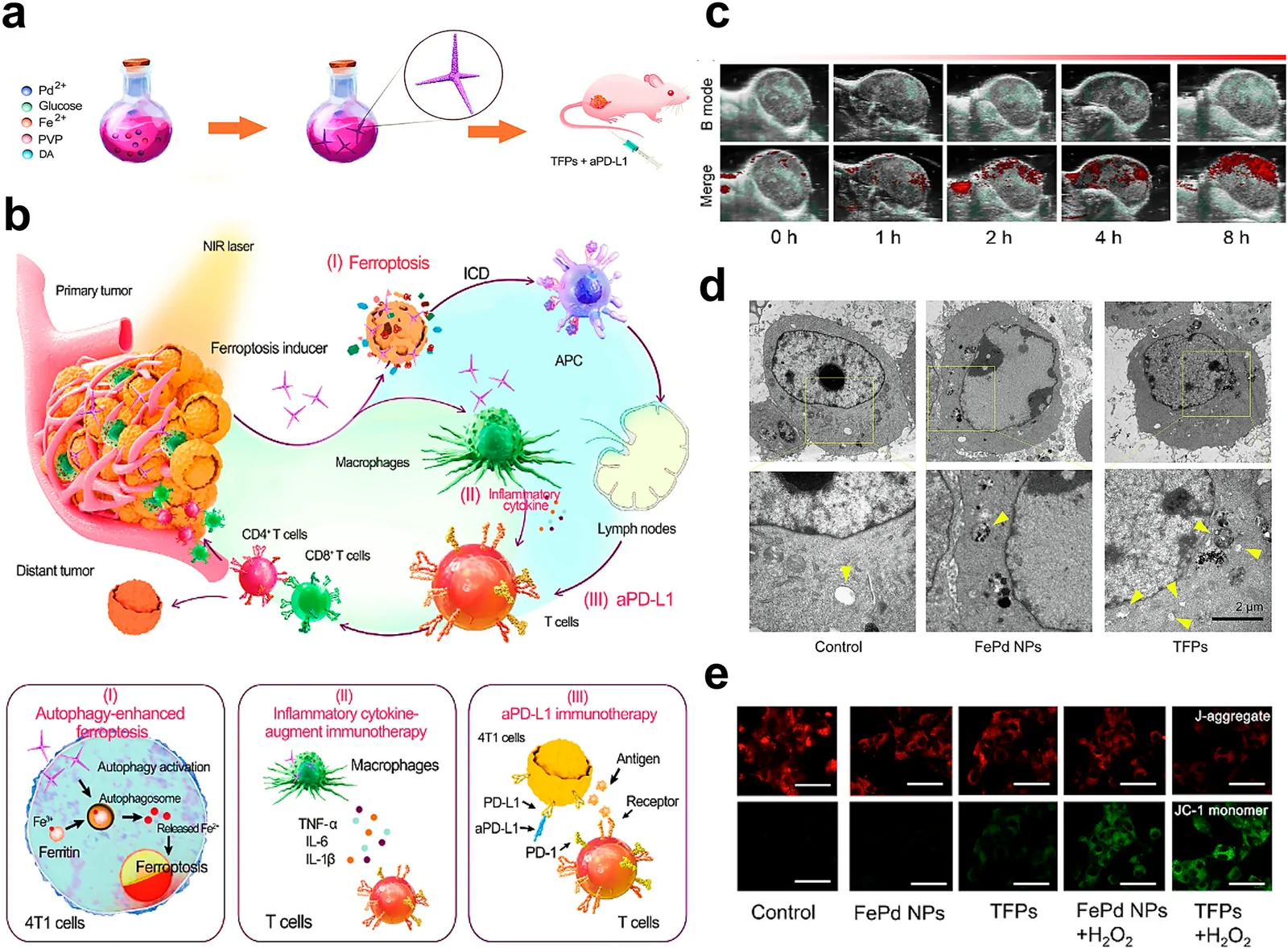
**a** Synthesis scheme of TFPs. **b** Design rationale: Spiky nanostructure-enabled TFPs for synergistic ferroptosis–immunotherapy enhancement. **c** In vivo PA imaging of 4T1 tumors at 0–8 h post-TFP injection. **d** Bio-TEM visualization of 4T1 cells post-treatments, indicating autophagosomes/autolysosomes (yellow arrows). **e** Mitochondrial depolarization in 4T1 cells assessed by JC-1 staining (CLSM). Adapted with permission from [5], copyright 2023 Science China Press

Given ferritin's role as the primary iron storage protein, targeted induction of ferritinophagy (ferritin-selective autophagy) can liberate Fe^2+^ pools to potentiate tumor ferroptosis. Capitalizing on this mechanism, the tetrapod-spiked FePd nanoconstructs (TFP) with polyvinylpyrrolidone (PVP) surface modification (Fig. 8a) were engineered [5]. The unique quadripodal morphology induces morphology-dependent autophagy, driving ferritin degradation to amplify ferroptosis–ICD signaling and suppress both primary and metastatic tumors (Fig. 8b). Post-TFP injection, photoacoustic signal intensity tripled within 8 h (Fig. 8c), demonstrating superior PA-guided therapeutic monitoring capability. Bio-TEM revealed significant autolysosome and autophagic vesicle accumulation in TFP-treated 4T1 cells versus controls (Fig. 8d), confirming morphology-enhanced autophagic flux. The TFP + H_2_O_2_ group exhibited markedly declined mitochondrial membrane potential (JC-1 fluorescence) than FePd NPs + H_2_O_2_ (Fig. 8e), validating ferritin degradation-driven ferroptosis amplification after autophagy. Combination therapy (TFP + NIR + αPD-L1) achieved 78% distant tumor inhibition versus 49% for FePd-based controls, establishing that TFP's spiked architecture synergizes with checkpoint blockade through enhanced ICD and antitumor immunity.

#### cGAS-STING Signaling Activation

The cGAS-STING signaling axis plays a pivotal role in cytoplasmic DNA surveillance. When cyclic GMP-AMP synthase (cGAS) detects aberrant dsDNA, it catalyzes cyclic GMP-AMP (cGAMP) synthesis. This secondary messenger binds the stimulator of interferon genes (STING) dimers, inducing conformational changes that facilitate Tank-binding kinase 1 (TBK1) recruitment and subsequent interferon regulatory factor 3 (IRF3) phosphorylation, ultimately driving IFN-β production. This cascade initiates critical immune responses including DC maturation, CD8^+^ T/NK cell activation, and IFN-γ upregulation [178]. Ferroptosis contributes to this pathway through dual mechanisms: mitochondrial damage releases mtDNA while cellular debris provides cytoplasmic dsDNA fragments, collectively activating cGAS-STING signaling.

**Fig. 9:**
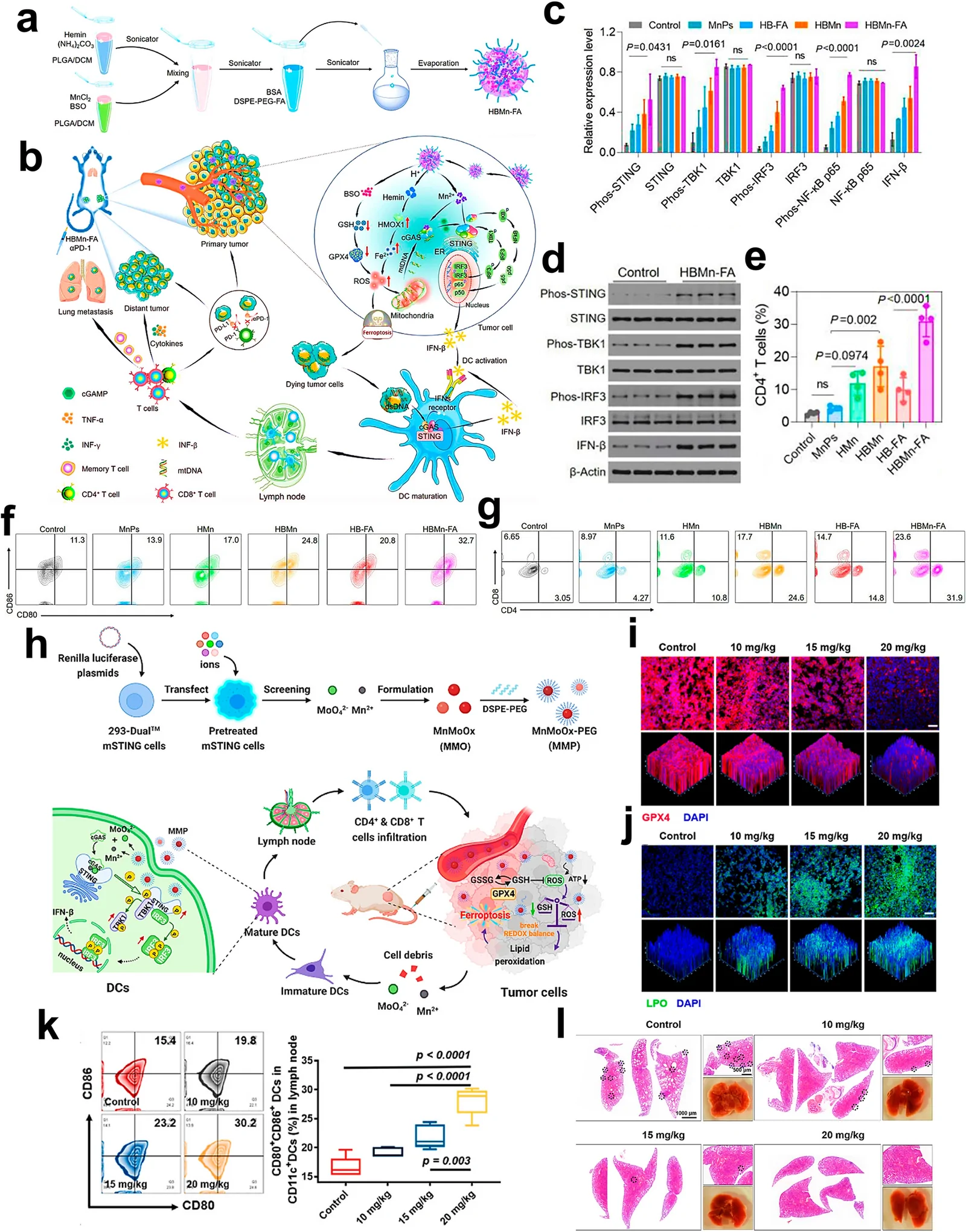
**a** Synthesis scheme of HBMn-FA nanoplatform. **b** Therapeutic mechanism: HBMn-FA activates cGAS-STING pathway to potentiate checkpoint blockade efficacy against primary/metastatic tumors. **c** Quantitative grayscale analysis of cGAS-STING pathway proteins (WB) in 4T1 cells post-treatment (mean ± SD, *n* = 3). **d** Western blot verification of cGAS-STING activation in DC2.4 cells. **e** Flow cytometry analysis of tumor-infiltrating CD4^+^ T cells across treatment groups. **f** DC maturation profiling (CD80^+^/CD86^+^) in tumor-draining lymph nodes (TDLNs). **g** Tumor-infiltrating CD4^+^/CD8^+^ T cell subpopulations quantified by flow cytometry. Adapted with permission from [164], copyright 2023 Science China Press. **h** Synthesis and mechanism of MMP NDs: Ferroptosis induction and cGAS-STING activation for tumor-specific immunity. Immunofluorescence staining of **i** LPO and **j** GPX4 expression in tumor sections. **k** Flow cytometry of DC maturation markers (CD80^+^/CD86^+^) in TDLNs. **l** H&E-stained lung sections from mice receiving MMP NDs (i.v. 10–20 mg/kg) vs control. Adapted with permission from [62], copyright 2024 KeAi Communications Co. Ltd

Metal ions modulate this pathway through distinct mechanisms. Mn^2+^ enhances cGAS enzymatic activity by increasing DNA-binding sensitivity and stabilizing cGAS-DNA complexes, enabling cGAMP production even at suboptimal DNA concentrations [179]. Furthermore, Mn^2+^ strengthens cGAMP-STING binding affinity, amplifying downstream signaling. Liang et al. engineered a multifunctional nanoplatform (HBMn-FA) incorporating Hemin, BSO, and Mn^2+^ within FA-modified PLGA polymers (Fig. 9a) [164]. This design achieves dual activation: (1) Ferroptosis induced by BSO/Hemin generates mitochondrial ROS, causing mtDNA release that synergizes with Mn^2+^ to prime cGAS-STING signaling; (2) Ferroptotic debris-derived dsDNA activates DC cGAS-STING pathways, promoting antigen presentation (Fig. 9b). Semiquantitative analysis of Western blot confirmed significant upregulation of p-STING, p-IRF3, and IFN-β in HBMn-FA-treated 4T1 cells, with Mn^2+^ potentiating pathway activation (Fig. 9c). Co-culture experiments demonstrated HBMn-FA-treated tumor cell supernatants enhance DC2.4 cell phosphorylation cascades (p-STING, p-TBK1, p-IRF3) and IFN-β secretion (Fig. 9d). Flow cytometry revealed 32.7% DC maturation efficiency and increased tumor-infiltrating CD8^+^/CD4^+^ T cell populations (Fig. 9e–g), confirming systemic immune activation. Another study identified Mn^2+^ and MoO_4_^2−^ as potent cGAS-STING activators. Their synergistic potential inspired manganese molybdate nanoparticles (MMP NDs) that simultaneously induce ferroptosis (via Mo^6+^/Mn^4+^-mediated GSH depletion and GPX4 inhibition) and activate DNA sensing pathways (Fig. 9h, i) [62]. MMP NDs demonstrated dual functionality: inducing LPO accumulation (ferroptosis hallmark) (Fig. 9j) while promoting DC maturation (Fig. 9k), and CD8^+^ T cell activation effectively suppressing primary/metastatic tumors (Fig. 9l). Besides, Zn^2+^ demonstrates additional regulatory capacity by stabilizing cGAS-DNA condensates [180]. This property was exploited in ZIF-8 nanoparticles co-loaded with U-104 and siNFS1, where Zn^2+^ synergizes with Cu^2+^-enhanced ROS to induce mitochondrial DNA damage beyond repair thresholds [163]. The resultant mtDNA release robustly activates cGAS-STING pathways, combining with ferroptosis-induced ICD to enhance DC maturation and T cell infiltration for potent antitumor immunity.

**Fig. 10:**
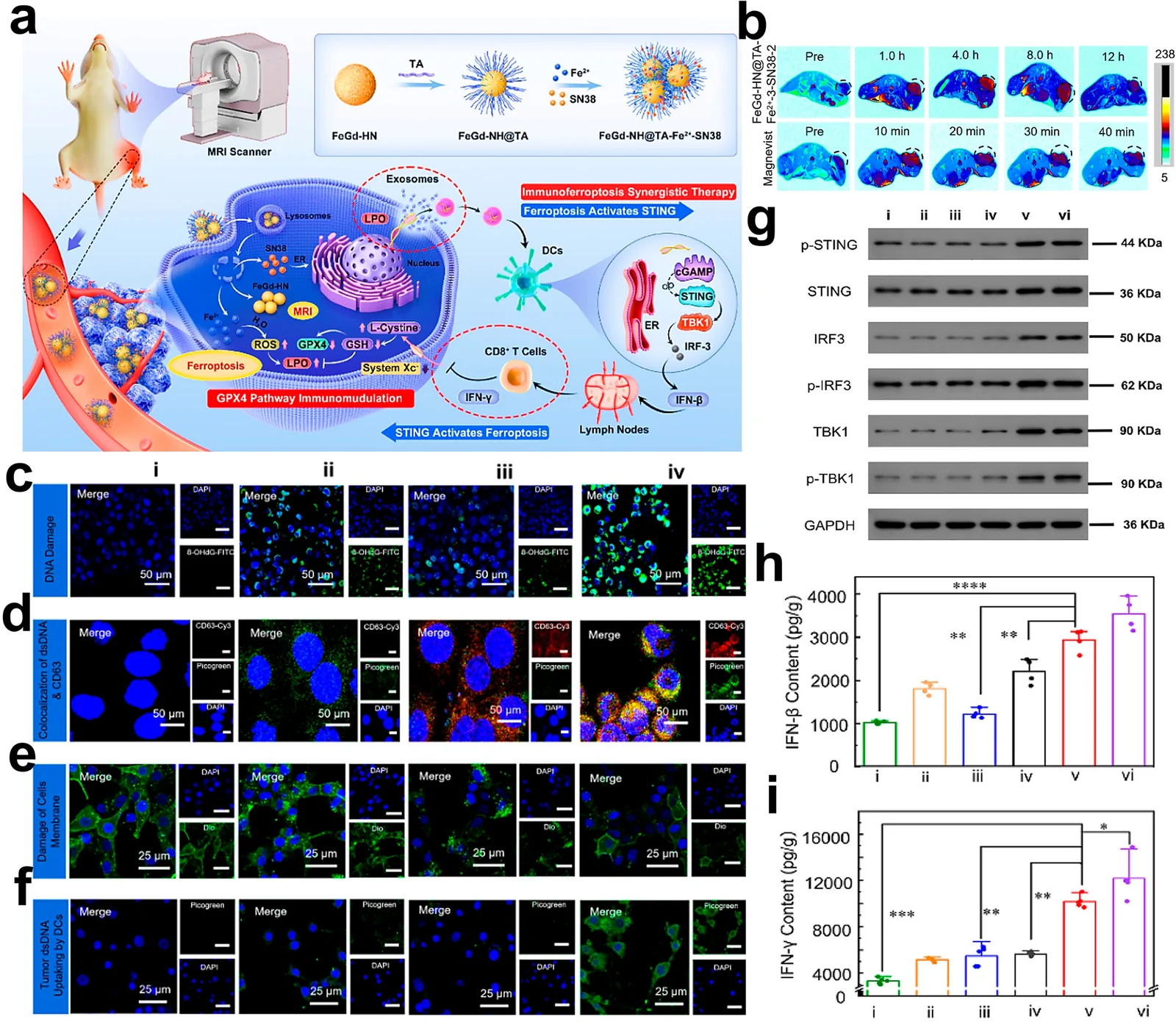
**a** Synthesis of FeGd-HN@TA-Fe^2+^-SN38 and therapeutic mechanism via GPX4/STING dual-pathway cross-talk for immunoferroptosis therapy. **b** T1-weighted MRI of 4T1 tumors in BALB/c mice post-injection of FeGd-HN@TA-Fe^2+^-SN38 (Fe/Gd dose: 5 mg/kg) vs Magnevist® (Gd dose: 5 mg/kg). Confocal imaging of 4T1 cells treated with (i)–(iv): **c** DNA damage (8OHdG, green)/nuclear co-staining (DAPI, blue); **d** Exosome marker CD63 (red)/nucleic acid staining (PicoGreen, green); **e** Membrane integrity (DiO, green). **f** Bone marrow-derived DCs (BMDCs) cultured with conditioned media from (i)–(iv) treated cells (DAPI nuclear staining). **g** Western blot analysis of cGAS-STING pathway proteins (p-STING, TBK1, IRF3) in tumors from groups (i)–(vi). ELISA quantification of **h** IFN-βand **i** IFN-γ in tumor tissues across groups (i)–(vi). Adapted with permission from [165], copyright 2023 Elsevier Ltd

In contrast with metal-ion-mediated STING activation, organic small-molecule drugs that trigger nuclear or mitochondrial DNA release have emerged as alternative activators of the cGAS-STING pathway. For example, Guo et al. developed Fe_3_O_4_/Gd_2_O_3_ hybrid nanoparticles coated with TA and complexed with Fe^2+^ and SN38 (7-ethyl-10-hydroxycamptothecin) [165]. This system leverages SN38 to stimulate exocytosis of DNA-containing exosomes while Fe^2+^-mediated LPO accumulation induces plasma membrane rupture, synergistically enhancing exosome release and facilitating DNA fragment delivery to DCs for STING pathway activation (Fig. 10a). Intratumoral MRI signals at 4 h post-injection confirmed efficient nanoparticle accumulation in tumors (Fig. 10b). CLSM demonstrated that the FeGd HN@TA-Fe^2+^-3-SN38-2 group exhibited maximal DNA damage in 4T1 cells through combined ferroptosis and SN38 effects (Fig. 10c). Colocalization of dsDNA (green) with exosomal marker CD63 (red) verified DNA-containing exosome production (Fig. 10d). Concurrently, LPO-driven membrane disruption (Fig. 10e) significantly enhanced DC uptake of DNA fragments (Fig. 10f). Western blot analysis revealed elevated STING pathway activation markers (STING, p-STING, TBK1, p-TBK1, IRF3, p-IRF3) in groups v (FeGd HN@TA-Fe^2+^-3-SN38-2) and vi (combination with αPD-L1), confirming successful DC activation (Fig. 10g). Corresponding increases in tumor tissue IFN-β (Fig. 10h) and IFN-γ (Fig. 10i) levels validated STING-mediated DC maturation. Alternatively, psoralen directly binds nuclear DNA, inhibiting replication and inducing fragmentation to elevate cytoplasmic DNA levels for cGAS-STING activation [68]. As a photosensitizer, it generates singlet oxygen that synergizes with Fenton reaction-derived •OH to potentiate ferroptosis. This dual action promotes ICD through DAMPs release while STING activation drives IFN-β-mediated DC maturation and T-cell differentiation, establishing durable antitumor immunity. Separately, NRF2 inhibitor ML385 induces mitochondrial ROS bursts, causing membrane depolarization and mtDNA release to activate STING [37]. ML385 concurrently enhances ferroptosis-induced ICD and reprograms MDSCs toward a CTL-supportive phenotype via STING activation, amplifying antitumor immune responses.

#### Adjuvant-Driven Innate Immune Priming

As essential components of vaccines, immune adjuvants amplify the magnitude, breadth, and durability of immune responses through multifaceted mechanisms. These include DC activation, complement system engagement, and cytokine/chemokine modulation, collectively improving therapeutic outcomes in cancer treatment [181]. Recent advances integrate immune adjuvants (e.g., TLR agonists, aluminum-based adjuvants) with nanodelivery platforms to enhance APC efficiency during ferroptosis-mediated immunotherapy. This synergy accelerates T cell-driven antitumor immunity and establishes durable immune memory.

**Fig. 11:**
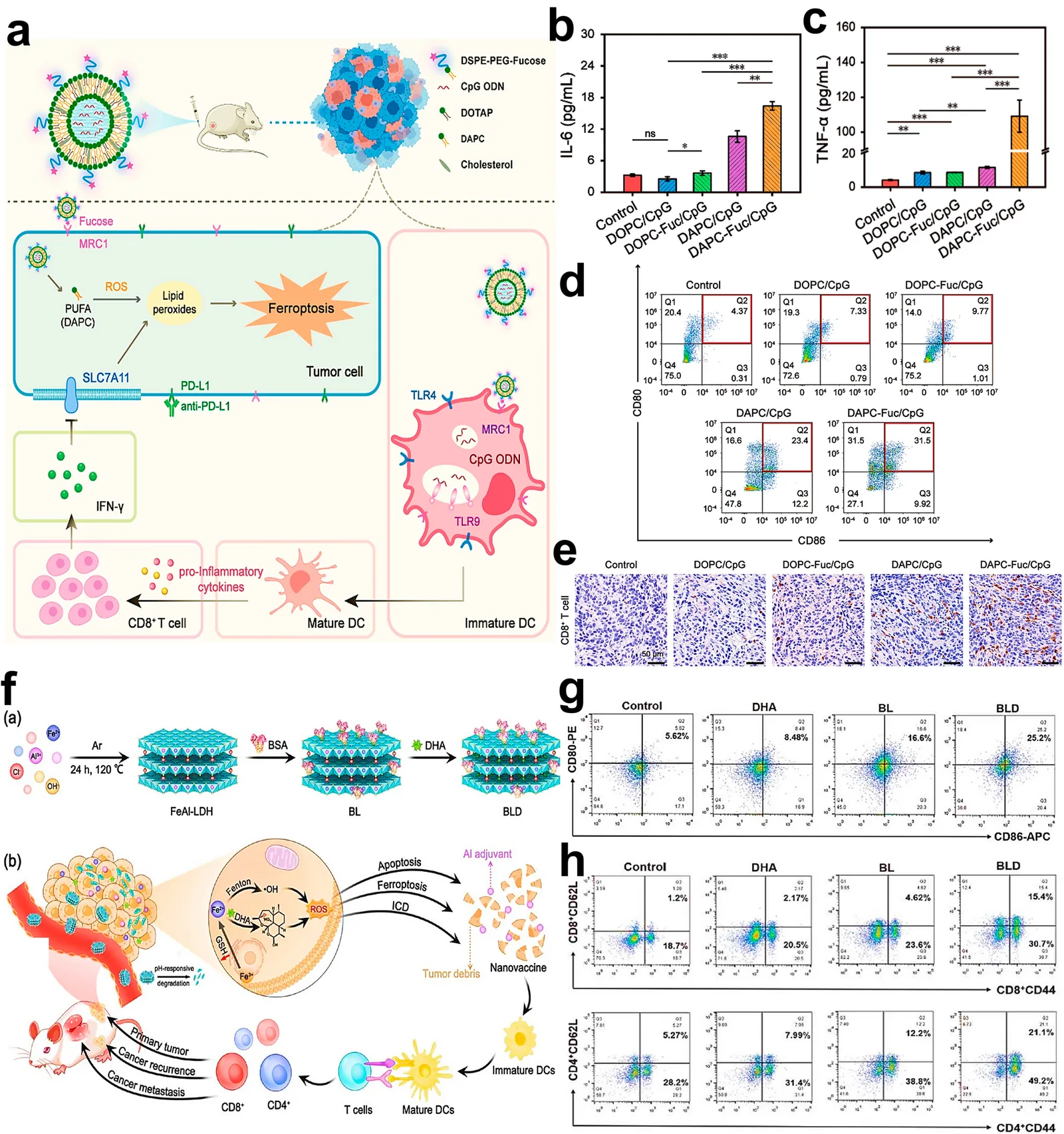
a Schematic of targeted liposomal platform enabling synergistic ferroptosis–immunotherapy. **b** Serum IL-6 and **c** TNF-α quantification post-treatment. **d** Splenic DC maturation profiling (CD80^+^/CD86^+^ ratio). **e** CD8^+^ T cell infiltration in tumors 1-day post-treatment (IHC imaging). Adapted with permission from [38], copyright 2024 Elsevier Ltd. **f** BLD construct synthesis and TME-responsive Fe^3+^/Al^3+^/DHA release mechanism. **g** Flow cytometry analysis of DC maturation (CD11c^+^CD80^+^/CD86^+^) in lymph nodes. **h** Memory T cell subsets (CD44^+^CD62L^±^) in splenic CD8^+^/CD4^+^ populations. Adapted with permission from [162], copyright 2024 American Chemical Society

Notably, CpG ODNs—synthetic TLR9 agonists—induce DC maturation, upregulate MHC-II expression, and stimulate Th1 polarization with pro-inflammatory cytokine production [181]. Building on this, Gao et al. engineered liposomes using DAPC lipids to encapsulate CpG ODNs [38]. Tumor-associated ROS triggered DAPC lipid peroxidation, simultaneously inducing ferroptosis and releasing CpG ODNs. These dual effects enhanced DC maturation and CD8^+^ T cell effector functions, creating a self-reinforcing adaptive immune loop (Fig. 11a). CpG supplementation significantly elevated serum IL-6 (Fig. 11b) and TNF-α (Fig. 11c), indicative of amplified pro-inflammatory signaling. Splenic CD80^+^/CD86^+^ DC populations increased markedly (Fig. 11d), correlating with expanded tumor-infiltrating CD8^+^ T cells (Fig. 11e). IFN-γ secretion from activated T cells further potentiated ferroptosis, establishing a positive feedback mechanism between immunotherapy and cell death pathways.

Aluminum adjuvants—the first clinically approved adjuvants—function via antigen adsorption to form depot complexes that sustain antigen exposure [182]. Though non-immunogenic, they enhance APC-mediated antigen phagocytosis by precipitating soluble antigens. In an innovative application, FeAl-layered double hydroxide (FeAl-LDH) nanoparticles delivered DHA as an in situ nanovaccine (BLD) [162]. Iron-catalyzed Fenton reactions and DHA activation exacerbated tumor oxidative stress, inducing ferroptosis and ICD. Concurrently, released TAAs complexed with aluminum adjuvants generated potent in situ vaccination (Fig. 11f). Histopathological analysis further revealed extensive structural damage in both primary and distal tumors following BLD treatment. Enhanced DC maturation in tumor-draining lymph nodes (Fig. 11g) and elevated CD8^+^/CD4^+^ memory T cell populations (Fig. 11h) confirmed sustained immune memory activation, underscoring the nanovaccine’s capacity to prevent recurrence and metastasis.

**Fig. 12:**
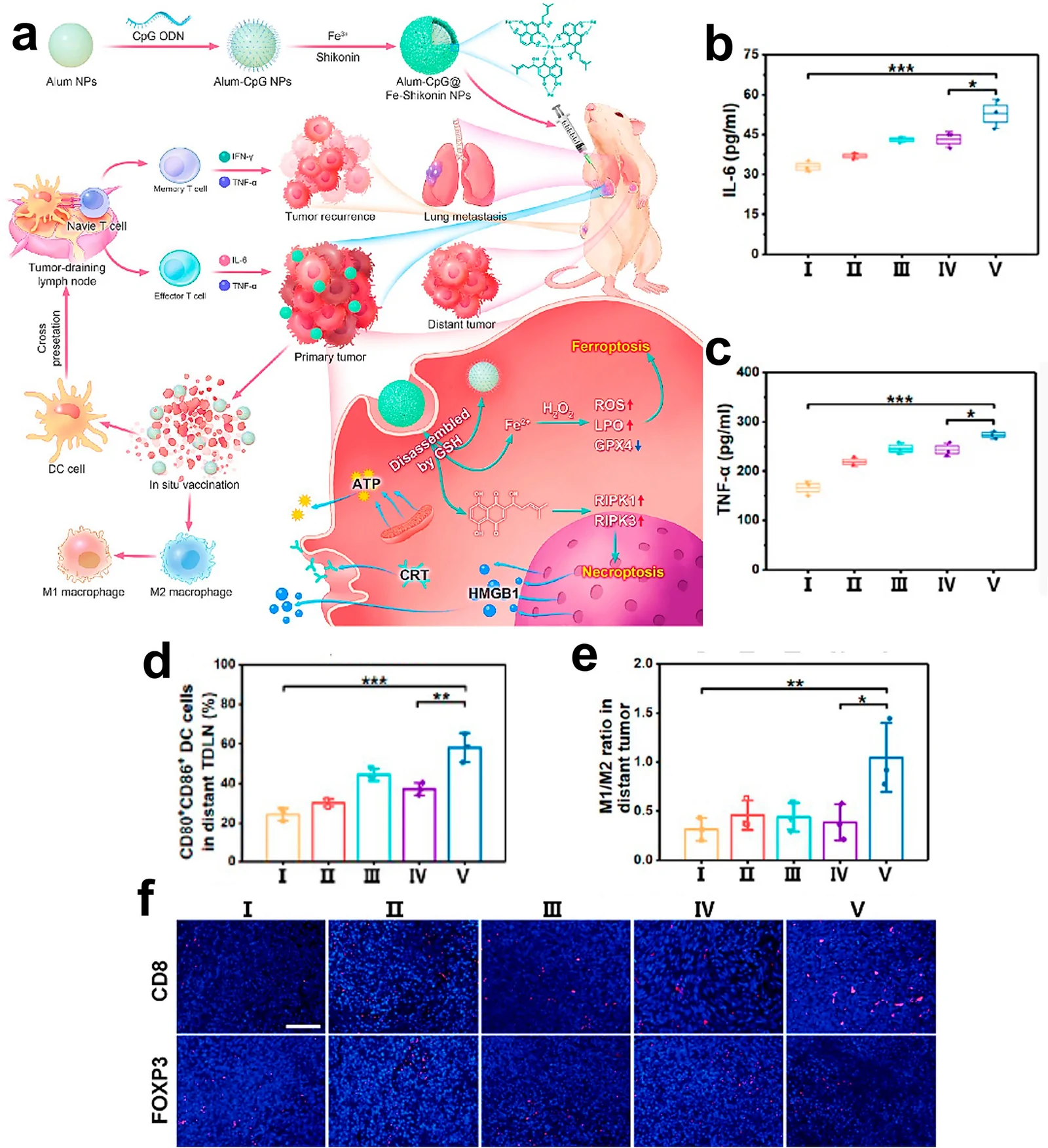
**a** Combination immunotherapy via autologous tumor lysates co-inducing immunogenic ferroptosis/necroptosis with adjuvant co-delivery. **b** Serum IL-6 and **c** TNF-α levels in 4T1 tumor-bearing mice across treatments (mean ± SD, *n* = 3). **d** Mature DCs (CD11c^+^CD80^+^CD86^+^) quantification in tumor-draining lymph nodes (TDLNs) by flow cytometry (*n* = 3). **e** M1/M2 macrophage polarization ratio (F4/80^+^CD206^−^CD86^+^/F4/80^+^CD206^+^CD86^−^) in distant tumors (*n* = 3). **f** Immunohistochemical staining of CD3^+^CD8^+^ T cells and Tregs (Foxp^3+^) in distant tumors. Adapted with permission from [183], copyright 2023 American Chemical Society

The strategic combination of alum-based adjuvants with CpG ODNs synergistically potentiates immune activation by improving antigen presentation efficiency and amplifying antitumor immunity. Illustrating this paradigm, Shi et al. developed alum-phosphate nanocarriers encapsulating CpG ODNs, surface-coated with an iron-shikonin metal–phenolic network (Alum-CpG@FeShikonin NPs), to create an in situ personalized nanovaccine [183]. Upon tumor cell internalization, the Fe-shikonin shell dissociates into Fe^2+^ and shikonin, jointly inducing ferroptosis and necroptosis-driven ICD. Tumor-derived antigens released during this process are captured by alum nanoparticles and co-delivered with CpG ODNs to professional APCs, initiating a multistage antitumor immune cascade (Fig. 12a). Systemic immune potentiation was evidenced by elevated serum levels of IL-6 and TNF-α (Fig. 12b, c). Leveraging the alum-CpG synergy, DC maturation in tumor-draining lymph nodes (TDLNs) was substantially enhanced (Fig. 12d). Critically, the nanovaccine skewed TAMs toward a pro-inflammatory M1 phenotype (Fig. 12e) and promoted robust infiltration of CTLs in distant tumors (Fig. 12f), demonstrating its capacity to reprogram the immunosuppressive TME and establish durable antitumor immunity.

### Disruption of Immunosuppressive Tumor Niches

#### Coordination with ICB-Based Therapy

Immune checkpoints operate through ligand–receptor interactions that deliver inhibitory signals to immune effector cells, suppressing antitumor immunity. While this mechanism safeguards self-tolerance and prevents autoimmune tissue damage, malignant cells co-opt these pathways to evade T cell-mediated immune surveillance [184]. Immune checkpoint blockade (ICB) therapies targeting CTLA-4, PD-1, or PD-L1 counteract this immune suppression by disrupting inhibitory signaling axes, thereby reinvigorating T cell function and potentiating systemic antitumor responses [185]. The combination of ferroptosis induction and ICB demonstrates therapeutic synergy through reciprocal mechanisms. Ferroptosis enhances tumor immunogenicity by promoting immune cell infiltration and converting immunologically inert (“cold”) tumors into responsive (“hot”) microenvironments, thereby improving ICB efficacy. Conversely, ICB compensates for ferroptosis limitations by mitigating immunosuppressive networks and establishing immunological memory, thereby reducing metastatic potential and preventing recurrence. We next discuss strategic approaches to optimize this ferroptosis–ICB synergy through coordinated therapeutic targeting.

##### PD-1/PD-L1 Axis Inhibition

The PD-1/PD-L1 immune checkpoint axis, where PD-1 is expressed on tumor-infiltrating immune cells and PD-L1 on tumor cells, plays a critical role in suppressing adaptive antitumor immunity by enabling tumor immune escape [186]. Current therapeutic strategies combine PD-1/PD-L1 antibodies with ferroptosis inducers to simultaneously block this immunosuppressive pathway and enhance antitumor immune responses [87, 148]. Beyond conventional antibodies, emerging research highlights the potential of PD-L1 antagonistic peptides, nucleic acids, enzymes, and small-molecule drugs as immune checkpoint inhibitors.

**Fig. 13:**
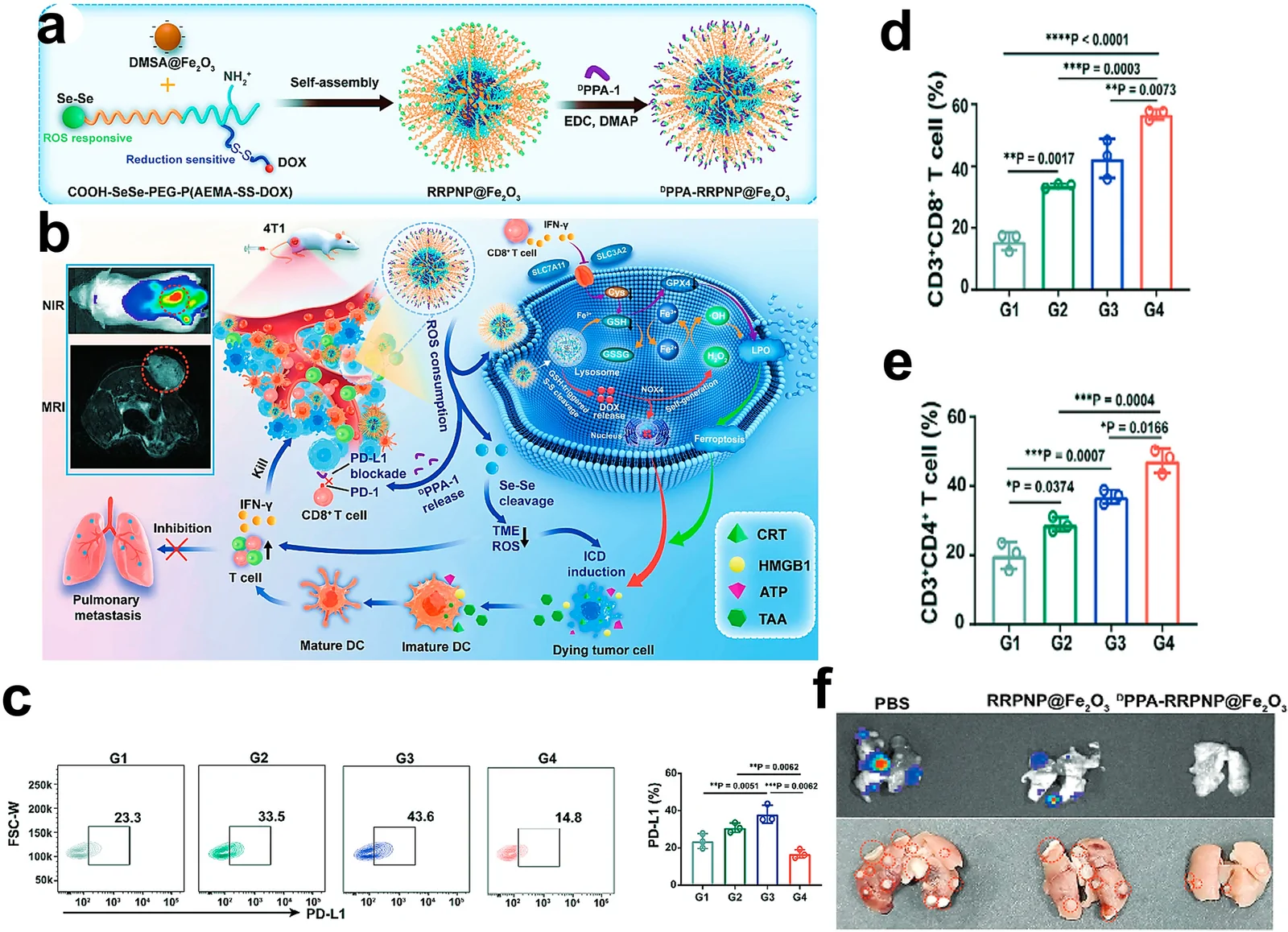
**a** Synthesis of ^D^PPA-RRPNP@Fe_2_O_3_. **b** ^D^PPA-RRPNP@Fe_2_O_3_ synergizes ROS scavenging, immune checkpoint blockade, and chemo-ferroptosis-mediated antitumor immunity. **c** Flow cytometry analysis of PD-L1 expression on tumor cells (mean ± SD, *n* = 3). Tumor-infiltrating **d** CD3^+^CD8^+^ and **e** CD3^+^CD4^+^ T cell quantification across treatments. **f** Lung metastasis detection via luciferase bioluminescence imaging at day 31. Adapted with permission from [118], copyright 2024 Wiley–VCH GmbH, Weinheim

Recent developments include a ferroptosis nanoplatform (^D^PPA-RRPNP@Fe_2_O_3_) constructed through electrostatic adsorption of PD-L1-blocking D-peptide (^D^PPA-1) onto DMSA@Fe_2_O_3_ nanoparticles (Fig. 13a) [118]. This system synergistically induces ICD and T-cell activation through combined ferroptosis and ICB, achieving superior tumor immunotherapy outcomes (Fig. 13b). In vivo studies demonstrated that ^D^PPA-RRPNP@Fe_2_O_3_ treatment significantly reduced tumor surface PD-L1 expression (Fig. 13c) and enhanced T-cell activation compared to RRPNP@Fe_2_O_3_ controls (Fig. 13d, e). The treatment group showed reduced tumor burden, extended survival, and suppressed pulmonary metastasis via ex vivo imaging (Fig. 13f), confirming the therapeutic synergy between ferroptosis and ICB.

**Fig. 14:**
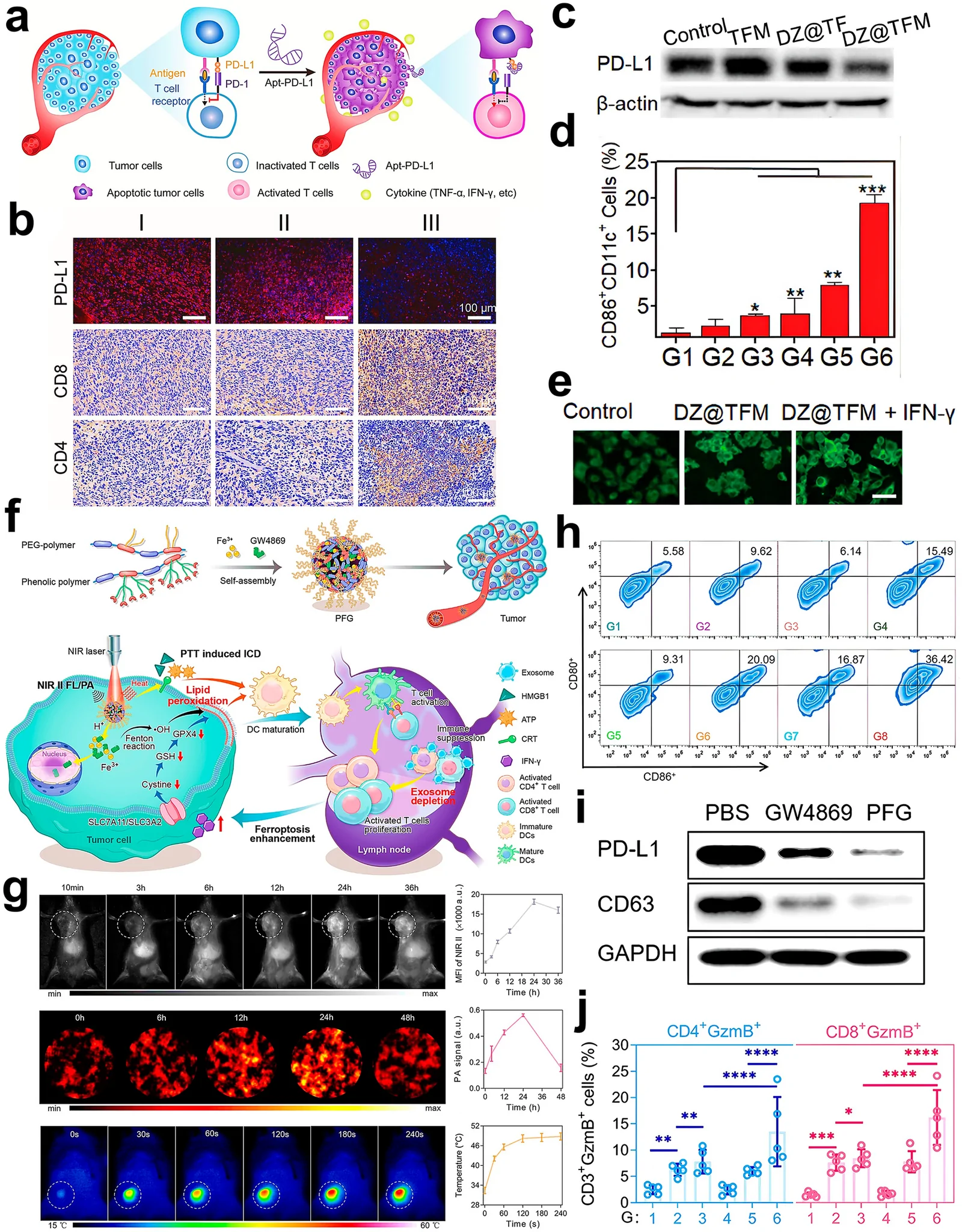
**a** Apt-PD-L1-mediated immune checkpoint blockade mechanism. **b** Immunofluorescence (IF) staining of PD-L1 and immunohistochemical analysis of CD4^+^/CD8^+^ T cell infiltration in tumor sections. Adapted with permission from [90], copyright 2022 Elsevier Ltd. **c** WB analysis of PD-L1 expression in B16F10 melanoma cells. **d** Quantitative assessment of DC maturation (CD80^+^/CD86^+^) in tumor-draining lymph nodes. **e** Fluorescence imaging of LPO levels post-IFN-γ pretreatment. Adapted with permission from [91], copyright 2022 Wiley–VCH GmbH, Weinheim. **f** PFG MPNs design: Photothermal imaging/therapy-mediated exosomal immunosuppression relief, ferroptosis potentiation, and immune activation. **g** In vivo NIR-II fluorescence imaging, PA imaging, and thermal imaging of PFG MPNs in tumors. **h** Flow cytometry analysis of mature DCs (CD11c^+^CD80^+^CD86^+^) in TDLNs. **i** WB detection of exosome-specific PD-L1 and CD63 in tumor-derived exosomes. **j** Flow cytometric profiling of GzmB^+^CD4^+^/CD8^+^ T cells (CD3^+^ gating) in TDLNs. Adapted with permission from [141], copyright 2022 American Chemical Society

Parallel studies explore PD-L1 DNA aptamers (Apt-PD-L1) for their high specificity and tissue penetration. Zhang et al. combined Apt-PD-L1 with ferroptosis-inducing nanoreactors to amplify TAA release and enhance immune infiltration (Fig. 14a, b) [90]. Another approach utilized anti-PD-L1 deoxyribozyme (DZ) loaded into Mn^2+^/Fe^3+^ hybrid metal–phenolic networks (DZ@TFM), which effectively silenced PD-L1 while inducing ferroptosis–immunotherapy [91]. DZ@TFM treatment significantly downregulated PD-L1 expression (Fig. 14c) and enhanced DC maturation through combined ferroptosis and PTT effects (Fig. 14d). Notably, IFN-γ elevation in DZ@TFM-treated cells amplified LPO levels (Fig. 14e), establishing a positive feedback loop between immune activation and ferroptosis.

Notably, several organic small molecules demonstrate effective PD-1/PD-L1 pathway blockade. A representative example involves engineering semiconductor polymer-based metal–phenolic networks (PFG MPNs) that co-encapsulate ferroptosis inducer Fe^3+^ and exosomal inhibitor GW4869 [141]. This PTT-active platform targets PD-L1 exosomal secretion, thereby reversing DC maturation suppression and T-cell dysfunction while synergizing PTT with ferroptosis-induced ICD to amplify antitumor immunity (Fig. 14f). Pharmacokinetic analysis showed maximal tumor accumulation at 24 h post-injection through NIR-II/photoacoustic dual-signal peaking, with concurrent thermal imaging confirming rapid tumor temperature elevation to ~ 50 °C—achieving therapeutically effective hyperthermia thresholds (Fig. 14g). Comparative flow cytometry revealed GW4869-mediated exosome inhibition enhanced DC maturation by 1.72-fold versus PBS controls, while Fe^3+^-containing PF components exhibited 1.67-fold maturation enhancement, demonstrating complementary ferroptosis-mediated immunomodulation (Fig. 14h). The nanoplatform significantly downregulated PD-L1-associated exosomal marker CD63 (Fig. 14i) and elevated T-cell activation markers (Fig. 14j), establishing a coordinated immune response against B16F10 tumors through simultaneous exosomal PD-L1 blockade and ferroptosis-driven antigen presentation. Mechanistically, NRF2 has been identified as a transcriptional regulator of PD-L1. Pharmacological inhibition of NRF2 using ML385 reduces PD-L1 expression, thereby alleviating CTL suppression and enhancing antitumor immunity [37]. These multimodal approaches demonstrate the therapeutic potential of integrating ferroptosis induction with PD-L1 pathway modulation for enhanced cancer immunotherapy.

In conclusion, PD-1/PD-L1 pathway inhibition strategies show diverse progress in tumor immunotherapy, with varied inhibitors displaying distinct features in nanodelivery systems. Antibody inhibitors like nivolumab and pembrolizumab represent the most advanced clinical approach, featuring high specificity and extended half-life, but their use is limited by high costs and immune-related adverse effects [187]. Due to the current administration via standalone injection, their direct linkage to nanocarriers proves technically challenging. Nevertheless, considering nanocarriers' immense potential to address permeability and combination therapy issues, future material innovations may overcome these barriers. Small-molecule inhibitors, with efficient membrane penetration, oral bioavailability, and tunable chemistry, are highly suitable for nanocarrier delivery to extend half-life and minimize off-target toxicity. However, their relatively low specificity may lead to side effects, including unintended damage to immune cells in the TME [188]. Peptide inhibitors exhibit high tissue penetration, low immunogenicity, and low-cost synthesis, while their easy conjugation to nanosystems makes them ideal. Nanocarrier delivery addresses their enzymatic degradation vulnerability and short half-life, enhancing in vivo stability and enabling targeted release [189, 190]. Nucleic acid inhibitors achieve precision suppression via gene silencing or specific binding, but their poor uptake and instability restrict clinical use. Nanocarriers protect them from nucleases and boost intracellular delivery. Yet clinical translation remains hindered by off-target effects, immune barriers, and challenges in scalable production [191]. Despite hurdles, ongoing advances in nanocarrier design and combinatorial strategies will enable safer, more effective PD-1/PD-L1-targeted immunotherapies.

##### CTLA-4 Checkpoint Blockade

**Fig. 15:**
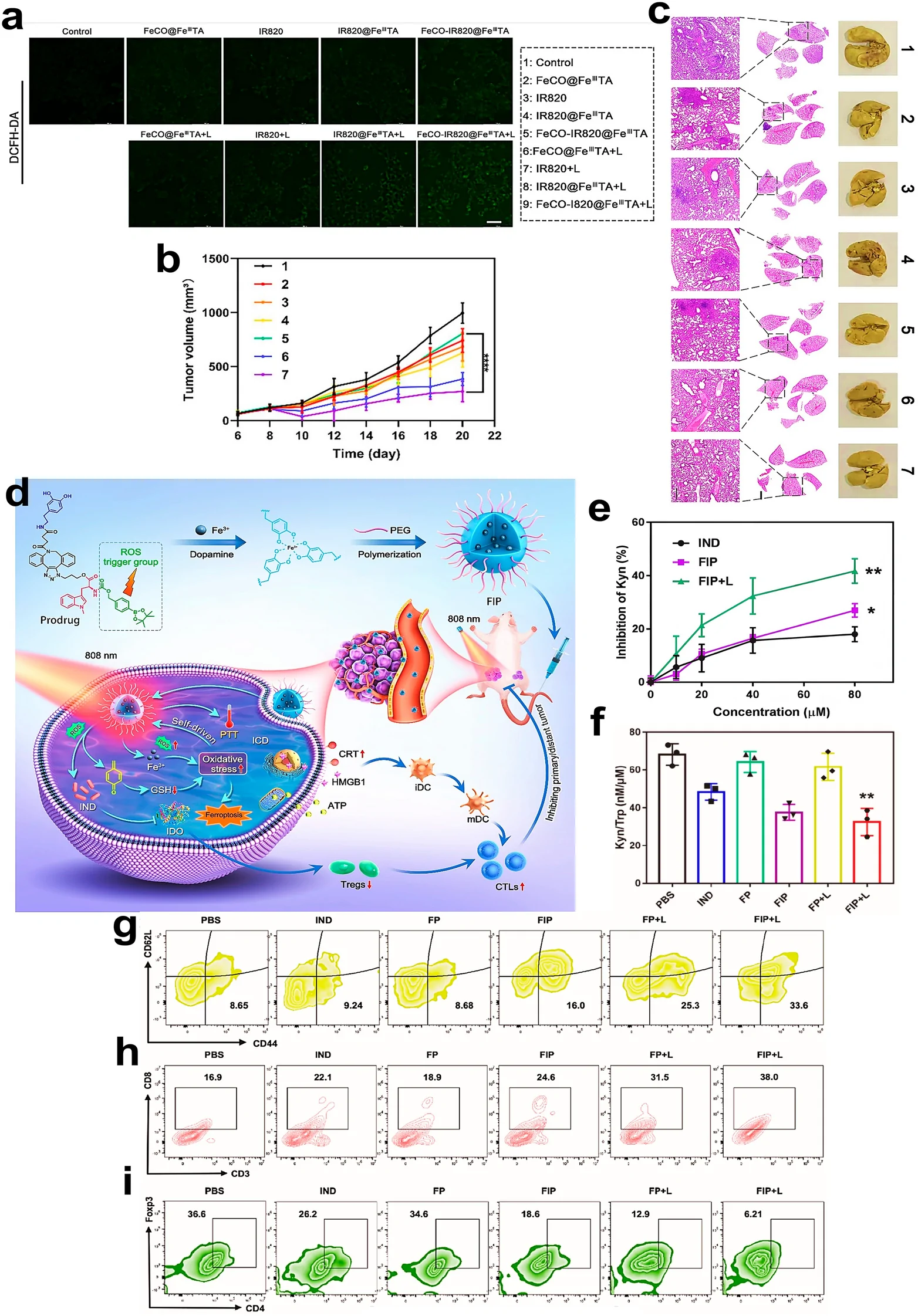
**a** Confocal microscopy of intracellular ROS levels using DCFH-DA probe across treatment groups. **b** Tumor growth kinetics in 4T1-bearing mice monitored every 2 days for 14 days. **c** Lung gross morphology and H&E-stained sections from seven experimental groups. Adapted with permission from [161], copyright 2022 Elsevier B.V. **d** FIP nanosystem design for combinatorial ferroptosis, photothermal therapy, and immune activation. **e** Kynurenine (Kyn) suppression rates in treated 4T1 cells. **f** Kyn/Trp ratio quantification by ELISA.** g** Splenic memory T cells (CD44^+^CD62L^+^) analyzed by flow cytometry post-treatment. Flow cytometric quantification of **h** tumor-infiltrating CD8^+^ T cells and **i** Tregs (CD4^+^Foxp^3+^). Adapted with permission from [166], copyright 2023 Elsevier Ltd

The interaction between CD28 on T cells and B7 molecules on DCs delivers a critical co-stimulatory signal for T cell activation. However, CTLA-4, expressed on T cells, competitively inhibits this process through its higher binding affinity for B7 compared to CD28, thereby suppressing T cell activation and serving as an immune checkpoint [192]. To counteract this immunosuppressive mechanism, anti-CTLA-4 antibodies have been employed to block inhibitory signaling and enhance T cell activation, offering a synergistic approach to improve ferroptosis-mediated immunotherapy. In a recent study, anti-CTLA-4 antibodies were integrated with multifunctional nanoparticles (FeCO-IR820@FeIIITA) composed of tannic acid, a CO prodrug (FeCO-PBA), Fe^3+^ ions, and the photosensitizer IR820 [161]. Under NIR irradiation, the FeCO-IR820@FeIIITA group exhibited the most intense green fluorescence, indicative of robust ROS generation driven by CO release, photothermal effects from IR820, and Fe^3+^-mediated ferroptosis (Fig. 15a). Elevated ROS levels subsequently induce tumor cell damage and ICD, promoting DC maturation and transforming immunologically “cold” tumors into immunoreactive microenvironments. In vivo studies demonstrated that FeCO-IR820@FeIIITA + L combined with CTLA-4 checkpoint blockade achieved a tumor growth inhibition rate of 72.9% (Fig. 15b), underscoring the therapeutic synergy between nanoparticle-enabled ferroptosis and immune checkpoint inhibition. Furthermore, the FeCO-IR820@FeIIITA + L + anti-CTLA-4 cohort showed a marked reduction in both size and quantity of lung metastatic nodules compared to control groups (Fig. 15c), validating the enhanced antimetastatic efficacy of this combinatorial strategy.

##### Metabolic Checkpoint: IDO Pathway Blockade

Indoleamine 2,3-dioxygenase (IDO) is frequently overexpressed in tumor cells, where it catalyzes the conversion of tryptophan (Trp)—an essential amino acid required for T-cell activation and proliferation—into kynurenine (Kyn). This enzymatic activity creates an immunosuppressive TME through dual mechanisms: Trp depletion-induced T-cell anergy and Kyn-mediated activation of Tregs and MDSCs via aryl hydrocarbon receptor signaling [193]. To counteract this immunosuppression, IDO inhibitors such as NLG919 and indoximod have been developed to restore Trp levels and block Kyn production, thereby enhancing ferroptosis–immunotherapy efficacy.

NLG919, a direct IDO enzymatic inhibitor, has been co-loaded with sorafenib onto ferritin-modified polymer carriers, forming a nanotherapeutic platform that induces ferritinophagy-dependent ferroptosis [113]. This approach promotes DC maturation, enhances tumor-infiltrating T-cell populations, and elevates cytokine secretion, effectively reprogramming the immunosuppressive TME. In contrast, indoximod (IND) exerts its IDO pathway inhibition through modulation of Kyn metabolism. Huang et al. developed a multifunctional nanoplatform (FIP) through Fe^3+^ chelation with phenolic groups and dopamine polymerization [166]. This system enables self-driven indoximod release while amplifying oxidative stress to synergistically enhance ferroptosis and photothermal-IDO immunotherapy (Fig. 15d). The FIP platform demonstrated significant IDO pathway blockade, evidenced by reduced Kyn levels (Fig. 15e) and decreased Kyn/Trp ratios (Fig. 15f). Remarkably, FIP + L treatment achieved complete primary tumor regression and suppressed pulmonary metastasis, indicating potent antimetastatic activity. Immunological profiling revealed expansion of memory T-cell populations from 8.65% to 33.6% (Fig. 15g), enhanced CTL activation (Fig. 15h), and significant Treg depletion (Fig. 15i), confirming successful TME immunoreprogramming through combined ferroptosis-PTT and IDO inhibition strategies.

##### CD47-SIRPα Checkpoint Inhibition

The CD47 protein, frequently overexpressed on malignant cell surfaces, engages with signal regulatory protein alpha (SIRPα) expressed by myeloid cells (particularly macrophages) to transmit an immunosuppressive “don't eat me” signal, enabling tumor immune evasion through phagocytosis inhibition [194]. To counteract this immune checkpoint mechanism, CD47 antagonists have been developed to disrupt CD47-SIRPα interactions, thereby potentiating macrophage-mediated tumor clearance and enhancing ferroptosis-based immunotherapy.

**Fig. 16:**
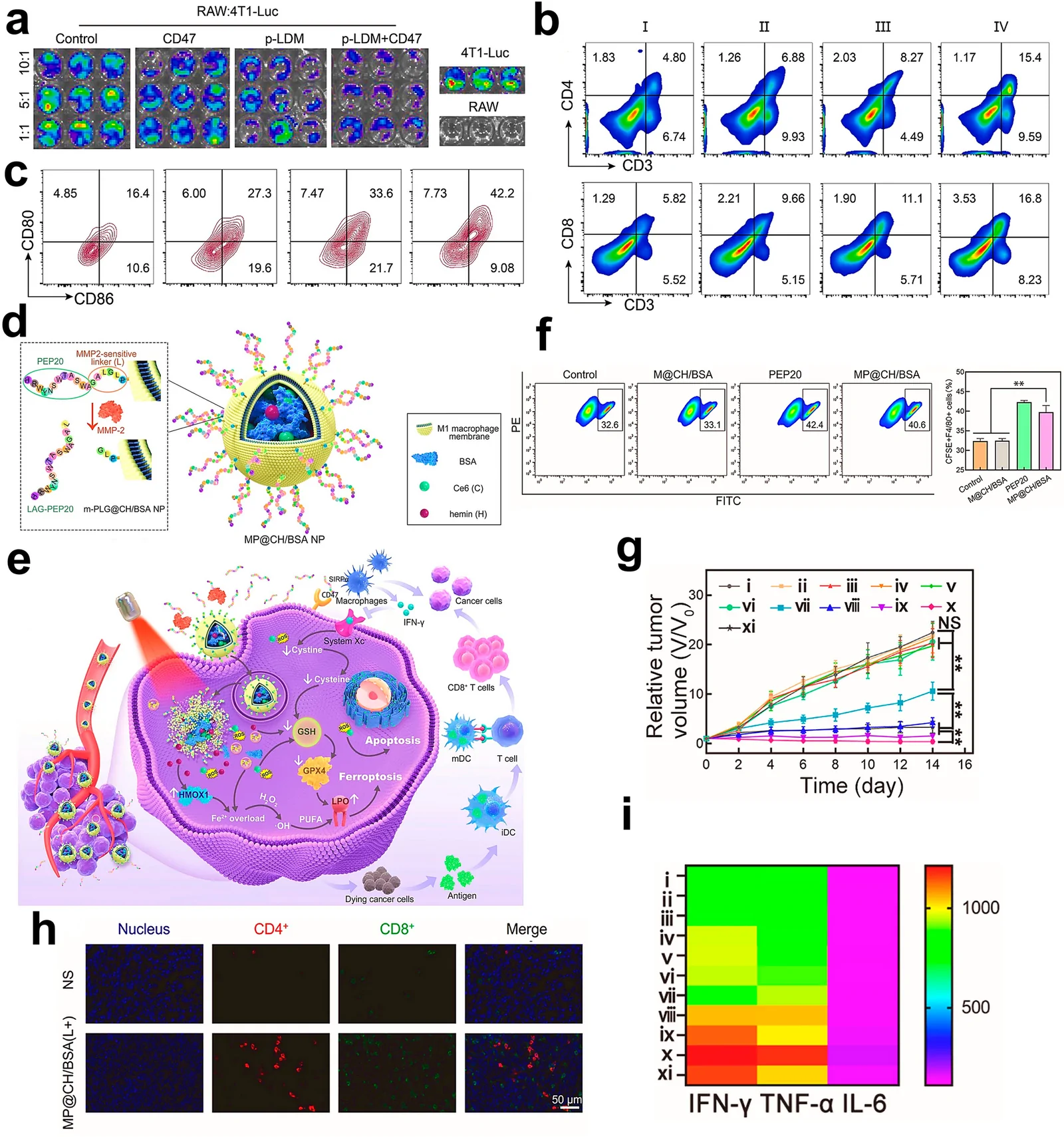
**a** Confocal microscopy imaging of RAW264.7 macrophage-mediated 4T1-Luc cell phagocytosis across treatment groups. **b** Flow cytometry analysis of tumor-infiltrating CD3^+^CD4^+^ T helper cells (Ths) and lymph node CD11c^+^CD80^+^CD86^+^ mature DCs. **c** Tumor-infiltrating CTLs (CD3^+^CD8^+^) quantified by flow cytometry. Adapted with permission from [39], copyright 2023 Elsevier B.V. **d, e** MMP-2-responsive MP@CH/BSA NP design and in vivo tumor-targeting mechanism. **f** Flow cytometric quantification of FITC^+^PE^+^ BMDM/4T1 co-cultures post-treatment. **g** 4T1 tumor growth curves under different therapeutic regimens. **h** Immunofluorescence co-localization of CD4^+^ (red) and CD8^+^ (green) T cells in tumor sections. **i** Heatmap of serum cytokine profiles (IFN-γ/TNF-α/IL-6) in treated 4T1-bearing mice. Adapted with permission from [150], copyright 2022 Elsevier Ltd

Recent studies describe an innovative self-amplifying ferroptosis-inducing metal–organic framework (Fe^3+^-H_2_BDC MOF) that synergistically initiates ferroptosis and ICD [39]. This dual-action system promotes emission of “find me” (e.g., ATP) and “eat me” (e.g., CRT) signals while concurrently administering anti-CD47 antibodies to neutralize immunosuppressive signaling, effectively reprogramming the TME. In vitro validation using luciferase-tagged 4T1 murine mammary carcinoma cells demonstrated significant bioluminescence reduction (*p* < 0.01) following anti-CD47 treatment, confirming enhanced macrophage phagocytic capacity (Fig. 16a). Combinatorial therapy with p-LDM and anti-CD47 antibodies elicited robust T-cell infiltration, yielding 2.82-fold and 3.22-fold increases in CD4^+^ and CD8^+^ T-cell populations respectively versus controls (Fig. 16b). Notably, the combination regimen surpassed p-LDM monotherapy in DC maturation (Fig. 16c), suggesting CD47 blockade amplifies antigen presentation efficiency.

To optimize therapeutic delivery, researchers engineered a novel nanoplatform integrating PEP20—a high-affinity CD47 inhibitory peptide—with MMP-2 responsive linkers [150]. This construct was grafted onto M1 macrophage membrane-coated nanoparticles co-loaded with Ce6 and hemin (MP@CH/BSA NPs), creating a multimodal system for simultaneous macrophage activation and T-cell stimulation (Fig. 16d, e). Comparative analysis revealed PEP20-containing MP@CH/BSA (L +) significantly outperformed M@CH/BSA controls in macrophage recruitment (Fig. 16f) and tumor suppression metrics, achieving maximal inhibition of primary tumor growth and pulmonary metastasis. Therapeutic efficacy proved dependent on both PEP20-mediated CD47 blockade and ferroptosis induction, as evidenced by diminished tumor control in PEP20-deficient (ix group) and deferoxamine-treated (xi group) cohorts (Fig. 16g). Immunophenotyping revealed enhanced CD4^+^/CD8^+^ T-cell infiltration (Fig. 16h) and elevated Th1-associated cytokine levels (IFN-γ, TNF-α, IL-6) in MP@CH/BSA (L +)-treated tumors (Fig. 16i), confirming successful immune microenvironment remodeling. These findings collectively demonstrate that coordinated ferroptosis induction and CD47-SIRPα axis disruption creates a self-reinforcing antitumor immune cycle through enhanced phagocytosis, DC maturation, and cytotoxic T-cell activation.

##### B7-H3 Pathway Inhibition

B7-H3 (CD276), a transmembrane protein identified in 2001 as a member of the B7 superfamily, demonstrates tumor-restricted overexpression across multiple malignancies. This immunomodulatory molecule suppresses T cell activation while maintaining minimal expression in normal tissues. Unlike conventional monoclonal antibodies, bispecific antibodies (BiAbs) employ engineered architectures combining two distinct single-chain variable fragments. Notably, bispecific T cell engagers simultaneously engage CD3ε on T cells and TAAs, facilitating immune synapse formation between effector T cells and tumor cells. This interaction triggers T cell activation, inflammatory cytokine release, and subsequent tumor cell lysis.

**Fig. 17:**
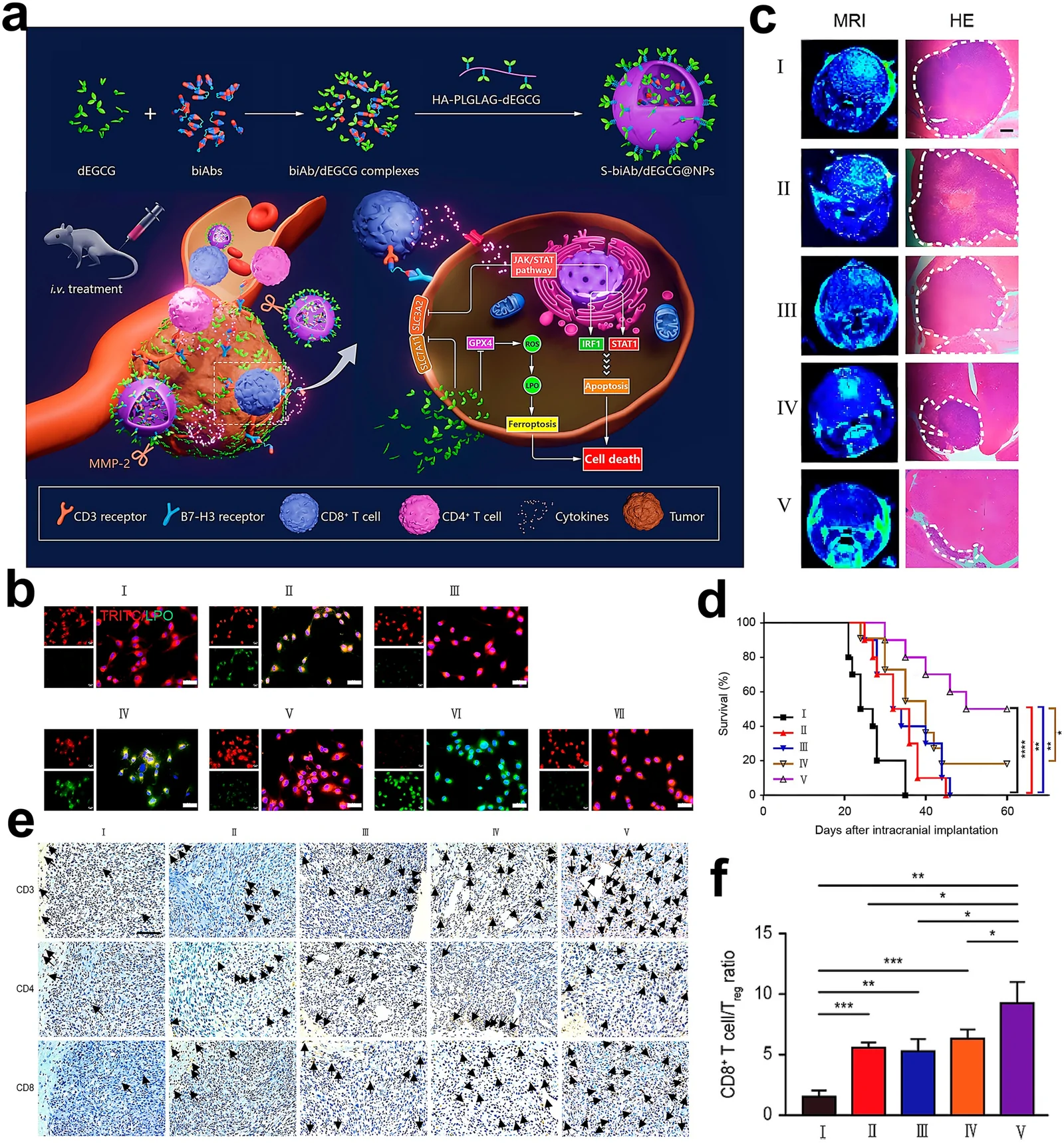
**a** Schematic of S-biAb/dEGCG@NP-mediated ferroptosis-potentiated glioblastoma (GBM) immunotherapy. **b** Confocal imaging of lipid peroxidation (C11-BODIPY staining) in U87 cells across treatments. **c** Post-treatment (day 35) MRI and H&E-stained brain sections from orthotopic GBM models. **d** Survival curves of GBM-bearing mice (*n* = 8). **e** Flow cytometric quantification of brain-infiltrating CD3^+^/CD4^+^/CD8^+^ T cells. **f** CD8^+^ T cell/Treg ratio in tumor-infiltrating lymphocytes (TILs) by flow cytometry. Adapted with permission from [51], copyright 2023 American Chemical Society

Capitalizing on this mechanism, Fan et al. developed MMP-2-responsive nanoparticles (S-biAb/dEGCG@NPs) integrating anti-B7-H3 × CD3 BiAbs, a dimer of epigallocatechin gallate (dEGCG), and hyaluronic acid-PLGLAG-dEGCG conjugates to potentiate immune tolerance reversal and ferroptosis in glioblastoma (Fig. 17a) [51]. In vitro lipid peroxidation assays revealed that U87 cells treated with dEGCG plus IFN-γ exhibited enhanced LPO signals versus controls (Fig. 17b). This pro-ferroptotic effect was abrogated by co-incubation with ferrostatin-1 (Fer-1), confirming that dEGCG synergizes with ICB-mediated IFN-γ secretion to amplify ferroptosis. MRI volumetry and histopathological analysis demonstrated significant tumor regression in the S-biAb/dEGCG@NP cohort (Fig. 17c), with concomitant survival benefit (Fig. 17d), validating MMP-2-sensitive nanoparticle efficacy against GBM. Immunophenotyping revealed robust CD3^+^ and CD8^+^ T cell infiltration in S-biAb/dEGCG@NP-treated tumors (Fig. 17e), accompanied by a 5.9-fold elevation in CD8^+^/Treg ratio versus controls (Fig. 17f). These findings collectively demonstrate the dual capacity of these nanoparticles to overcome tumor-mediated immunosuppression and reactivate cytotoxic T cell responses.

#### Precision Modulation of Immunosuppressive Cell Populations

Immunosuppressive cells critically regulate immune homeostasis by attenuating excessive immune activation, thereby preventing autoimmune pathologies. However, tumor-associated immunosuppressive populations—including TAMs, Tregs, MDSCs, and tumor-associated neutrophils (TANs)—paradoxically foster tumor progression by facilitating metastasis, angiogenesis, and immune evasion through cytokine-mediated signaling [195]. Consequently, therapeutic modulation of these immunosuppressive cell populations to overcome tumor immune resistance represents a promising strategy for augmenting ferroptosis-based immunotherapy efficacy.

**Fig. 18:**
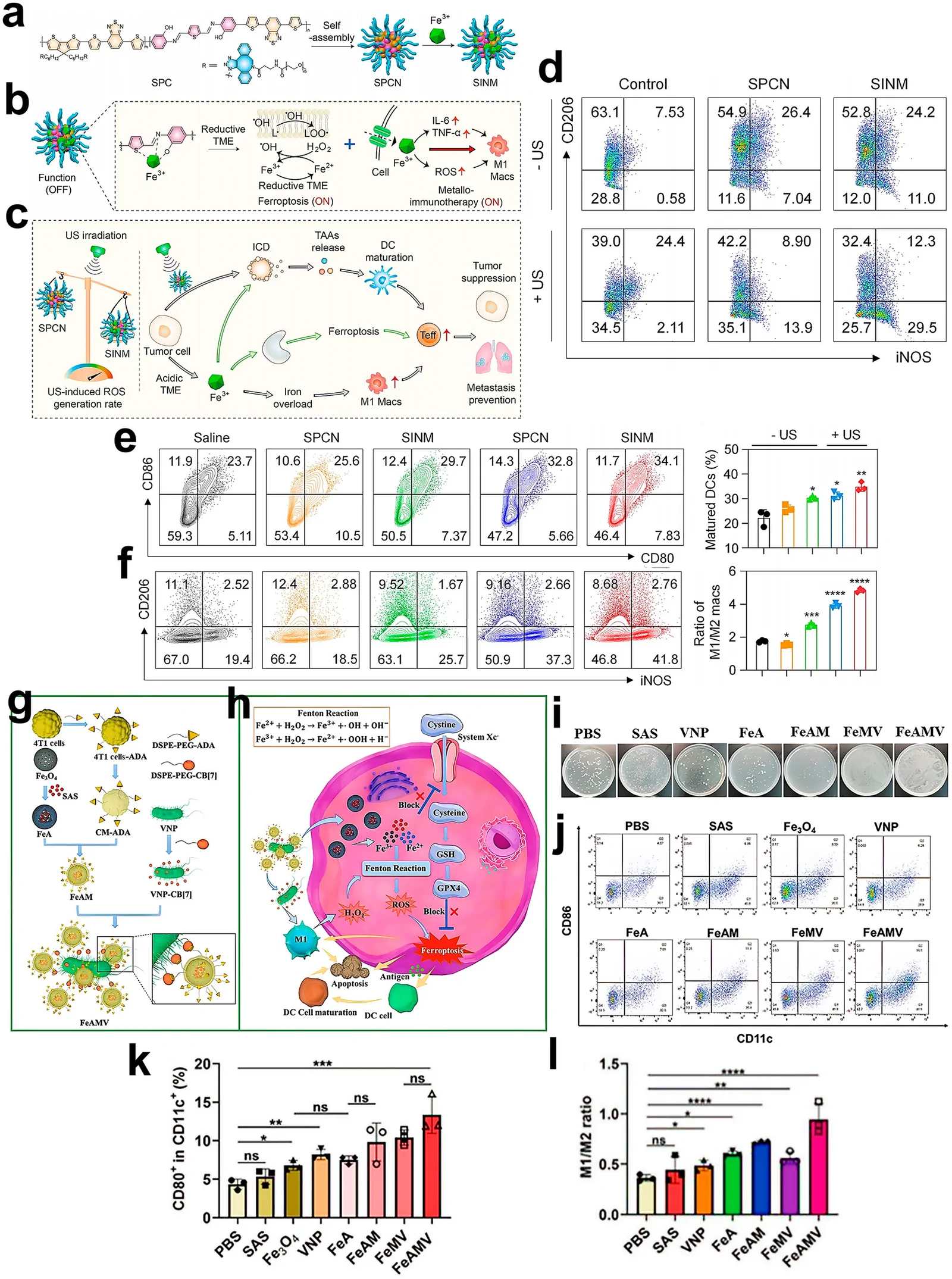
**a** Synthesis scheme and chemical structure of SINM. **b** Iron-mediated ferroptosis and metallo-immunotherapy mechanisms in reductive TME. **c** Spatiotemporally controlled SINM enables precision sono-metallo-immunotherapy in vivo. **d** BMDM polarization analysis (M1/M2) post 12 h SINM/SPCN incubation + 60 s sonication. **e** M1 (iNOS^+^)/M2 (CD206^+^) macrophage ratio in TAMs (F4/80^+^). **f** DCs maturation (CD80^+/^CD86^+^) in TDLNs at day 10. Adapted with permission from [43], copyright 2023 Wiley–VCH GmbH, Weinheim. **g** Synthesis of Fe_3_O_4_@CM-ADA/CB-VNP via host–guest conjugation. **h** FeAM tumor-targeting strategy combining ferroptosis induction and antitumor immunity activation. **i** Representative tumor morphology across treatment groups post-therapy. **j, k** DCs maturation (CD86^+^/CD11c^+^) quantification in tumor tissues after 24 h treatment. **l** M1 (CD11c^+^)/M2 (CD206^+^) macrophage ratio in tumor tissues. Adapted with permission from [124], copyright 2023 Wiley–VCH GmbH, Weinheim

Within the TME, TAMs predominantly exhibit an M2-polarized phenotype that promotes tumorigenesis. These cells enhance immune evasion through multiple mechanisms: (1) expression of immune checkpoint ligands (e.g., PD-L1), (2) secretion of anti-inflammatory cytokines (IL-10), and (3) VEGF-mediated angiogenesis [196]. Reprogramming M2 TAMs toward tumoricidal M1 macrophages emerges as a potential therapeutic approach to counteract immunosuppression and potentiate ferroptosis–immunotherapy. Notably, iron overload has been demonstrated to induce M1 polarization via interferon regulatory factor 5 pathway activation and arginase-1 suppression, thereby shifting the M1/M2 balance [197]. Capitalizing on this iron-mediated polarization mechanism, He et al. engineered a semiconductor polymer-based nano-immunomodulator (SINM) featuring Fe^3+^-chelating scaffolds and PEGylated side chains (Fig. 18a) [43]. Upon TME accumulation, Fe^2+^ liberated from SINM catalyzes ultrasound-enhanced Fenton reactions, generating ROS to drive tumor ferroptosis and ICD. Concurrent iron overload promotes M1 macrophage repolarization, achieving dual suppression of primary tumor growth and pulmonary metastases (Fig. 18b, c). Flow cytometry revealed SINM-induced M1 populations exceeding SPCN (iron-free) controls by 1.6-fold, with further enhancement under ultrasound exposure (Fig. 18d), confirming synergistic iron/sonodynamic polarization effects. Therapeutic evaluation demonstrated superior tumor control in SINM + US cohorts (91.2% primary tumor inhibition; 93.2% distant tumor suppression), outperforming SPCN by 1.7-fold and 1.3-fold respectively. Enhanced systemic immunity was evidenced by elevated CD80^+^CD86^+^ mature DC populations in tumor-draining lymph nodes (Fig. 18e) and a maximal M1/M2 ratio (41.8% iNOS^+^CD206^−^ cells) within SINM + US tumors (Fig. 18f), indicating durable tumor immune microenvironment (TIME) remodeling. Complementary studies by SAS/Fe_3_O_4_ investigators demonstrated synergistic ferroptosis–immunotherapy using tumor membrane-coated Fe_3_O_4_ nanoparticles (FeAMV) combined with live bacteria (Fig. 18g) [124]. Fe_3_O_4_/SAS co-delivery potentiated M1 polarization while bacterial colonization amplified intratumoral H_2_O_2_ levels, enhancing both ferroptosis and immune activation (Fig. 18h). Tumor-specific accumulation of FeAMV components was validated through elemental analysis (Fig. 18i). FeAMV treatment elicited superior DC maturation (CD11c^+^CD86^+^ cells) versus monotherapies (Fig. 18j, k), achieving maximal tumor growth inhibition and M1/M2 polarization (Fig. 18l), confirming combinatorial ferroptosis–bacterial immunotherapy efficacy.

**Fig. 19:**
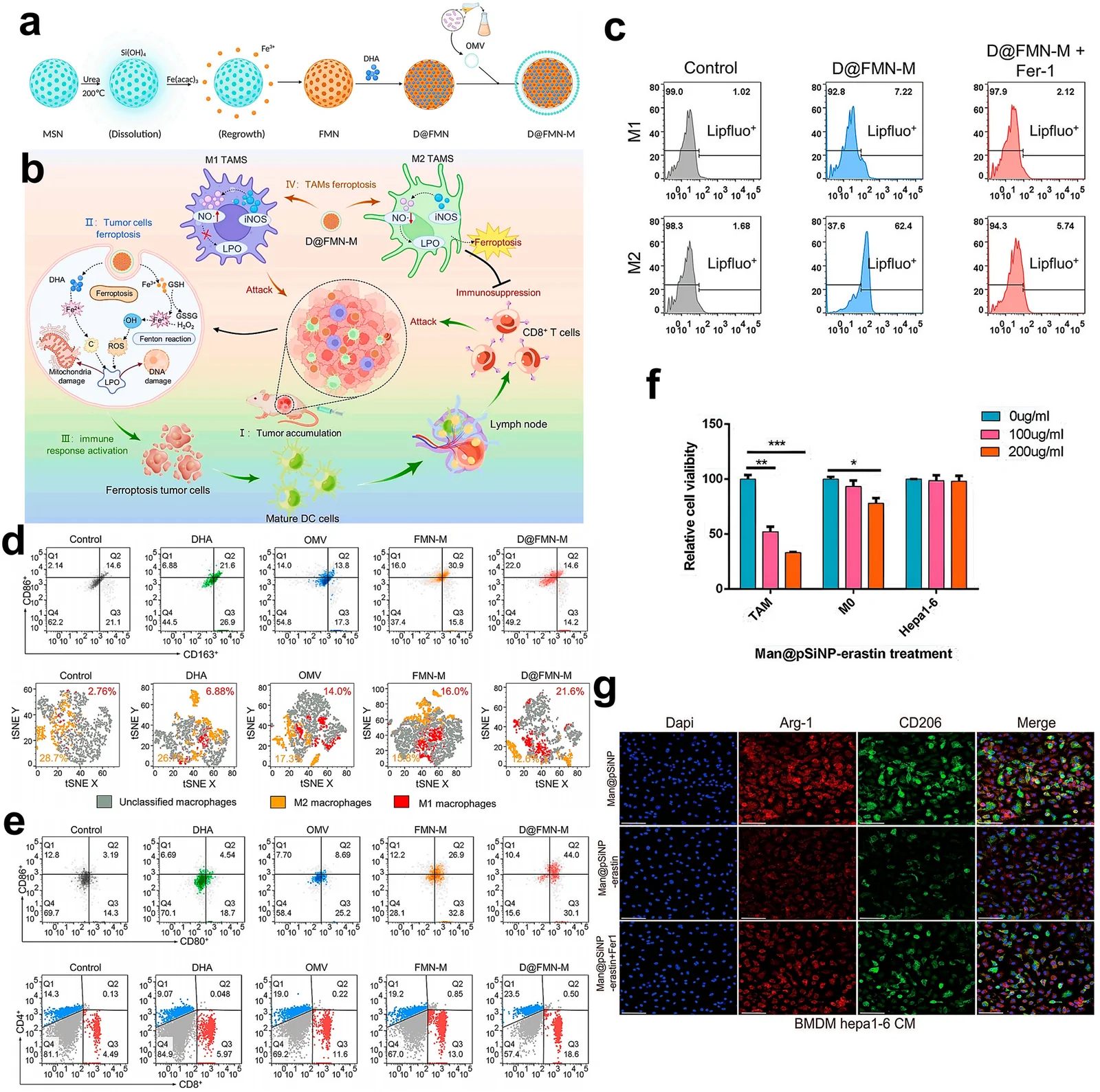
**a** Assembly scheme of D@FMN-M. **b** D@FMN-M-mediated dual ferroptosis induction in tumor cells/M2-TAMs enhances antitumor immunity. **c** Lipid peroxidation (Lipfluo^+^) quantification by flow cytometry across treatments. **d** Macrophage polarization analysis (M1/M2) with t-SNE mapping of CD163^+^ M2-TAMs in tumors. **e** DCs maturation (CD80^+^/CD86^+)^ and CD4^+^/CD8^+^ T cell profiling in 4T1 tumor models. Adapted with permission from [80], copyright 2023 Elsevier Ltd. **f** Cell viability of TAMs/M0 macrophages/Hepa1-6 cells post-Man@pSiNPs-erastin treatment. **g** Immunofluorescence staining of TAM subsets (Arg-1^+^/CD206^+^) infiltration under different therapies. Adapted with permission from [148], copyright 2023 Wiley–VCH GmbH, Weinheim

The distinct metabolic profile of M2 macrophages, particularly their reduced inducible nitric oxide synthase (iNOS)/NO• expression compared to M1 counterparts, presents a therapeutic opportunity. Mechanistically, iNOS catalyzes arginine conversion to NO•, which suppresses 15-lipoxygenase-mediated oxidative reactions, thereby limiting LPO accumulation and conferring ferroptosis resistance in M2 macrophages [19]. To exploit this vulnerability, a biomimetic ferroptosis inducer (D@FMN-M) was engineered through encapsulation of DHA and iron-doped MSNs, surface-modified with outer membrane vesicle (Fig. 19a) [80]. Upon cellular internalization, Fe^3+^ undergoes GSH-mediated reduction to Fe^2+^, initiating Fenton reactions that cleave DHA's peroxide bridges. This process generates ·OH, driving intracellular LPO accumulation and triggering ferroptosis in both malignant cells and M2-polarized TAMs (Fig. 19b). Lipid peroxidation analysis using Lipifluor™ revealed pronounced LPO fluorescence in D@FMN-M-treated M2 macrophages, contrasting with minimal signal in M1 populations (Fig. 19c), confirming preserved antioxidant capacity in M1 cells. Immunophenotyping demonstrated significant CD163 downregulation (M2 marker) post-treatment (Fig. 19d), indicating successful M2-TAM depletion and favorable M1/M2 ratio modulation. These changes correlated with enhanced DC maturation and elevated CD8^+^ T cell infiltration compared to control treatments (Fig. 19e), confirming improved ICD and antitumor immunity.

Previous studies identified xCT (system Xc^−^ catalytic subunit) as a critical regulator of M2 polarization through SOCS3-STAT6-PPAR-γ signaling while concurrently suppressing ferroptosis via GPX4/RRM2 pathways. To validate this axis, mannose-functionalized porous silica nanoparticles loaded with erastin (Man@pSiNPs-erastin) were developed for M2-TAM-specific xCT knockout [148]. Xenograft models demonstrated significantly reduced tumor volumes, diminished Ki67 proliferation indices, and attenuated tumor vascularization in xCT KO (xCT knockout) versus wild-type mice. Dose–response assays revealed selective cytotoxicity against TAMs and M0 precursors without compromising Hepa1-6 viability (Fig. 19f), confirming ferroptosis-mediated specificity. Immunofluorescence analysis further showed Man@pSiNPs-erastin-induced suppression of M2 polarization, reversible by ferroptosis inhibitor Fer-1 (Fig. 19g), establishing xCT ablation as a dual-acting mechanism for M2-TAM eradication.

**Fig. 20:**
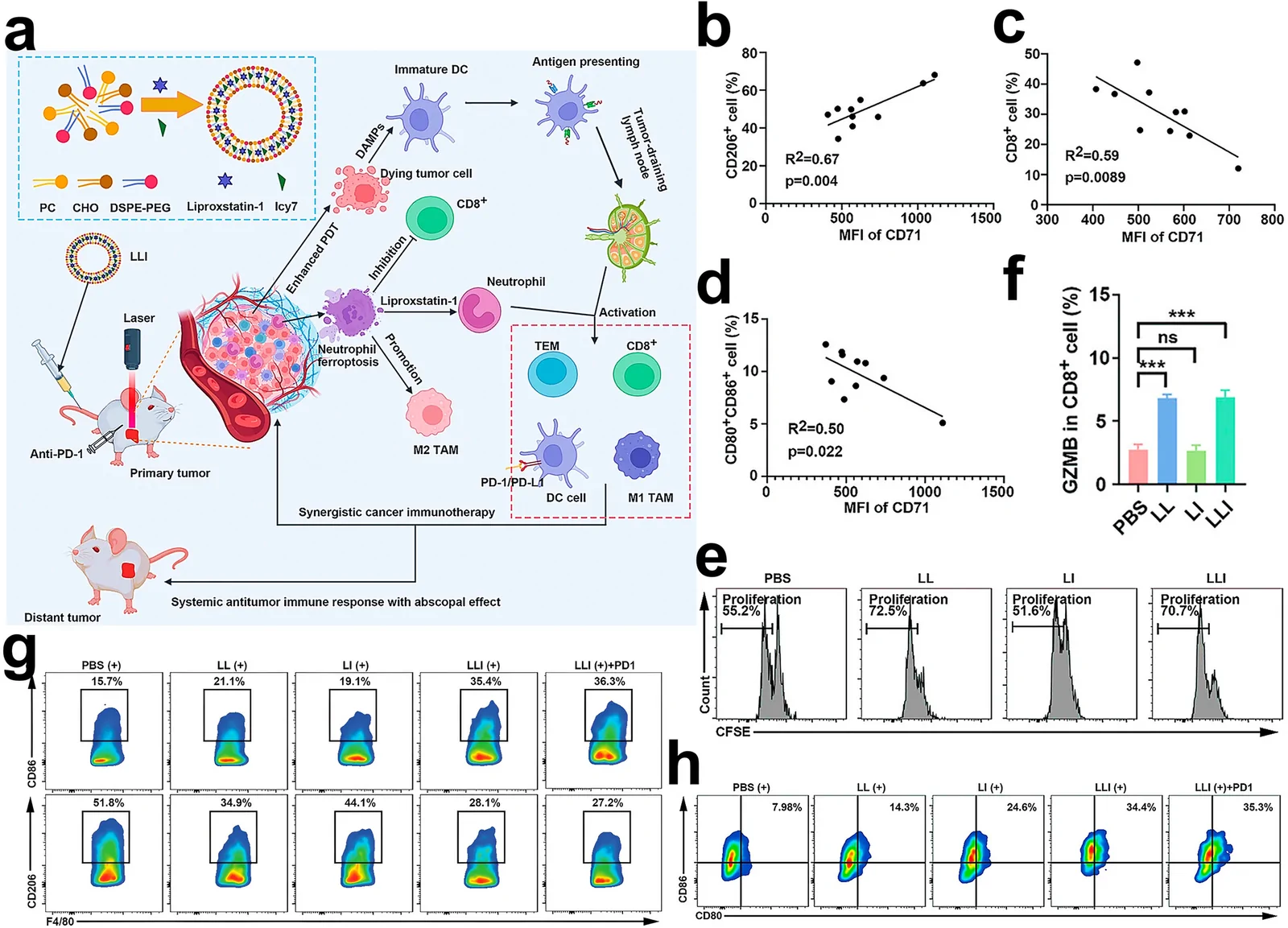
**a** LLI nanodrug enabling immunogenic PDT/neutrophil-targeted immunotherapy to potentiate anti-PD-1® efficacy with abscopal immunity. **b** Correlations between CD71 MFI and M2-TAM infiltration, **c** CD8^+^ T cell abundance, and **d** DC maturation in tumors. **e** GzmB^+^CD8^+^ T cells quantified via flow cytometry post-co-culture with LLI-primed neutrophils. **f** CD8^+^ T cell proliferation (CFSE dilution) after co-culture with LLI-activated neutrophils. **g** M1/M2-TAM ratio in tumors quantified by flow cytometry across treatment groups. **h** DCs maturation (CD80^+^/CD86^+^) in tumors analyzed by flow cytometry. Adapted with permission from [167], copyright 2024 Wiley–VCH GmbH, Weinheim

Emerging evidence indicates that TANs spontaneously undergo ferroptosis within the TME, where released LPO impairs T cell functionality and promote tumor progression [198]. Targeting neutrophil ferroptosis presents a promising therapeutic strategy to alleviate tumor immune suppression. In this context, researchers developed dual-functional liposomes (LLI) co-loaded with the ICD inducer Icy7 and the ferroptosis inhibitor Liproxstatin-1, which simultaneously induce ICD in tumor cells and inhibit neutrophil ferroptosis, thereby reprogramming the immunosuppressive TME and establishing durable antitumor immune memory (Fig. 20a) [167]. Immunofluorescence analysis demonstrated a positive correlation between ferroptotic neutrophils and M2 macrophage infiltration (Fig. 20b), while revealing inverse correlations with CD8^+^ T cell density (Fig. 20c) and DC maturation status (Fig. 20d). These findings suggest that neutrophil ferroptosis contributes to an immunosuppressive TME. Functional validation showed that neutrophils treated with PBS or LI (Liproxstatin-1-free formulation) significantly suppressed CD8^+^ T cell proliferation (Fig. 20e). Conversely, Liproxstatin-1 treatment markedly enhanced IFN-γ production and Granzyme B expression in CD8^+^ T cells (Fig. 20f), confirming that ferroptosis inhibition restores T cell effector functions. Notably, the LLI (+) treatment group exhibited substantial M1 macrophage polarization compared to control groups (Fig. 20g), accompanied by enhanced DC maturation (Fig. 20h). When combined with PD-1 checkpoint blockade, LLI synergistically amplified antitumor immunity through dual mechanisms: reversing immunosuppressive networks in the TME and potentiating systemic immune activation. This combinatorial approach significantly suppressed primary tumor growth and metastatic dissemination, demonstrating enhanced therapeutic efficacy compared to monotherapies.

### Biophysical Hallmark Remodeling in the Tumor Microenvironment

The distinct biophysical features of tumor tissues, particularly hypoxia and abnormally dense ECM composition, create formidable barriers that impede immune effector cell infiltration and compromise antitumor immunity. This pathophysiological characteristic of the TME necessitates strategic intervention to overcome immune exclusion. Emerging evidence suggests that functionalized nanoplatforms combining ferroptosis induction with immunotherapeutic strategies may offer a promising solution by physically remodeling TME properties. Targeted modulation of these critical biophysical barriers could enhance immune cell trafficking into tumor beds while restoring effector functions, ultimately enabling effective tumor eradication.

#### Modulating Intratumoral Hypoxia

Hypoxia, a hallmark feature of TME in numerous malignancies, arises from compromised vascular oxygenation coupled with heightened oxygen demands from rapidly proliferating neoplastic cells. This oxygen-deprived state triggers activation of hypoxia-inducible factor (HIF) and its downstream signaling cascades, driving tumor invasiveness, progression, and metastatic potential while simultaneously fostering immune evasion through multiple mechanisms. HIF-mediated pathways stimulate production of immunosuppressive cytokines that promote M2-polarization of TAMs and enhance MDSC functionality [199]. Notably, HIF-1α stabilization enables hypoxic tumor cells to resist ferroptosis through enhanced fatty acid metabolism and lipid storage, mitigating peroxide-induced membrane damage [200].

**Fig. 21:**
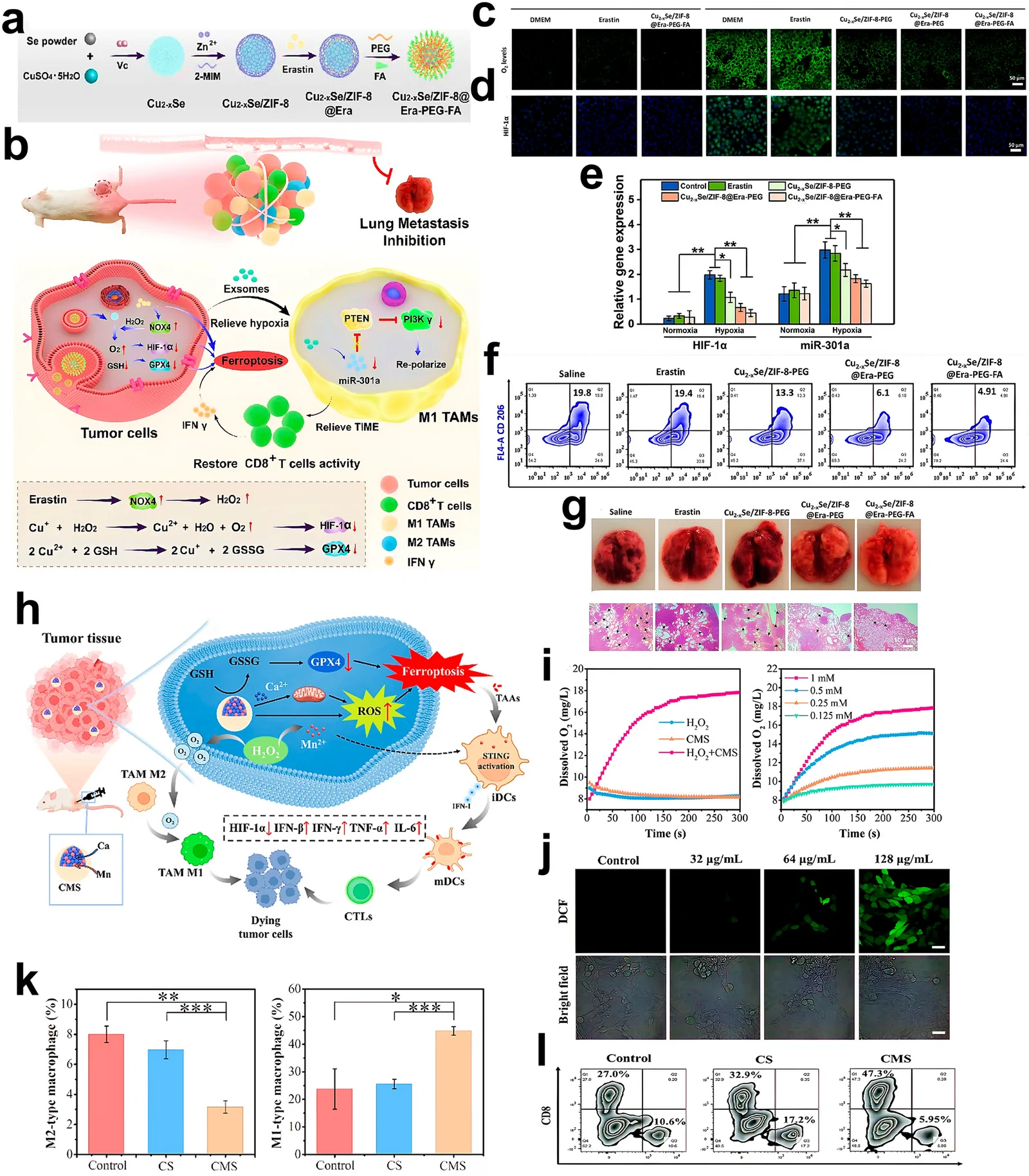
**a** Synthesis scheme of Cu_2−x_Se/ZIF-8@Era-PEG-FA. **b** Cu_2−x_Se/ZIF-8@Era-PEG-FA enables H_2_O_2_ → O_2_ conversion and GSH depletion to drive ferroptosis, repolarize TAMs, and activate antitumor immunity. **c** Intracellular O_2_ fluorescence imaging (Ir(III) probe) across treatments. **d** Immunofluorescence (IF) analysis of HIF-1α in tumor sections. **e** qRT-PCR quantification of exosomal PD-L1 mRNA levels. **f** M2-TAMs (CD206^+^) infiltration in tumors. **g** Lung metastatic foci bioluminescence imaging in 4T1 tumor models. Adapted with permission from [33], copyright 2023 American Chemical Society. **h** CMS nanoplatform triggers ferroptosis while reprogramming TAMs/DCs for enhanced antitumor immunity. **i** Time-dependent dissolved O_2_ kinetics in H_2_O_2_/CMS systems (left); CMS + H_2_O_2_ under varying H_2_O_2_ concentrations (right). **j** Confocal imaging of ROS (DCFH-DA) in CMS-treated 4T1 cells. **k** M1 (iNOS^+^)/M2 (CD206^+^) macrophage ratio in tumors by flow cytometry. **l** CD8^+^ T cell infiltration analysis by flow cytometry. Adapted with permission from [69], copyright 2024 KeAi Communications Co. Ltd

Emerging evidence reveals that tumor-derived exosomes containing miR-301a can activate PTEN/PI3Kγ signaling in TAMs, further skewing their differentiation toward pro-tumorigenic M2 phenotypes. In a therapeutic approach targeting these mechanisms, Li et al. engineered a multifunctional nanoparticle system (Cu_2−x_Se/ZIF-8@Era-PEG-FA) featuring a copper selenide core, zeolitic imidazolate framework-8 (ZIF-8) shell, and erastin payload (Fig. 21a) [33]. This platform demonstrates dual oxygen-generating and GSH-depleting capabilities through copper valence cycling, effectively alleviating tumor hypoxia (Fig. 21b). Confocal microscopy revealed significantly attenuated hypoxia-probe fluorescence in nanoparticle-treated cells compared to erastin-only or DMEM controls (Fig. 21c), corroborating the system's catalytic H_2_O_2_ → O_2_ conversion capacity. Western blot analysis confirmed concomitant HIF-1α downregulation (Fig. 21d), while exosomal miR-301a expression decreased by 60% post-treatment (Fig. 21e). These changes translated to reduced M2-TAM infiltration (Fig. 21f) and diminished pulmonary metastasis in 4T1 tumor models (Fig. 21g), with significantly increased splenic effector memory T cell populations, further demonstrating that hypoxia-alleviating strategies can potentiate host antitumor immunity.

In a parallel investigation, researchers developed a calcium-manganese dual-ion hybrid nanostimulator (CMS) that synergistically induces ferroptosis through GSH depletion and ROS accumulation [69]. Concurrently, CMS catalyzes H_2_O_2_ decomposition to produce oxygen, effectively ameliorating tumor hypoxia and activating innate immunity. This dual mechanism drives HIF-1α downregulation and promotes TAM repolarization from immunosuppressive M2 to antitumor M1 phenotypes (Fig. 21h). Experimental validation demonstrated that CMS addition to H_2_O_2_ solutions elicited a concentration-dependent elevation in dissolved oxygen levels (Fig. 21i), confirming its catalytic capacity for oxygen generation. ROS production was quantitatively verified through CLSM analysis using DCFH-DA fluorescent probes (Fig. 21j). Immunophenotyping revealed a 2.5-fold reduction in M2 macrophage infiltration and 1.9-fold increase in M1 polarization within CMS-treated tumors compared to controls (Fig. 21k), substantiating the oxygen-mediated immunomodulatory effects. The combined ferroptosis induction and immune activation significantly enhanced DC maturation, CTL infiltration (Fig. 21l), and pro-inflammatory cytokine secretion, collectively demonstrating CMS efficacy in reprogramming the immunosuppressive TME.

#### Precision Modulation of the Extracellular Matrix

**Fig. 22:**
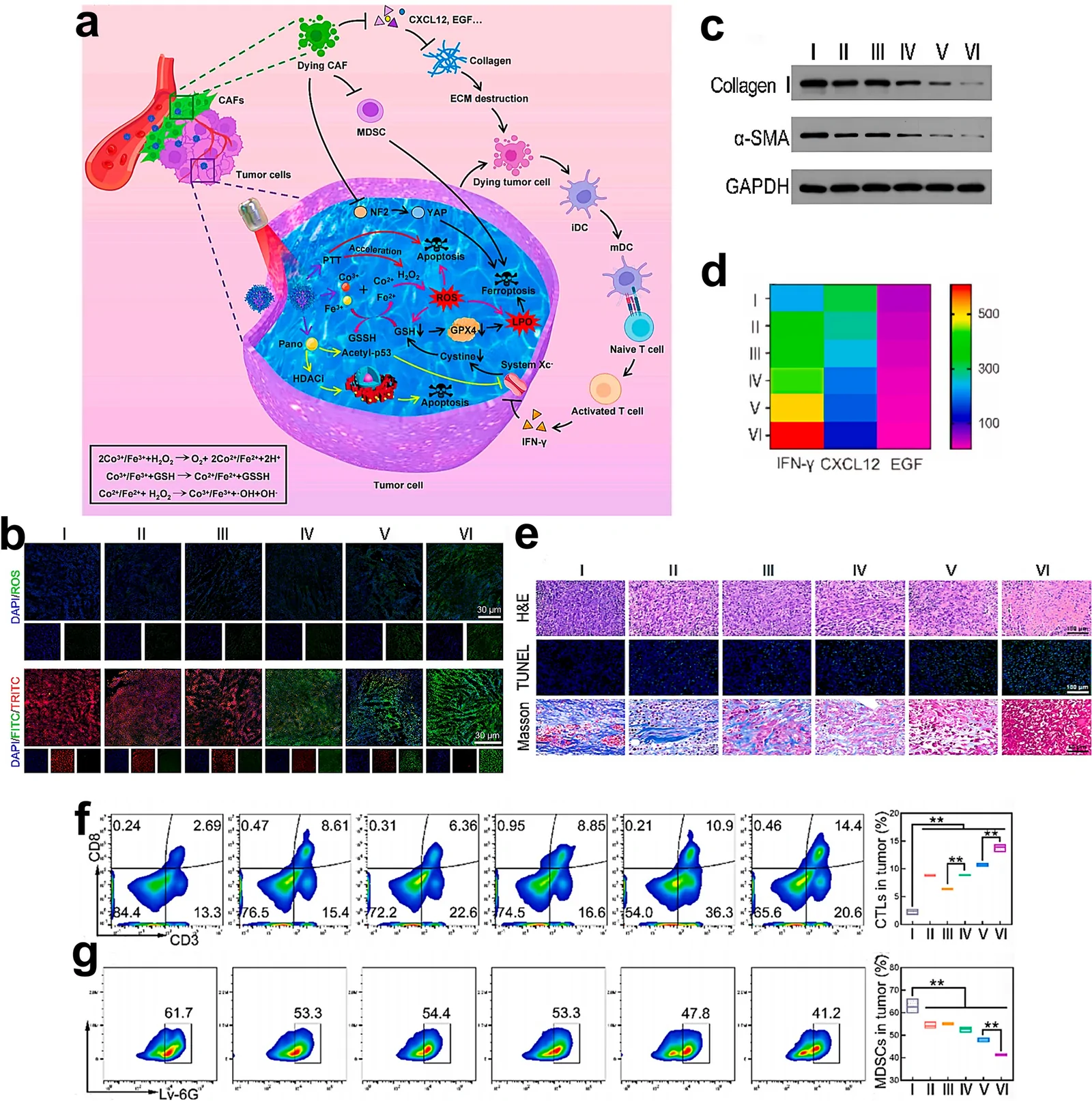
**a** T-FeCo/P-mediated ferroptosis activation and ECM remodeling synergistically enhance antitumor immunity. **b** Confocal imaging of ROS (DCFH-DA, green) and LPO (C11-BODIPY, red) in 4T1/CAF tumors across treatments. **c** Western blot analysis of ECM markers (Collagen I/α-SMA) expression post-treatment. **d** Heatmap profiling IFN-γ/CXCL12/EGF levels in 4T1/CAF models (mean ± SD, *n* = 3). **e** H&E (necrosis), TUNEL (apoptosis), and Masson's trichrome (fibrosis) staining of tumor sections (scale bar: 50 μm). **f** Tumor-infiltrating CTLs (CD3^+^CD8^+^) quantified by flow cytometry. **g** MDSCs (CD11b^+^Ly-6G^+^) infiltration analysis via flow cytometry. Adapted with permission from [121], copyright 2024 Elsevier B.V

The dysregulated ECM, a critical TME component, exhibits characteristic pathological alterations in malignancies. Tumor-associated ECM undergoes significant densification through excessive collagen crosslinking and overproduction of glycoproteins/glycosaminoglycans [201]. This structural remodeling demonstrates dual oncogenic effects: Enhanced matrix stiffness facilitates tumor invasion and metastasis, while simultaneously impeding therapeutic drug penetration and restricting antitumor immune cell infiltration, ultimately compromising treatment efficacy [202]. Emerging research highlights ECM remodeling as a promising therapeutic target, particularly given collagen's predominant role in ECM composition. Strategic collagen degradation could potentially inhibit metastasis, improve nanocarrier-mediated drug delivery, and enhance immune effector cell infiltration, thereby potentiating ferroptosis-based immunotherapy. Capitalizing on this rationale, the T-FeCo/P nanocarriers—telmisartan-PEG2000-modified Fe/Co bimetallic MOFs encapsulating the histone deacetylase (HDAC) inhibitor panobinostat were developed [121]. This dual-targeting system effectively engaged both 4T1 cells and CAFs, inducing ferroptosis while mediating ECM disruption through collagen degradation. The remodeled TME enhanced CTL infiltration and drug penetration while modulating the NF2/Hippo-YAP pathway and reducing MDSC recruitment, collectively sensitizing tumors to ferroptosis (Fig. 22a). Mechanistic evaluation revealed that T-FeCo/P + L (laser) treatment generated significantly elevated ROS and LPO levels in both cell types (Fig. 22b), confirming potent ferroptosis induction. Concurrent CAF suppression was evidenced by diminished α-SMA expression (Fig. 22c) and reduced epidermal growth factor (EGF) secretion (Fig. 22d). ECM structural disruption was confirmed through decreased type I collagen deposition (Fig. 22c) and attenuated blue signals from collagen fibers via Masson's trichrome staining (Fig. 22e). Subsequent TME remodeling manifested as CXCL12 chemokine reduction (Fig. 22d), enhanced CD8^+^ T cell infiltration (Fig. 22f), and decreased MDSC accumulation (Fig. 22g), demonstrating improved immune cell trafficking and ferroptosis therapeutic efficacy through ECM normalization.

Notably, ferroptosis may produce dual effects on antitumor immune responses: it effectively kills tumor cells and activates antitumor immunity, but may impair key immune effector cells, thus compromising antitumor responses. Studies have indicated that CD8^+^ T cells exhibit greater ferroptosis sensitivity than tumor cells, especially under GPX4 inhibition. Furthermore, blocking ACSL4-mediated ferroptosis can attenuate T-cell antitumor activity, limiting the application of certain ferroptosis-inducing strategies. Intriguingly, however, when induced via SLC7A11 inhibition or AA supplementation, ferroptosis may preserve or even enhance CD8^+^ T-cell antitumor function, suggesting that different ferroptosis-inducing approaches display distinct effects on immune cells [203]. Furthermore, elevated CD36 expression promotes fatty acid uptake in tumor-infiltrating CD8^+^ T cells, causing lipid peroxidation and ferroptosis that impair their antitumor function. Conversely, CD36 genetic deletion or CD8^+^ T-cell ferroptosis inhibition can restore their antitumor activity [204]. NK cells, critical antitumor immune players, are also ferroptosis-susceptible, with their dysfunction linked to lipid peroxidation-driven oxidative stress. NRF2 signaling activation or ferroptosis inhibition has been shown to enhance NK cell survival and cytotoxicity in tumors [205]. B cells, key effectors of humoral immunity, show high sensitivity to GPX4 deficiency-induced ferroptosis due to their active lipid metabolism, potentially impairing antibody production and T-cell regulatory functions [203]. Additionally, ferroptotic cancer cells may cause DC dysfunction, characterized by impaired antigen cross-presentation and T-cell activation [206]. These findings underscore the complexity of ferroptosis regulation in tumor immunity. Future studies need to further clarify cell type-specific ferroptosis effects on various immune cells, and develop selective protective strategies to safeguard immune cell antitumor function while triggering tumor cell ferroptosis, thereby optimizing ferroptosis-targeted immunotherapy.

### Nanodrugs in Ferroptosis–Immunotherapy: Clinical Perspectives

Although developing anticancer drugs targeting ferroptosis–immunotherapy demands extended exploration, key advances in elucidating the ferroptosis–immune interplay and progress in nanodrug delivery technologies reveal promising clinical translation potential. Presently, few ferroptosis inducers have entered clinical trials. For example, Imetelstat modulates fatty acid metabolism to boost PUFA-PL synthesis, triggering lipid peroxidation and oxidative stress, thus inducing ferroptosis in AML cells. In a randomized Phase II trial in AML patient-derived xenografts, Imetelstat markedly reduced leukemic burden and prolonged survival [207]. In preclinical models, the GPX4 inhibitor cyst(e)inase combined with immunotherapy synergistically boosted T cell-mediated antitumor immunity and induced ferroptosis in cancer cells [31]. Moreover, zero-valent iron nanoparticles (ZVI-NPs) triggered mitochondrial dysfunction, oxidative stress, and lipid peroxidation in lung cancer cell lines, driving ferroptosis and remodeling the TME to deliver robust antitumor effects [208]. Notably, multiple FDA-approved drugs can induce ferroptosis, contributing to antitumor efficacy. For example, Sorafenib, approved for liver, kidney, and thyroid cancers, acts as a System Xc^−^ inhibitor and activates ferroptosis [209]. In breast cancer, combining lapatinib with the lysosome-disrupting agent siramesine disrupts iron metabolism and triggers lipid peroxidation [210]. Similarly, octreotide (approved for ovarian cancer) directly inhibits GPX4 to induce ferroptosis and enhance antitumor immunity [211, 212]. The antimalarial drug artesunate depletes GSH levels and elevates ROS in head and neck cancer cells, inducing ferroptosis and immune activation [213, 214].

Simultaneously, major progress occurred in nanodrug-based cancer immunotherapy, with several candidates in clinical trials. For instance, lipid nanoparticle (LNP)-encapsulated personalized mRNA cancer vaccines (mRNA-4157) produce robust T cell responses against mRNA-encoded neoantigens, displaying promising preclinical results. A Phase IIb trial is assessing mRNA-4157 plus pembrolizumab [215]. Similarly, personalized mRNA neoantigen vaccines (autogene cevumeran) delivered via lipoplex nanoparticles, administered intravenously to stimulate T cells in pancreatic cancer, have successfully completed Phase I trials [216]. Furthermore, CMP-001 (vidutolimod), a TLR9 agonist-encased virus-like particle, overcomes resistance to PD-1 blockade in advanced melanoma by inducing potent IFN responses and recruiting antitumor T cells, showing promising Phase II trial outcomes [217]. Another innovation employs platelet membrane-coated nanoparticles (PNP-R848) with a polylactic acid core loaded with the TLR7/8 agonist R848 to allow targeted delivery and immune activation. This system has secured FDA IND clearance and commenced clinical evaluation [218]. These advances underscore the promising clinical translation of ferroptosis–immunotherapy nanodrugs. Future efforts should accelerate clinical trials assessing safety/efficacy of ferroptosis–immunotherapy combinations to yield critical insights benefiting more cancer patients.

## Conclusion and Outlook

Recent advances in ferroptosis-inducing nanoplatforms integrated with immunomodulatory agents have catalyzed the development of synergistic ferroptosis–immunotherapy strategies. These approaches aim not only to eradicate primary tumors but also to activate systemic antitumor immunity, thereby suppressing metastatic and recurrent lesions. Such innovations establish a conceptual framework and potential paradigm shift for future cancer treatment, moving beyond conventional therapeutic modalities. The evolution of nanomaterial engineering has been instrumental in propelling tumor immunotherapy forward, with rational nanocarrier design emerging as a critical determinant of ferroptosis–immunotherapy efficacy. Firstly, current nanoplatform customization leverages diverse material compositions and structural configurations. Iron-based nanomaterials predominate due to their capacity to elevate intracellular iron levels, thereby potentiating Fenton reactions and generating cytotoxic ROS. Biomimetic systems utilizing bacterial membrane vesicles, tumor cell derivatives, or macrophage-inspired constructs demonstrate enhanced immune evasion, prolonged circulation, and tumor-homing capabilities. Hemoglobin-based carriers present dual advantages by simultaneously supplying oxygen for PDT and iron for ferroptosis induction, offering novel perspectives for drug delivery system design. Secondly, nanocarrier architecture dictates therapeutic cargo loading through mechanisms including hydrophobic interactions, *π*–*π* stacking, electrostatic adsorption, or covalent conjugation. Key physicochemical parameters—size, surface charge, morphology, amphiphilicity, and optical properties—critically influence biological stability, biodistribution, cellular internalization, and ultimately, therapeutic outcomes. Strategic modulation of these properties represents a pivotal optimization strategy. Thirdly, multifunctional nanocarriers engineered through chemical modifications enable: (1) targeted delivery via ligand–receptor interactions, (2) stimulus-responsive drug release under specific physicochemical cues, and (3) integrated bioimaging capabilities for treatment monitoring. Material selection further informs therapeutic potential, with Fenton-active metal-based nanomaterials, GSH-depleting nanozymes, and PUFA-loaded liposomes showing particular promise for ferroptosis potentiation. Lastly, the TME poses significant challenges through its immunosuppressive nature, hypoxia, antioxidant defenses, and dense ECM. While nanomaterial engineering addresses some barriers, comprehensive solutions require multimodal strategies to: (1) amplify immunogenic cell death, (2) reverse immunosuppression, (3) remodel hypoxic/ECM-rich TME features, (4) restore immune surveillance, (5) accelerate APC maturation, and (6) enhance tumor-infiltrating immune effector populations. Such integrated approaches maximize the therapeutic potential of ferroptosis–immunotherapy paradigms.

While nanomedicine-driven ferroptosis–immunotherapy has made substantial preclinical progress, critical challenges persist. First, biosafety remains paramount for clinical translation. Priority should be given to FDA-approved nanomaterials with established biocompatibility and biodegradability profiles (e.g., PEGylated liposomes, iron oxide nanoparticles, polymeric micelles) as foundational platforms [219]. Emerging materials (metals, MOFs, MPNs, bioinspired carriers) require comprehensive toxicological profiling beyond standard assessments, with emphasis on chronic effects—complement activation, hemolytic potential, inflammatory responses, and mitochondrial toxicity—to ensure clinical viability [220]. Despite clinical approvals for conventional nanotherapeutics (liposomal DOX, nab-paclitaxel) [221], no ferroptosis–immunotherapy nanocarriers have reached clinical testing, underscoring the need for optimized preclinical models to balance therapeutic efficacy with systemic safety. Moreover, while iron-based nanomaterials dominate ferroptosis induction strategies, their clinical implementation faces dual barriers: therapeutically effective intravenous doses (≥ 75 mg/kg) risk systemic iron overload and tissue hypersensitivity [222]. This necessitates combinatorial approaches to enhance tumor-selective ferroptosis sensitivity while minimizing metal exposure. Second, exploiting tumor-intrinsic H_2_O_2_ abundance for Fenton reactions remains constrained by reaction kinetics. Strategic integration of photothermal agents to locally enhance Fenton activity via hyperthermia [223], or Fe^3+^-chelating phenolic compounds (tannic/gallic acids) to stabilize Fe^2+^ and accelerate ROS generation, could mitigate these limitations. Current strategies over-rely on GSH/GPX4 inhibition; effective ferroptosis requires co-delivery of lipid peroxidation amplifiers (PUFAs) and ROS generators to overwhelm cellular antioxidant defenses. Third, in ICB-integrated regimens, conventional monoclonal antibody checkpoint inhibitors (anti-PD-1/PD-L1, anti-CTLA-4) face pharmacokinetic limitations—short plasma half-life and off-target distribution—that constrain therapeutic indices. Emerging solutions include engineering nanocarrier-compatible checkpoint inhibitors (antagonistic peptides, small molecules) and bispecific T-cell engagers that bridge tumor antigens to immune activation signals. Fourth, ferroptosis-induced ICD shows microenvironmental dependence; combining ferroptosis inducers with ICD amplifiers (PDT, hyperthermia, anthracyclines) creates positive feedback loops to enhance antitumor immunity. Notably, selective ferroptosis induction in immunosuppressive populations (M2 macrophages, MDSCs) via GPX4/ASAH2 inhibition must be balanced against ferroptosis vulnerability in CD8^+^ T-cells and Th subsets, requiring cell-specific targeting strategies [14]. Finally, monotherapeutic approaches—whether ICD enhancement, STING pathway activation, or TME normalization—often prove insufficient against established immunosuppressive networks. Sustained efficacy demands multimodal integration: coupling ferroptosis-mediated ICD with ICB to overcome T-cell exhaustion, while simultaneously reprogramming immunosuppressive niches through metabolic modulation and ECM remodeling. This combinatorial paradigm addresses both immunogenicity and immune evasion mechanisms, creating synergistic therapeutic windows unattainable through single-axis interventions.

The emerging field of ferroptosis–immunotherapy demonstrates significant therapeutic promise across malignancies including colorectal carcinoma, breast cancer, and melanoma. However, clinical translation progresses slowly due to heterogeneous ferroptosis responses across cancer molecular subtypes (e.g., P53/Ras-mutant variants), resulting in variable treatment efficacy [224]. Different cancer subtypes show significant heterogeneity in ferroptosis sensitivity due to variations in iron metabolism, lipid peroxidation regulation, and antioxidant defenses. Ovarian cancer tumor-initiating cells exhibit high ferroptosis-susceptibility from an iron-overloaded phenotype caused by elevated TFR1 and reduced FPN expression [225]. In gastric cancer, the mesenchymal subtype exhibits unique ferroptosis vulnerability mediated by very long-chain fatty acid protein 5 (ELOVL5) and fatty acid desaturase 1 (FADS1) dependent PUFA synthesis enhancement. Clear cell renal cell carcinoma establishes ferroptosis sensitivity through alkylglycerone-phosphate synthase (AGPS)-dependent PUFA-PL accumulation [14]. Notably, non-neuroendocrine small cell lung cancer outstrips its neuroendocrine counterpart in ferroptosis sensitivity, linked to distinct PL-synthase expression profiles and elevated PUFA-PL levels [226]. Furthermore, triple-negative breast cancer emerges as an ideal target for ferroptosis induction therapy due to unique metabolic traits: aberrant PUFA accumulation, elevated labile iron pool, and impaired GPX4-GSH defense [227]. This biological complexity necessitates tumor-specific therapeutic design. Notably, selective ferroptosis vulnerability in lung and breast carcinomas compared to normal epithelia enables tumor-selective cell death through precise therapeutic window identification, offering enhanced safety profiles [14]. Despite encouraging preclinical results with ferroptosis inducer–immunotherapy combinations, nanomedicine translation faces critical barriers. Firstly, structurally complex nanoplatforms encounter reproducibility and scalability challenges. Streamlined nanocarrier designs with well-defined physicochemical properties are essential to ensure manufacturing consistency and compliance with good production practices for clinical trials. Secondly, current preclinical evaluations rely predominantly on murine models that inadequately recapitulate human tumor pathophysiology. Developing clinically relevant models—such as patient-derived xenografts incorporating humanized immune systems—could bridge the translational gap by improving preclinical-to-clinical correlation. Thirdly, safety concerns persist regarding off-target effects: GPX4 inhibitors (common ferroptosis inducers) may disrupt neurological and renal homeostasis given GPX4's critical role in embryonic development and adult tissue maintenance [224]. Comprehensive toxicological profiling and structural optimization of ferroptosis inducers are required to mitigate systemic toxicity while preserving therapeutic activity. To advance clinical implementation, future efforts should prioritize: (1) rational combination therapies that amplify antitumor immunity while counteracting immunosuppressive networks, and (2) optimization of dosing regimens—including administration sequence, timing, and route—to maximize therapeutic synergy. Spatiotemporally controlled delivery systems could further enhance precision by coordinating ferroptosis induction with immune checkpoint blockade activation, potentially overcoming current limitations in treatment reliability. (3) Employ AI and machine learning to screen materials, optimize structure design, and enhance drug-loading efficiency, while guiding nanosystem development with high tumor-targeting capability and microenvironment adaptability through in vivo pathophysiological response simulations, thereby accelerating clinical translation. (4) Develop personalized treatment strategies based on patient-specific biomolecular features, optimizing ferroptosis–immunotherapy dosage and regimens to enhance efficacy and safety of tumor-subtype-specific treatments.

While nanomedicine-driven ferroptosis–immunotherapy remains investigational and unapproved for clinical use, its preclinical synergy demonstrates transformative potential. The convergence of ferroptosis biology, nanotechnology innovation, and clinical immunotherapy breakthroughs positions this paradigm as a promising frontier for oncology. By coupling tumor-selective ferroptosis induction with systemic immune activation, such strategies enable dual therapeutic action: direct ablation of primary lesions and immune-mediated suppression of metastases/recurrence. Overcoming current technical and translational barriers could establish ferroptosis–immunotherapy as a novel therapeutic modality. These nanotherapeutic platforms represent a conceptual leap in cancer immunotherapy, offering a multimodal weapon against treatment-resistant malignancies. With continued mechanistic elucidation and technological refinement, clinical translation of this approach may redefine therapeutic standards, providing novel avenues for precision oncology.
